# R0 resection following neoadjuvant PD-1 inhibition with paclitaxel-cisplatin is associated with improved overall survival in locally advanced and recurrent oral squamous cell carcinoma: multicenter retrospective cohort study

**DOI:** 10.1080/07853890.2026.2744077

**Published:** 2026-10-29

**Authors:** Qiwei Wang, Yan Liu, Liang Zuo, Shuichao Gao, Jiaming Chen, Lei Xue, Ping Li, Xiaoqing Lei, Jingguan Lin, Yaqian Han, Hekun Jin, Yujuan Zhou, Hao Tian

**Affiliations:** ^a^ Department of Head and Neck Surgery, Hunan Cancer Hospital, The Affiliated Cancer Hospital of Xiangya School of Medicine, Central South University, Changsha, Hunan Province, China; ^b^ Department of Pathology, Hunan Cancer Hospital, The Affiliated Cancer Hospital of Xiangya School of Medicine, Central South University, Changsha, Hunan Province, China; ^c^ Department of Maxillofacial-Head and Neck Surgery, Liuyang Hospital of Traditional Chinese Medicine, Changsha, Hunan Province, China; ^d^ Department of Otolaryngology, Liling Hospital of Traditional Chinese Medicine, Zhuzhou, Hunan Province, China; ^e^ Hunan Key Laboratory of Cancer Metabolism, Hunan Cancer Hospital, The Affiliated Cancer Hospital of Xiangya School of Medicine, Central South University, Changsha, Hunan Province, China; ^f^ Department of Head and Neck Radiotherapy, Hunan Cancer Hospital, The Affiliated Cancer Hospital of Xiangya School of Medicine, Central South University, Changsha, Hunan Province, China

**Keywords:** Neoadjuvant therapy, OSCC, overall survival, CPS

## Abstract

**Background:**

Locally advanced oral squamous cell carcinoma (LA-OSCC) has poor prognosis with standard therapy; recurrent disease is palliative. We compared neoadjuvant PD-1 inhibition plus paclitaxel-cisplatin followed by R0 resection ± radiotherapy versus non-surgical treatment in treatment-naive and recurrent LA-OSCC.

**Methods:**

This multicenter retrospective study (2021–2024) included 178 stage III–IVb LA-OSCC patients (43 recurrent, 135 treatment-naive) receiving neoadjuvant chemoimmunotherapy. The primary endpoint was overall survival (OS), analyzed using multivariable time-dependent Cox regression, propensity score matching (PSM), inverse probability of treatment weighting (IPTW), and landmark analyses

**Results:**

The overall response rate was 66.3%; the pathological complete response rate among 117 surgical patients was 35.0%. Responders had better OS than non-responders (HR 0.19, 95%CI 0.11–0.32, *p* < 0.001). Surgery was associated with improved OS versus non-surgical management (HR 0.23, 95%CI 0.13–0.39, *p* < 0.001), and the association remained consistent after multivariable adjustment (HR 0.29, 95%CI 0.16–0.52), PSM (47 pairs; HR 0.34, 95%CI 0.17–0.66), IPTW (HR 0.36, 95% CI 0.18–0.74), and 6-month landmark analysis (HR 0.22, 95%CI 0.13–0.39). Radiotherapy was also associated with improved OS (HR 0.43, 95%CI 0.21–0.87, *p* = 0.018); postoperative radiotherapy was associated with longer OS than surgery alone (HR 0.43, 95%CI 0.16–0.98, *p* = 0.047), and surgery was associated with improved OS in recurrent disease (HR 0.47, 95%CI 0.21–0.99, *p* = 0.049).

**Conclusion:**

In treatment-naive and recurrent LA-OSCC, neoadjuvant chemoimmunotherapy followed by response-directed R0 resection and risk-adapted radiotherapy was associated with improved OS. These retrospective associations warrant confirmation in prospective randomized trials.

## Introduction

Oral squamous cell carcinoma (OSCC) is the predominant malignancy of the head and neck. In 2022, an estimated 389,485 incident cases and 188,230 cancer-specific deaths were recorded worldwide [1]. The principal modifiable risk factors for OSCC are smoked tobacco, chronic heavy alcohol consumption, and areca-nut chewing, consequently, and a substantial proportion of patients with OSCC present with locally advanced disease [2]. The standard therapeutic approach for this cohort has consisted of surgical resection followed by adjuvant therapy, or definitive concurrent chemoradiotherapy. However, among patients with locally advanced OSCC (LA-OSCC) treated with this regimen, the 5-year survival rate remains suboptimal. Thus, there is an unmet need for novel therapeutic strategies that either improve survival outcomes or enable deintensification of the current standard of care.

Neoadjuvant therapy centered on PD-1 inhibitors has demonstrated substantial efficacy in multiple cancer types, including melanoma, non-small cell lung cancer, and prostate cancer [3–5]. Pembrolizumab or nivolumab, a monoclonal antibody targeting PD-1, has also been approved for the treatment of recurrent or metastatic head and neck squamous cell carcinoma (HNSCC), associated with a longer overall survival (OS) compared with chemotherapy [6,7]. While neoadjuvant immunotherapy has yielded considerable advantages over conventional chemotherapy in the treatment of HNSCC, objective response rate remains relatively low, largely constrained by substantial tumor burden and tumor heterogeneity. In contrast, neoadjuvant chemoimmunotherapy consisting primarily of PD-1 monoclonal antibodies plus platinum-based agents has effectively addressed this limitation of low objective response rates, with response rates consistently exceeding 60% across multiple clinical studies in HNSCC [8–11].

To date, in studies investigating neoadjuvant chemoimmunotherapy for OSCC, researchers have predominantly focused on treatment-naive patients, with limited attention paid to the ultra-advanced stage IVb population. Additionally, a subset of patients declines subsequent definitive treatment following neoadjuvant therapy due to various factors. In fact, recurrent OSCC patients may also be candidates for neoadjuvant chemoimmunotherapy, and a considerable proportion of stage IVb patients can still achieve R0 resection. Accordingly, we collected data from 178 consecutive patients with LA-OSCC across three distinct centers who received neoadjuvant therapy with a PD-1 inhibitor plus cisplatin and paclitaxel at our center between June 2021 and June 2024. This cohort included both recurrent and treatment-naive patients across stages III to IVb. The primary objective of this study was to evaluate the efficacy and feasibility of this therapeutic regimen, thereby providing a theoretical basis for the subsequent optimization of treatment strategies.

## Methods

### Evaluation and administration

Clinical data were retrospectively collected from the electronic medical record systems of the three participating centers (Hunan Cancer Hospital, Liuyang Hospital of Traditional Chinese Medicine and Liling Hospital of Traditional Chinese Medicine) for consecutive patients treated between June 2021 and June 2024. Extracted variables included demographic characteristics, tumor site and TNM stage, treatment details, pathological and radiological response assessments, and survival outcomes.

The PD-1 inhibitors used in this cohort reflected real-world prescribing practice in China and included zimberelimab, tislelizumab, pembrolizumab, penpulimab, and nivolumab, each administered in combination with paclitaxel (260 mg/m^2^) and cisplatin (75 mg/m^2^) every 3 weeks. No statistically significant difference in overall survival or pathological response was observed among patients treated with different PD-1 inhibitors.

Pathological evaluations were based on standard postoperative histopathological examination with serial sectioning of surgical specimens. Because no universally accepted histopathological regression grading system exists for oral squamous cell carcinoma, pathological response was classified using the following study-specific definitions, which categorize the histopathological findings of the resected specimen in conjunction with the radiological response: (i) pathological complete response (pCR), defined as the absence of residual invasive carcinoma on histological evaluation—Hematoxylin and eosin (H&E)-stained sections were systematically obtained during specimen processing, with a minimum of one section per 5 mm of the tumor’s greatest diameter to ensure adequate sampling; (ii) pathological partial response (pPR), defined as residual viable invasive carcinoma accompanied by substantial treatment-related regression in patients whose radiological assessment met RECIST v1.1 criteria for partial response; (iii) pathological stable disease (pSD), defined as residual viable invasive carcinoma with little or no histological evidence of regression, corresponding to radiological stable disease (neither sufficient shrinkage to qualify for PR nor sufficient increase to qualify for PD); and (iv) pathological progressive disease (pPD), defined as viable tumor extending beyond the pretreatment disease extent, corresponding to radiological progressive disease. Radiological assessments were performed using contrast-enhanced magnetic resonance imaging (MRI) and contrast-enhanced computed tomography (CT), and radiological response was classified according to RECIST v1.1 (CR, PR, SD, PD). Patients who did not undergo surgery were evaluated radiologically only. Because few patients had pPD and both pPD and PD indicated disease progression, these categories were pooled for analysis and are collectively referred to as PD.

Since intraoperative rapid frozen section analysis generally ensured negative surgical margins, the presence of pathological extra nodal extension (ENE+) served as the primary risk stratification factor for postoperative radiotherapy. For surgical patients: (1) pCR and pPR (without ENE+): 60 Gy; 30 fractions. (2) pSD, PD or pPR (with ENE+): 66 Gy plus concurrent chemotherapy (cisplatin: 40 mg/m^2^ weekly); 33 fractions. For non-surgical patients: CR/PR/SD/PD: 66 Gy plus concurrent chemotherapy (cisplatin: 100 mg/m^2^ every 3 weeks). Radiotherapy using intensity-modulated radiotherapy (IMRT) was uniformly administered across participating centers. The radiation treatment fields encompassed the primary tumor bed and the cervical region. For patients with midline tumors, such as those located in the oral tongue or floor of the mouth, bilateral neck irradiation was administered. Postoperative radiotherapy was initiated within 5 to 10 weeks after surgery in all surgical patients, all of whom completed the prescribed radiation dose. The pathological grading characteristics of the 48 patients who received postoperative radiotherapy are presented in Table S1.

Standard pre-operative optimization included nutritional support, dental clearance, and management of comorbidities. All surgery patients involve R0 resection with a minimum macroscopic margin of 0.5–1.0 cm beyond the post-neoadjuvant therapy tumor boundary. Specimens were sampled with a minimum of one section per 5 mm of the greatest tumor diameter. In patients with radiologically or cytologically suspected cervical lymph node metastasis, concurrent neck dissection of the involved anatomical compartments should be performed to achieve Enbloc resection. Intraoperative defect reconstruction is determined by the extent of tissue loss and may include primary closure, biologic mesh repair, pedicled flaps, or free flap transfer. Post-treatment surveillance consisted of clinical exam and contrast-enhanced MRI/CT.

During pre-operative counseling or at the time of the pre-treatment pathological biopsy, it was explicitly communicated to every patient that the resected lesions, tissues, and specimens could be subjected to pathological examination, cytological examination, and genetic testing, and each patient signed a written informed consent form authorizing these examinations. All operations were performed by board-certified head-and-neck surgeons with ≥10 years of experience; radiotherapy was delivered by specialist radiation oncologists. All centers followed shared principles for response assessment, intraoperative frozen-section margin confirmation, target delineation, radiotherapy dose prescription, and initiation of postoperative radiotherapy. An a priori protocol was developed and is available on reasonable request.

Overall survival was calculated from the date of neoadjuvant therapy initiation to the date of death from any cause or last follow-up.

### Statistical analysis

The Kruskal-Wallis test was employed to assess differences in tumor volume reduction rates across multiple subgroups. Categorical variables were analyzed using the chi-square test to evaluate potential associations between binary factors. The difference in OS between different subgroup, Kaplan-Meier survival curves and log-rank tests were employed. P-value less than 0.05 was considered statistically significant, indicating a significant difference or association in the analyzed data. To avoid overfitting, the multivariable Cox model was restricted to prespecified pretreatment baseline prognostic factors: recurrent disease at baseline, T stage (collapsed as T0–T2/T3/T4 because of the small numbers of T0–T2 cases), N stage, and primary tumor site. Overall AJCC stage was not entered simultaneously because it is a deterministic combination of T and N stages. Treatment response category was not included because it is a post-treatment variable and a potential mediator of the association between subsequent treatment and survival. Surgery and radiotherapy were modeled as time-dependent covariates in the multivariable Cox model to account for their occurrence after the start of follow-up. The proportional hazards assumption was assessed using Schoenfeld residuals.

To mitigate treatment-selection bias arising from the non-randomized treatment allocation, we performed propensity score analyses. Propensity scores for receiving surgery were estimated using a logistic regression model incorporating pretreatment baseline covariates (age, sex, smoking, alcohol consumption, betel-nut chewing, recurrence status, clinical T and N stage, and primary tumor site). One-to-one nearest-neighbor matching without replacement was conducted with a caliper of 0.2 standard deviations of the logit of the propensity score; covariate balance was assessed using standardized mean differences (SMD), with SMD < 0.1 prespecified as indicative of good balance. Survival in the matched cohort was compared using the log-rank test. As a complementary analysis, inverse probability of treatment weighting (IPTW) was applied using stabilized weights trimmed at the 1st and 99th percentiles, followed by a weighted Cox model with robust sandwich variance estimation. To account for potential immortal time bias arising from the interval between neoadjuvant therapy initiation and subsequent surgery or radiotherapy, landmark analyses were performed with the landmark set at 6 months for surgery comparisons and 9 months for radiotherapy comparisons (both later than the latest protocol-plausible treatment dates). All computational and statistical analyses were conducted using R-4.2.2.

### Combined positive score (CPS) assessment

PD-L1 Combined Positive Score (CPS) was quantified using immunohistochemical analysis, with the specific methodology as follows: pre- neoadjuvant biopsy samples were tested using the Dako22C3 pharmDx^™^ companion diagnostic kit. Immunohistochemical results were evaluated under a light microscope at ×100 magnification, and the CPS values were reported on a scale of 0–100. Two types of archived, formalin-fixed paraffin-embedded (FFPE) human specimens, all obtained during routine clinical care, were used in this study: (i) pre-treatment biopsy specimens of the primary tumor, used for PD-L1 CPS immunohistochemistry (Dako 22C3 pharmDx) as described above; and (ii) surgical resection specimens, used for postoperative histopathological examination. The CPS assessment comprised specimens from 12 patients in the initial exploratory analysis, obtained between September 2022 and October 2023, and from 20 additionally accrued surgical patients in the expanded analysis (32 patients in total), collected between August 2024 and March 2025.

## Results

### Patient characteristics

From June 2021 to June 2024, a total of 178 patients across three distinct centers with locally oral squamous cell carcinoma (LA-OSCC) were enrolled in this study. The cohort included 43 patients (24.2%) with recurrent disease and 135 treatment-naïve patients (75.8%). The median age was 52 years (range, 27–73), and the majority of patients were male (168 [94.4%]). A history of smoking was reported in 115 patients (64.6%), alcohol consumption in 80 (44.9%), and betel nut chewing in 19 (10.7%). Primary tumor site distribution included the tongue (including the floor of the mouth), gingiva, buccal mucosa, and hard palate. Additionally, 4 recurrent patients presented with metastatic lymph nodes alone. Pretreatment staging revealed that all patients had American Joint Committee on Cancer (AJCC) stage III or higher disease (stage III: 43 [24.16%]; stage IVa: 96 [53.93%]; stage IVb: 39 [21.91%]). The primary tumor (T) and nodal (N) classifications were as follows: T-stage: T0 (4 [2.25%]), T1 (1 [0.56%]), T2 (12 [6.74%]), T3 (77 [43.26%]), T4 (84 [47.19%]); N-stage: N0 (56 [31.46%]), N1 (41 [23.03%]), N2 (72 [40.45%]), N3 (9 [5.06%]). The anatomical distribution of tumors was as follows: buccal/palate (53 [29.78%]), gingiva (32 [17.98%]) and tongue (93 [52.25%]). The demographics and clinical characteristics are summarized in Table 1.

**Table 1.** Clinical characteristics.

Characteristics	Patients (*n* = 178)
Age, median (range; years)	52 (27–73)
**Sex**	
female	10 (5.6%)
male	168 (94.4%)
**Smoking**	
Yes	115 (64.6%)
No	63 (35.4%)
**Drinking Alcohol**	
Yes	80 (44.9%)
No	98 (55.1%)
**Chewing Betel**	
Yes	19 (10.7%)
No	159 (89.3%)
**Pretreatment clinical T-stage**	
T0	4 (2.25%)
T1	1 (0.56%)
T2	12 (6.74%)
T3	77 (43.26%)
T4	84 (47.19%)
**Pretreatment clinical N-stage**	
N0	56 (31.46%)
N1	41 (23.03%)
N2	72 (40.45%)
N3	9 (5.06%)
**AJCC stage**	
III	43 (24.16%)
IVa	96 (53.93%)
IVb	39 (21.91%)
**Recurrent disease**	
Yes	43 (24.2%)
No	135 (75.8%)
**Anatomical regions**	
Buccal	53 (29.78%)
Gingiva/palate	32 (17.98%)
Tongue	93 (52.25%)
**Centers**	
Center1	106 (59.55%)
Center2	37 (20.79%)
Center3	35 (19.66%)
**Efficacy evaluation**	
SD	23 (12.92%)
PD	19 (10.67%)
PR	17 (9.55%)
CR	5 (2.81%)
pCR	41 (23.03%)
pPR	55 (30.9%)
pSD	18 (10.11%)

### Treatment characteristics

All patients underwent 2 to 5 cycles of neoadjuvant therapy (Figure 1A). The overall response rate was 66.29% (Figure 1E). Specifically, within the recurrence LA-OSCC subgroup, the response rate was 41.86% (Figure 1D), whereas in the non - recurrence LA-OSCC subgroup, the response rate reached 74.07% (Figure 1C). 117 patients underwent surgical treatment on days 21 to 35 following the completion of the last treatment cycle. The surgical approach comprises either resection of the primary site combined with neck dissection or neck dissection alone. 61 patients did not undergo surgery. In the surgery group, the response rate was 82.05%, the pCR rates was 35.04% (Figure 1F), 48 patients received postoperative radiotherapy. The Sankey diagram illustrates the therapeutic trajectory of the 178 patients enrolled in this study (Figure 1G). Additionally, 52 patients declined any further treatment following neoadjuvant therapy. Among these, 15 patients refused subsequent treatment due to disease progression, 9 declined for personal reasons, and the remaining 28 were unable to pursue further therapy owing to financial constraints.

**Figure 1.**
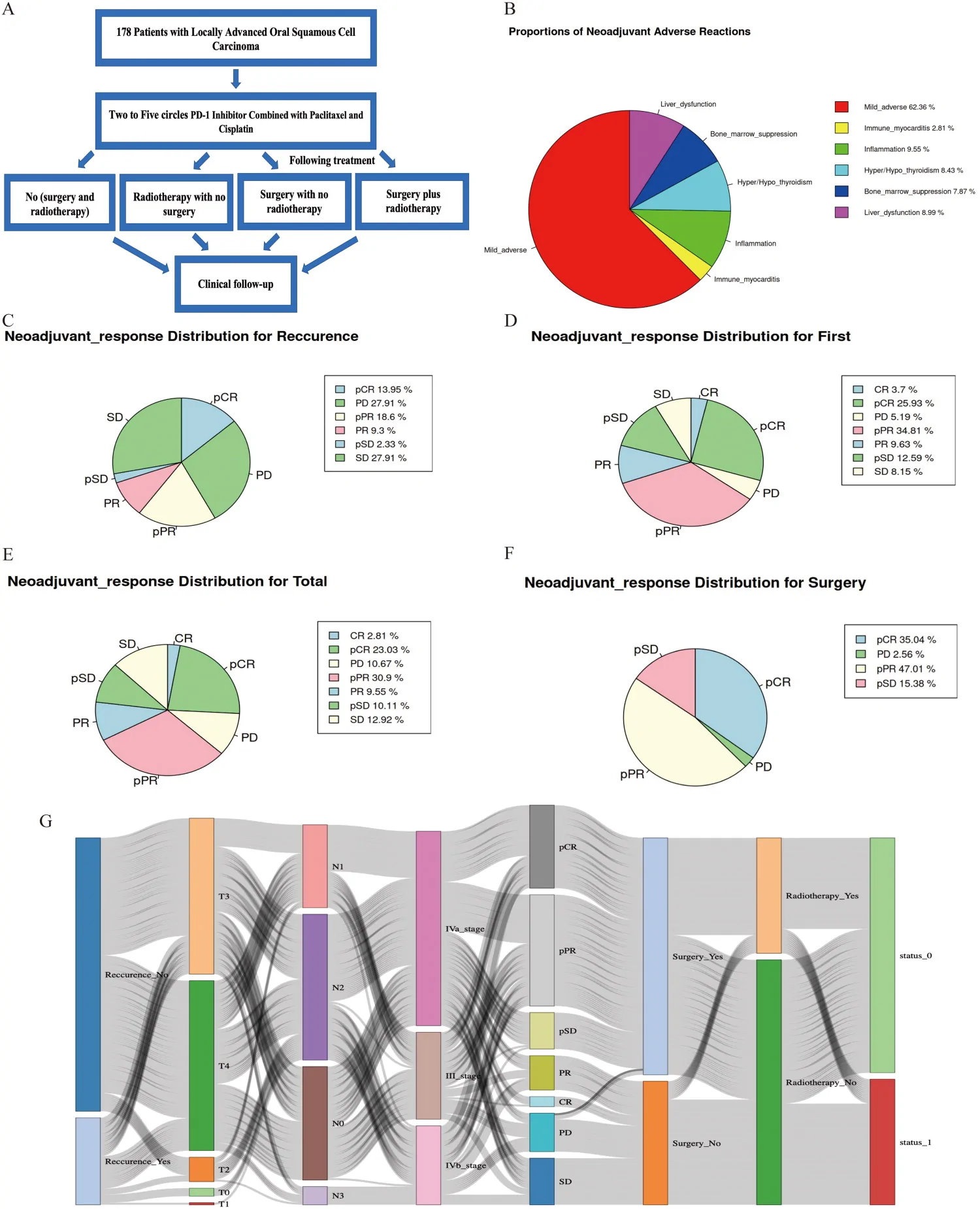
Workflow, adverse events and tumor response of this study. (A) Workflow of this study. (B) Adverse event proportion plot of 178 included patients. (C) Tumor response for recurrence-LA-OSCC population; *n* = 43. (D) Tumor response for treatment naive LA-OSCC population; n = 135. (E) Tumor response for total LA-OSCC population; *n* = 178. (F) Tumor response for surgery LA-OSCC population, *n* = 117. (G) Sankey diagram of the treatment course of the 178 included patients; status 0 = alive, status 1 = dead.

### Safety

Treatment-related adverse events (TRAEs) are presented in Figure 1B. According to the CTCAE v5.0 grading criteria, all patients who received neoadjuvant therapy experienced TRAEs, with the majority being grade 1–2 in severity; only one patient developed a grade 3 TRAE. The most common TRAEs were mild and included fatigue, nausea, among others, accounting for 62.36% of events. Immune-related myocarditis was evaluated according to the CTCAE definitions. Serum troponin I/T and 12-lead electrocardiography were routinely monitored before each neoadjuvant cycle. Patients with newly elevated troponin underwent echocardiography, and cardiac magnetic resonance imaging and cardiology consultation were performed when clinically indicated. Among 13 patients with unexplained post-treatment troponin elevation, 5 (2.81%) met the criteria for clinically diagnosed (probable/definite) immune-related myocarditis; the remainder were classified as possible myocardial injury. Four events were grade 1–2, and one was grade 3, occurring at the end of the second neoadjuvant cycle; the latter resolved after corticosteroid pulse therapy and intravenous immunoglobulin, and the patient ultimately underwent surgery on day 33 after completion of the second cycle. Bone marrow suppression was observed in 14 patients (7.87%), characterized by reductions in platelet count, hemoglobin levels, and neutrophil counts. An additional 17 patients (9.55%) exhibited signs of inflammation. Thyroid dysfunction was noted in 15 patients (8.43%), and abnormal liver function in 16 patients (8.99%). All patients experienced resolution or alleviation of adverse reactions following symptomatic and supportive treatment. No previously unknown or unexpected complications were observed. Importantly, no TRAEs led to delays, discontinuation, or dose reductions of neoadjuvant therapy, and no treatment-related surgical delays were observed.

### Baseline characteristics of treatment group

To evaluate the impact of baseline clinical characteristics on the efficacy of neoadjuvant therapy and subsequent treatment decisions, we conducted comparative analyses of demographic and clinical features across the following subgroups. The results demonstrated that disease recurrence status at initial diagnosis significantly influenced therapeutic decision-making, likely attributable to the more aggressive tumor biology and poorer treatment response observed in recurrent cases. However, no statistically significant associations were observed between smoking history, gender, alcohol consumption, AJCC clinical stage, T stage, N stage, or betel chewing habit with subsequent treatment choices. Of particular note, patients exhibited varying degrees of surgical acceptance depending on tumor location. Those with buccal mucosa or tongue cancers demonstrated higher surgical acceptance rates, potentially attributable to the relatively minor functional and aesthetic impacts of surgery in these sites. Conversely, patients with gingival and hard palate malignancies showed lower surgical and radiotherapeutic willingness, likely due to greater anticipated postoperative functional and cosmetic impairments. Patients with recurrent tumor demonstrated significantly lower adherence to subsequent therapies following neoadjuvant treatment compared to treatment-naive LA-OSCC patients (*p* < 0.001, chi-square test, p-values were adjusted for multiple comparisons). This disparity in treatment continuation rates may be attributable to multifactorial determinants, including the heightened psychological burden associated with disease recurrence, or socioeconomic barriers to care (**Table 2**).

**Table 2.** Group characteristics.

	Neither Surgery nor Radiotherapy (*N* = 52)	Radiotherapy alone (*N* = 9)	Surgery alone (*N* = 69)	Surgery plus radiotherapy (*N* = 48)	Chi-square P-value
**Gender**					
female	5 (9.6%)	0 (0%)	2 (2.9%)	3 (6.3%)	0.376
male	47 (90.4%)	9 (100%)	67 (97.1%)	45 (93.8%)	
**Clinical_Staging**					
III_stage	13 (25.0%)	1 (11.1%)	17 (24.6%)	12 (25.0%)	0.647
IVa_stage	24 (46.2%)	6 (66.7%)	37 (53.6%)	29 (60.4%)	
IVb_stage	15 (28.8%)	2 (22.2%)	15 (21.7%)	7 (14.6%)	
**Recurrent disease**					<0.001
No	30 (57.7%)	5 (55.6%)	56 (81.2%)	44 (91.7%)	
Yes	22 (42.3%)	4 (44.4%)	13 (18.8%)	4 (8.3%)	
**T_stage**					0.749
T0	0 (0%)	0 (0%)	2 (2.9%)	2 (4.2%)	
T1	0 (0%)	0 (0%)	1 (1.4%)	0 (0%)	
T2	3 (5.8%)	2 (22.2%)	3 (4.3%)	4 (8.3%)	
T3	24 (46.2%)	3 (33.3%)	29 (42.0%)	21 (43.8%)	
T4	25 (48.1%)	4 (44.4%)	34 (49.3%)	21 (43.8%)	
**N_stage**					0.0971
N0	21 (40.4%)	0 (0%)	23 (33.3%)	12 (25.0%)	
N1	7 (13.5%)	3 (33.3%)	15 (21.7%)	16 (33.3%)	
N2	19 (36.5%)	6 (66.7%)	29 (42.0%)	18 (37.5%)	
N3	5 (9.6%)	0 (0%)	2 (2.9%)	2 (4.2%)	
**Tumor_site**					0.0742
Buccal	14 (26.9%)	2 (22.2%)	26 (37.7%)	11 (22.9%)	
Gingiva-palate	12 (23.1%)	4 (44.4%)	11 (15.9%)	5 (10.4%)	
Tongue	26 (50.0%)	3 (33.3%)	32 (46.4%)	32 (66.7%)	
**Smoking**					0.712
No	20 (38.5%)	4 (44.4%)	21 (30.4%)	18 (37.5%)	
Yes	32 (61.5%)	5 (55.6%)	48 (69.6%)	30 (62.5%)	
**Drinking**					0.164
No	26 (50.0%)	5 (55.6%)	34 (49.3%)	33 (68.8%)	
Yes	26 (50.0%)	4 (44.4%)	35 (50.7%)	15 (31.3%)	
**Betel_chewing**					0.615
No	46 (88.5%)	9 (100%)	60 (87.0%)	44 (91.7%)	
Yes	6 (11.5%)	0 (0%)	9 (13.0%)	4 (8.3%)	

### Survival outcomes

Among 178 patients, 62 deaths (34.8%) occurred during a median observed follow-up of 1.35 years. The results demonstrated that 46 patients achieved a pCR or CR, 72 patients exhibited a pPR or PR, and 41 patients had pSD or SD, however, PD was observed in 19 patients. Kaplan-Meier analysis revealed statistically significant differences in overall survival (OS) among the four response groups: pCR/CR demonstrated the most favorable outcomes, followed by those with pPR/PR, pSD/SD and PD (pCR/CR vs PD: *p* < 0.001; pCR/CR vs pPR/PR: *p* = 0.005; pCR/CR vs pSD/SD: *p* < 0.001; pPR/PR vs PD: *p* < 0.001; pPR/PR vs pSD/SD: *p* < 0.001; pSD/SD vs PD: *p* = 0.423; Figure 2A). Notably, when stratified by clinical benefit, the pCR/CR and pPR/PR group (responders) had significantly longer survival than the pSD/SD and PD group (non-responders) (*p* < 0.001; HR = 0.19; 95% CI: 0.11–0.32; Figure 2B). Surgical resection remains a cornerstone of treatment for locally advanced HNSCC. Undergoing total surgical resection following neoadjuvant therapy was significantly associated with improved OS compared with non-surgical management (*p* < 0.001; HR = 0.23; 95% CI: 0.13–0.39; Figure 2C). Further analysis revealed that surgical resection was associated with superior survival versus non-surgical management in the response group (*p* = 0.0033; HR = 0.3; 95% CI: 0.13–0.7; Figure 2D); while a trend toward improved OS was observed with surgery, this difference did not reach statistical significance in the non-responder’s group (*p* = 0.164; HR = 0.58; 95% CI: 0.26–1.3; Figure S1A).

**Figure 2.**
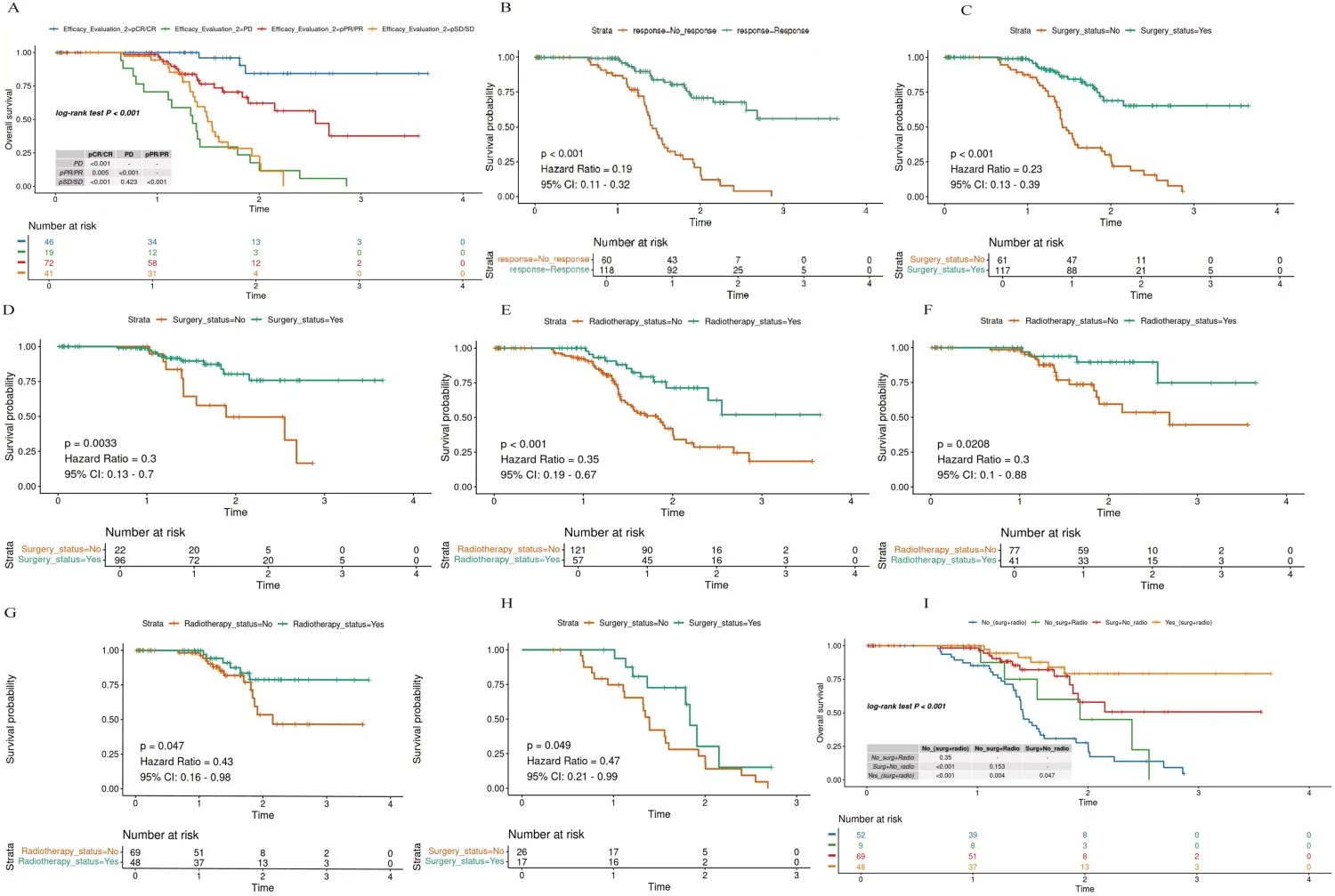
Neoadjuvant therapy contribute to the better overall survival of LA-OSCC. (A) Different tumor response group was associated with different OS, pairwise log-rank P values, with Holm-Bonferroni-adjusted P values in parentheses: (pCR/CR vs PD: *p *< 0.001; adjusted *p *< 0.001; pCR/CR vs pPR/PR: *p *= 0.002, adjusted *p *= 0.009; pCR/CR vs pSD/SD: *p *< 0.001, adjusted *p *< 0.001; pPR/PR vs PD: *p *< 0.001, adjusted *p *< 0.001; pPR/PR vs pSD/SD: *p *= 0.0003, adjusted *p *= 0.006; pSD/SD vs PD: *p *= 0.423, adjusted *p *= 0.453). (B) Response group shows better OS than Non response group; *p *< 0.001; HR = 0.19; 95% CI: 0.11–0.32. (C) Surgery group shows better OS than Non surgery group; *p *< 0.001; HR = 0.23; 95% CI: 0.13–0.39. D) In response group, patients receive surgery shows better OS than non-surgical treatment patients; *p *= 0.0033; HR = 0.3; 95% CI: 0.13–0.7. (E) Radiotherapy group shows better OS than Non radiotherapy group; *p *< 0.001; HR = 0.35; 95% CI: 0.19–0.67. (F) In response group, patients receive radiotherapy shows better OS than non-radiotherapeutic treatment patients; *p *= 0.0208; HR = 0.3; 95% CI: 0.1–0.88. (G) In surgery group, patients receive post-surgery radiotherapy shows better OS than non-radiotherapeutic treatment patients; *p *= 0.047; HR = 0.43; 95% CI: 0.16–0.98. (H) In recurrence LA-OSCC group, patients receive surgery shows better OS than non-surgical treatment patients; *p *= 0.049; HR = 0.47; 95% CI: 0.21–0.99. (I) (Exploratory) Pairwise log-rank P values, with Holm-Bonferroni-adjusted P values in parentheses: Non_(surg + radio) vs Non_surg + Radio: *p *= 0.35, (adjusted *p *= 0.415); Non_(surg + radio) vs Surg + Non_radio: *p *< 0.001, (adjusted *p *< 0.001); Non_(surg + radio) vs Yes_(surg + Radio): *p *< 0.001, (adjusted *p *< 0.001); Non_surg + Radio vs Surg + Non_radio: *p *= 0.153, (adjusted *p *= 0.375); Non_surg + Radio vs Yes_(surg + Radio): *p *= 0.004, (adjusted *p *= 0.0088); Surg + Non_radio vs Yes_(surg + Radio): *p *= 0.047, (adjusted *p *= 0.1295).X-axis: Time-years; Y-axis: OS.

Radiotherapy also is firmly established as the backbone of treatment for locally advanced HNSCC. In our cohort, receiving radiotherapy was significantly associated with improved OS compared with non-radiotherapy management (*p* < 0.001; HR = 0.35; 95% CI: 0.19–0.67; Figure 2E). Meanwhile, post-operation radiotherapy has been widely reported to benefit survival in locally advanced HNSCC [12], we specifically evaluated its prognostic impact in our cohort. Postoperative radiotherapy was associated with significantly longer OS compared with surgery alone (*p* = 0.047; HR = 0.43; 95% CI: 0.16–0.98; Figure 2G). This association was consistent across response subgroups (*p* = 0.0208; HR = 0.3; 95% CI: 0.1–0.88; Figure 2F), but no survival advantage was observed in the non-response and non-surgery groups (Non response group: *p* = 0.31; HR = 0.67; 95% CI: 0.31–1.5; Figure S1B. Non surgery group: *p* = 0.35; HR = 0.66; 95% CI: 0.28–1.6; Figure S1C), potentially attributable to higher tumor burden in this subgroup.

It is noteworthy that surgery remains a particularly critical therapeutic modality among the 43 patients with recurrent LA-OSCC (*p* = 0.049; HR = 0.47; 95% CI: 0.21–0.99; Figure 2H). Although no significant survival difference was observed between radiotherapy and non-radiotherapy groups, radiotherapy still serves as an effective short-term disease control strategy (*p* = 0.131; HR = 0.48; 95% CI: 0.18–1.3; Figure S1D). Notably, among the 13 patients who achieved PCR and received postoperative radiotherapy, no clinical deaths were observed (*p* = 0.15; Figure S1E).

Based on these findings, the exploratory four-group Kaplan-Meier analysis showed that the trimodality approach-neoadjuvant therapy followed by surgery and postoperative radiotherapy-was associated with the numerically best OS (pairwise log-rank P values, with Holm-Bonferroni-adjusted P values in parentheses: Non_(surg + radio) vs Non_surg + Radio: *p* = 0.35, (adjusted *p* = 0.415); Non_(surg + radio) vs Surg + Non_radio: *p* < 0.001, (adjusted *p* < 0.001); Non_(surg + radio) vs Yes_(surg + Radio): *p* < 0.001, (adjusted *p* < 0.001); Non_surg + Radio vs Surg + Non_radio: *p* = 0.153, (adjusted *p* = 0.375); Non_surg + Radio vs Yes_(surg + Radio): *p* = 0.004, (adjusted *p* = 0.0088); Surg + Non_radio vs Yes_(surg + Radio): *p* = 0.047, (adjusted *p* = 0.1295). Figure 2I). Cao et al. [13] reported that survival outcomes in locally advanced HNSCC varied significantly by anatomical subsite, our data found no statistically significant association between primary tumor location and OS (*p* = 0.19; Figure S1F).

We performed multivariate Cox regression including only pretreatment covariates plus surgery and radiotherapy, found surgery remained independently associated with improved overall survival (HR 0.29, 95% CI 0.16–0.52, *p* < 0.001), as did radiotherapy (HR 0.43, 95% CI 0.21–0.87, *p* = 0.018) and recurrent disease (HR 2.06, 95% CI 1.15–3.69, *p* = 0.016) (Table 3). The baseline model including all pretreatment variables yielded similar conclusions, that recurrence status was significantly associated with OS (Tables 2). After 1:1 propensity score matching (47 pairs), covariate balance improved substantially between the surgical and non-surgical groups, with the maximum absolute SMD reduced from 0.65 before matching to <0.30 after matching; however, not all covariates met the prespecified SMD <0.10 threshold (Figure S2A). Surgery remained associated with significantly improved OS in the matched cohort (HR = 0.34, 95% CI 0.17–0.66, *p* = 0.0015; Figure S2B). This association was confirmed by IPTW analysis of the full cohort (HR = 0.36, 95% CI 0.18–0.74, *p* = 0.0053; Figure S3A), and a consistent association was observed for radiotherapy in an IPTW sensitivity analysis (HR = 0.53, 95% CI 0.28–0.99, *p* = 0.045; Figure S3B). We also performed landmark analyses with the landmark set at 6 months for surgery comparisons and 9 months for radiotherapy comparisons. Patients who died or were censored before the landmark were excluded and the time origin was reset. The results were essentially unchanged (Figure S4A–D).

**Table 3.** Multivariable cox regression analysis.

Variable	HR	P value	Lower 95%CI	Upper 95%CI
**Recurrent disease**				
Yes VS No	2.06	**0.016**	1.15	3.69
**T_stage**				
T3 VS T0-T2	0.49	0.162	0.18	1.34
T4 VS T0-T2	0.6	0.287	0.24	1.53
**N_stage**				
N1 VS N0	1.15	0.723	0.54	2.45
N2 VS N0	1.36	0.401	0.66	2.81
N3 VS N0	1.12	0.85	0.34	3.47
**Surgery status**				
Yes VS No	0.29	**<0.001**	0.16	0.52
**Radiotherapy status**				
Yes VS No	0.43	**0.018**	0.21	0.87
**Tumor site**				
Gingiva_palate VS Buccal	0.57	0.175	0.25	1.29
Tongue VS Buccal	0.62	0.111	0.34	1.12

Bold values indicate *p*-values that are statistically significant (p < 0.05)

### Efficacy correlation

The waterfall heatmap demonstrates the relationship between post-neoadjuvant therapy tumor volume reduction and baseline clinical characteristics, revealing differential response patterns across demographic subgroups (Figure 3A). To investigate the factors influencing neoadjuvant therapy outcomes, we performed Kruskal-Wallis tests to assess the association between baseline clinical characteristics and changes in tumor volume on imaging in our cohort. We observed that pCR patients demonstrated significantly greater tumor volume reduction compared to PR patients (Figure 3B, *p* < 0.001). Patients who experienced recurrence demonstrated poorer responses to neoadjuvant treatment (Figure 3C, *p* < 0.001), suggesting that these tumors possess heightened aggressiveness and a more robust barrier effect, potentially impeding drug penetration or immune activation [14]. Additionally, tumors arising in the buccal mucosa, hard palate, and gingiva exhibited a smaller reduction in tumor volume on imaging compared with those located in the tongue (including the floor of the mouth) (Figure 3D, *p* = 0.024), possibly due to their unique anatomical constraints, higher intrinsic malignancy, and reduced lymphatic drainage efficiency [15].

**Figure 3.**
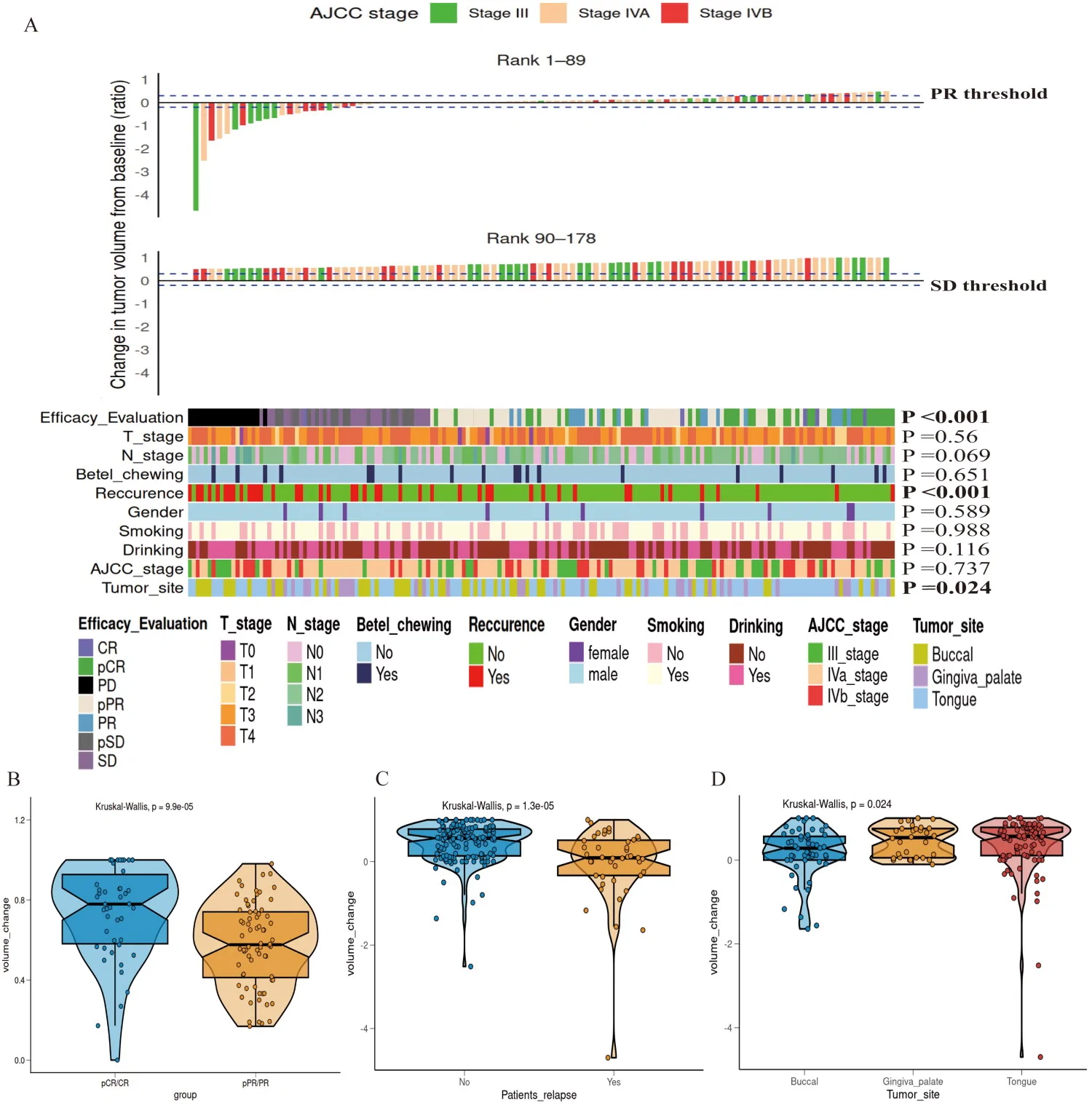
Tumor volume change and association with clinical characteristics. (A) Waterfall heatmap for relationship between tumor volume change and clinical characteristics. (B) Tumor volume change between pCR/CR group and pPR/PR group; P = 9.9*10^−5^. (C) Tumor volume change between recurrence LA-HNSCC group and no recurrence LA-OSCC group; P = 1.3*10^−5^. (D) Tumor volume change between buccal, gingiva_palate and tongue; P= 0.024.

### CPS evaluation

We randomly selected 3 patients from each of the four groups (meeting statistical requirements) for Combined Positive Score (CPS) assessment to investigate whether the CPS could predict pathological response. Magnetic resonance imaging (MRI) demonstrated the pre- and post-neoadjuvant therapy changes in one representative patient who achieved PCR and another with PD (Figure 4A and B). Results showed that among the 3 patients achieving pCR, all CPS < 1. Of the 3 patients with pPR, 1 had a CPS between 1 and 5, 1 had a CPS of 40, and 1 had a CPS of 60. Of the 3 patients with pSD, 3 had CPS values of 40, 30, and 20, respectively. For the 3 patients with PD, the CPS values were 1–5, 5, and 15. No statistically significant difference in CPS was observed among the response (pCR & pPR) and no response (pSD and PD) group (Figure 4C). We used immunohistochemistry to demonstrate the pathological features of 12 of these patients (Figure 4D–G). We also assessed CPS from 20 consecutively enrolled recent surgical patients (10 pCR, 10 pSD). In the pooled cohort of 32 patients, no statistically significant association between CPS and pathological response was observed (CPS ≤ 5 vs > 10: odds ratio = 1.29, Fisher’s exact *p* = 1.000). Notably, 5 of 10 patients achieving pCR in the expanded cohort had a CPS ≤ 5, including 2 with a CPS of 0.

**Figure 4.**
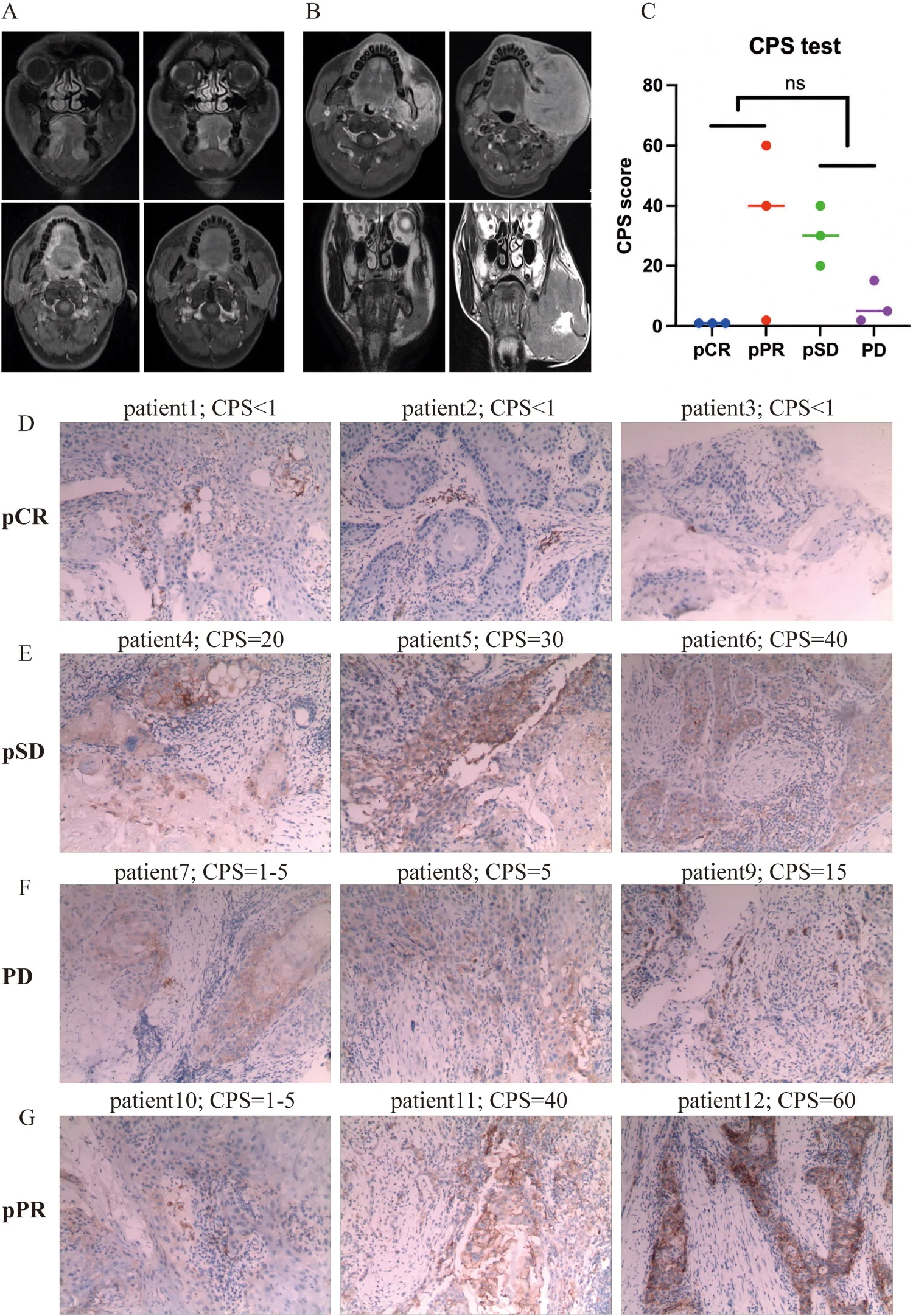
CPS evaluation in our cohort. (A) MRI before surgery demonstrates near-complete clinical regression of primary lesion. Top, before neoadjuvant chemoimmunotherapy; Down, after neoadjuvant chemoimmunotherapy. (B) MRI before surgery demonstrates progress of primary lesion. Top, before neoadjuvant chemoimmunotherapy; Down, after neoadjuvant chemoimmunotherapy. (C) The bar graph demonstrates no statistically significant difference in the CPS among the response and no response groups. (D-G) Histopathologic images of post-treatment resection specimens and their corresponding CPS values, (D): pCR; (E): pSD; (F): PD; (G): pPR; microscope at ×100 magnification.

## Discussion

The current standard treatment paradigm for LA-OSCC involves surgical resection followed by adjuvant radiotherapy [16]. However, this approach continues to face significant challenges, including high recurrence rates and mortality, with 5-year overall survival rates stagnating below 50% in most real-world datasets [17]. These persistent unmet clinical needs underscore the imperative for novel therapeutic strategies to fundamentally transform outcomes in this patient population.

Over the past decade, immunotherapy has revolutionized the treatment paradigm for solid tumors, with remarkable achievements in R/M OSCC establishing pembrolizumab as a first-line standard [18]. Multiple phase II clinical trials have now demonstrated both the promising efficacy and favorable safety profile of PD1 inhibitor as a neoadjuvant therapy for LA-OSCC [19,20]. The latest landmark KEYNOTE-689 trial demonstrated that a multimodal approach combining neoadjuvant pembrolizumab followed by surgery, radiotherapy, and adjuvant pembrolizumab maintenance (experimental arm) significantly improved EFS compared to standard therapy (surgery plus radiotherapy alone), notably, patients with CPS ≥10 showed a OS benefit with the pembrolizumab-containing regimen [21]. Importantly, the combination of chemotherapy with immunotherapy has been shown to further enhance PCR rates [22,23], These findings suggest that immunochemotherapy may represent a more effective and innovative therapeutic approach for LA-OSCC, with promising clinical potential. However, the above studies exclusively enrolled treatment-naive surgical candidates, excluding patients receiving definitive nonoperative management or those with recurrent disease. Consequently, the findings may not fully represent real-world treatment outcomes across broader patient populations.

Our study, which integrates data from three centers, included a broad population of patients with LA-OSCC undergoing neoadjuvant therapy. To our knowledge, this represents the first study in China reporting outcomes in over 150 patients treated with neoadjuvant chemoimmunotherapy for LA-OSCC. The data indicate that sequential therapy after neoadjuvant treatment is important; both surgery and radiotherapy following neoadjuvant therapy were associated with improved survival. We observed that the standard treatment regimen—neoadjuvant therapy followed by surgery plus adjuvant radiotherapy—was associated with the most favorable observed survival outcomes. Although our study did not incorporate radiotherapy combined with PD-1 inhibitors as a maintenance strategy post-surgery, we observed that patients achieving pCR had a favorable prognosis, suggesting that treatment intensity could potentially be individualized according to treatment response in the future. However, adjuvant immunotherapy maintenance or de-escalation was not evaluated in our study, and prospective randomized trials are warranted to validate response-adapted adjuvant strategies.

For patients with LA-OSCC, most surgeons would likely continue to base the extent of radical resection on the pretreatment tumor involvement [24]. The conventional radical resection often results in significant functional morbidity—including dysphagia, dysphonia, impaired mastication, and aspiration risk. Studies by Huang et al. and Li et al. [25,26] have demonstrated that neoadjuvant therapy with paclitaxel, cisplatin, and a PD-1 inhibitor improves survival in patients with OSCC. In our study, we implemented a response-adapted surgical protocol, in which tumor-specific resection margins were determined by post-neoadjuvant regression patterns, with rigorous intraoperative frozen-section confirmation of R0 resection. This strategy was designed to maintain oncologic safety while minimizing functional compromise. Notably, this approach was also associated with favorable oncologic outcomes in recurrent LA-OSCC. Histopathologic analyses revealed that even in tumors with substantial treatment response, residual malignant foci frequently persist in a dispersed, fragmented distribution within pretreatment anatomical boundaries [27]. This finding provides a mechanistic rationale for incorporating adjuvant radiotherapy into our treatment paradigm, which may have contributed to the favorable oncologic outcomes observed in this cohort.

Researchers suggests that oral submucosal fibrosis related to betel-quid exposure may impair local drug delivery [28]. We observed that primary tumor location was associated with imaging-based tumor volume reduction, with buccal tumors showing a trend toward attenuated response. However, OSF status was not recorded in this cohort, and the available data do not establish fibrosis as the cause of this observation. Alternative explanations include greater T4 burden and tobacco/alcohol exposure in the buccal subgroup, differences in tumor vascularity, hypoxia, stromal reaction, or immune microenvironment. The prior reports showing that patients achieving a major pathologic response (MPR) after neoadjuvant therapy have superior 3-year overall survival (OS) compared to those with >10% viable tumor [29]. Our findings support this association and suggest that pCR/CR may be robust predictors of 3-year OS. These results are consistent with existing evidence and may provide a clinically useful framework for stratifying therapeutic response in LA-OSCC. Furthermore, although patients achieving a pCR exhibited higher rates of tumor volume reduction compared to those with pPR, the volumetric response in pCR patients did not reach 100%. This underscores the need for further exploration of more effective predictive methods to identify patients who are likely to attain pCR .

Our study provides observational evidence that neoadjuvant sequential therapy was associated with improved outcomes in recurrent LA-OSCC. Historically, the management of this disease has predominantly involved non-surgical approaches. For instance, pembrolizumab in combination with carboplatin and paclitaxel has been established as the first-line regimen for recurrent OSCC, with reporting a 12-month median overall survival rate of 53.2%[30]. In our recurrent subgroup, patients who underwent surgery after neoadjuvant therapy had a 16-month overall survival rate of 63.4%, a time point approximating the median follow-up of this subgroup. Given the retrospective design and limited follow-up, these findings support continued exploration of the neoadjuvant-plus-surgery approach in recurrent OSCC. Notably, however, the pathologic response rate to neoadjuvant therapy in recurrent LA-OSCC was significantly lower than in treatment-naive patients. Given the higher operative risks and uncertain long-term benefits in this population, many patients may ultimately decline surgical resection despite initial eligibility. These observations underscore the need for rigorous patient selection based on tumor biology and treatment response, and comprehensive shared decision-making that incorporates individualized risk-benefit assessments. Cao et al. reported survival differences by anatomical subsite in patients undergoing de-escalated surgery after neoadjuvant chemoimmunotherapy. In our cohort, primary tumor site was not statistically significantly associated with overall survival, although a trend was observed. This discrepancy may reflect differences in eligibility criteria, stage and recurrence status, treatment response, subsequent surgery or radiotherapy selection, and the limited number of events within subsite strata.

The CPS has historically been utilized to predict the efficacy of anti-PD-1 monoclonal antibody therapy in patients with recurrent or metastatic HNSCC [31]. In another cohort of patients with HNSCC treated with anti-PD-1 monoclonal antibody plus apatinib, the CPS has similarly been identified as a key predictive marker for major pathological response [32]. Statistically, a CPS >20 represents a critical threshold for predicting major pathological response, whereas a CPS < 1 is considered incompatible with neoadjuvant immunotherapy [33]. Nevertheless, in our randomly selected cohort, the CPS did not predict the pathological response to neoadjuvant therapy among patients with LA-OSCC—In our exploratory analysis of 32 patients, no statistically significant association between CPS and pathological response was observed; however, the analysis was substantially underpowered, and no definitive conclusion regarding the predictive utility of CPS can be drawn. The observation that a considerable proportion of PCR patients exhibited low or undetectable PD-L1 expression raises the hypothesis that chemotherapy combined with PD-1 blockade may exert synergistic antitumor activity enabling complete pathological responses even in CPS-low tumors—a possibility that warrants validation in larger, prospectively enrolled cohorts.

Several limitations of this study should be acknowledged. First, as a retrospective analysis, treatment allocation was not randomized, and patients who proceeded to surgery were more likely to be responders with better performance status and fewer socioeconomic barriers to care. Although propensity score matching and inverse probability weighting yielded consistent estimates, not all covariates achieved the prespecified SMD <0.10 threshold after matching, and these methods adjust only for measured confounders. Therefore, residual imbalance and selection bias cannot be excluded; our findings establish association rather than causation. Second, the median follow-up of 1.35 years is relatively short for a disease in which late recurrences are common, and longer follow-up is needed to confirm the durability of the observed association. Third, the cohort was drawn from three centers in a single region, which may limit the generalizability of our findings to other populations. Fourth, several different PD-1 inhibitors were used, reflecting real-world practice but introducing treatment heterogeneity. Finally, the CPS substudy was exploratory and underpowered. Prospective randomized trials with standardized response assessment and longer follow-up are warranted to confirm these findings.

In this multicenter retrospective cohort, response-directed R0 resection and risk-adapted radiotherapy after neoadjuvant PD-1 inhibition plus paclitaxel–cisplatin were associated with improved overall survival in treatment-naive and recurrent LA-OSCC. Given the non-randomized design and potential residual confounding, these findings should be interpreted as associations and require confirmation in prospective randomized trials.

## Supplementary Material

Supplementary Figure S3.docx

Supplementary table1.docx

scanned copy of approved study protocol.pdf

Supplementary Figure S1.docx

Supplementary Figure S2.docx

Supplementary Figure S4.docx

Supplementary table2.docx

## Data Availability

Data, analytic methods, and study materials will be made available to other researchers upon request to the corresponding author.
