# Supplementary material for: R0 resection following neoadjuvant PD-1 inhibition with paclitaxel-cisplatin is associated with improved overall survival in locally advanced and recurrent oral squamous cell carcinoma: multicenter retrospective cohort study
Source: Ann Med. 2026 Oct 29;58(1):2744077. doi: 10.1080/07853890.2026.2744077 (PMC13721807; doi:10.1080/07853890.2026.2744077)
Supplement: Supplementary file 1 [file IANN_A_2744077_SM3829.pdf]

普特利单抗联合化疗新辅助治疗在初发、可切除、中线受侵的局部晚期舌鳞癌的探索性研究

研究方案

方案编号： HX008-OTSCC-II-01

方案版本： V2.0

方案版本日期： 2024 年 04 月 15 日

研究发起单位： 湖南省肿瘤医院

研究发起者： 田皞 教授

目录

试验方案摘要 3
1.研究背景 12
1.1 口腔鳞癌和头颈鳞癌治疗现状 12
1.2 新辅助化疗 12
1.3 新辅助免疫治疗 13
1.4 新辅助免疫联合化疗研究现状 15
1.5 研究药物普特利单抗介绍 17
2. 研究目标 21
3. 研究内容和意义 21
4. 研究设计 22
5、技术路线 24
6、研究方法 24
6.1 入组标准 24
6.2 试验过程的观察和评价 27
6.3 治疗方案 29
7、临床观察项目 38
8、患者生存获益评价 39
9、不良事件 40
9.1 定义： 40
9.2 不良事件记录 40
9.3 不良事件严重程度判定标准 40
9.4 不良事件与研究药物关系的判断标准 41
9.5 严重不良事件以及记录与报告 42
10、数据管理与统计分析 42
10.1 病例报告表的填写与移交 42
10.2 数据的录入与修改 42
10.3 数据锁定 43
10.4 统计分析 43
11、伦理学要求和受试者知情同意书 43
12、资料保存 44
附录 47
附录 1 肿瘤评价-新实体瘤缓解评价标准-版本 1.1 47
附录 2 ECOG 活动状态评分 55
附件 3 免疫相关不良事件管理参考办法 56
附件 4 体重指数（BMI）计算公式 62
附件 5 EORCT QLQ C30（生活质量评分）量表 63
附件 6 欧洲癌症研究组织(EORTC)头颈癌生存质量问卷(QLQ H&N35) 65
附件 7 华盛顿大学生存质量问卷第 4 版（UW-QOL，V4.0） 67

试验方案摘要

标题	普特利单抗联合化疗新辅助治疗在初发、可切除、中线受侵的局部晚期舌鳞癌的探索性研究
试验编号：	HX008-OTSCC-II-01
试验药品：	普特利单抗注射液（普佑恒）：重组人源化抗 PD-1 单克隆抗体注射液<br>顺铂，白蛋白紫杉醇
主要研究者	田皞
研究方法	开放、单中心、单臂、II 期研究
目标人群与研究创新性	拟选择经多学科会诊后局部晚期初治舌鳞癌（OTSCC）患者，以 III 期或 IVA 期浸及中线及过中线舌鳞癌患者为研究对象，予普特利单抗+紫杉类+铂类三药联合的新辅助治疗 3 周期后，评估该联合方案临床疗效及安全性，**以期在手术前达到缩瘤抑瘤、减小手术范围，以半舌或部分舌切除达到根治目的，减少手术创伤，尽最大可能保留舌功能，提升生活质量。**
研究目的	**主要研究目的：**<br>• 评估普特利单抗联合化疗新辅助治疗在初发、可切除、中线受侵的局部晚期舌鳞癌的疗效。<br>**次要研究目的：**<br>• 评估普特利单抗联合化疗新辅助治疗在初发、可切除、中线受侵的局部晚期舌鳞癌的生活质量评分、功能保留和安全性。
研究合理性	口腔鳞癌中，舌鳞癌发病率处于首位，对于舌鳞癌的治疗，目前多采取手术联合放、化疗或靶向联合放疗等综合模式进行治疗，但疗效仍不理想，2 年无进展生存率不到 40%，5 年总生存率在 20% -30%，35%-60%患者会出现局部区域复发。另外，对局晚舌鳞癌患者，尤其是浸及中线及过中线舌鳞癌患者，进行大范围的根治性手术可能给患者造成舌活动差，语言沟通，吞咽等功能障碍，影响患者外观导致社交困难，严重影响患者的生活质量。为进一步提高治疗疗效，延长患者生存，近年来，较多研究探索局部晚期舌鳞癌的新辅助治疗方案，包括免疫或靶向药物联合化疗等方向，目前仍无标准方案出现，本研究旨在探索局晚舌鳞癌特别是高肿瘤负荷的患者中有效安全的新辅助治疗方案。免疫治疗（ICIs）能让部分患者长期生存，耐受性良好，适合于局晚期舌鳞癌

	的新辅助治疗。因此，开展探索新型治疗方案的临床试验 ,以提高对局部区域极晚期口腔鳞癌患者的治疗效果具有重要意义。<br>普特利单抗（Pucotenlimab）是一款我国自主研发的针对人 PD-1 的人源化 IgG4 单抗，可高亲和力与 PD-1 结合，以通过阻断 PD-1 与其配体 PD-L1 及 PD-L2 的结合来恢复免疫细胞杀死癌细胞的能力。普特利单抗注射液采用创新分子设计以延长其半衰期，显示出强大的临床抗肿瘤活性以及良好的安全性。普特利单抗抗体设计上在铰链区引入了 S228P 突变来阻止 IgG4 的 Fab 臂交换，同样使得其抗体的稳定性极大大增加；其创新性地采用抗体工程技术，于 Fc 区引入三重突变，提高 FcRn 的结合亲和力，从而大幅延长其半衰期，显示出优异的临床疗效及患者的药物依从性，且三重突变修饰并未增加其 ADCC 和 CDC 效应。普特利单抗注射液的半衰期均值为 21.8（单次给药）及 38.2 天（稳态）。此外普特利单抗差异化识别 PD-1 的关键表位 N58，结合后可使得免疫细胞的 PD-1 表达下调，以及降低 PD-1 与肿瘤细胞 PD-L1 的结合能力，进而发挥信号阻断作用，临床前头对头动物模型研究比较证实，普特利单抗比 K 药和 O 药有更强的抗肿瘤效果。普特利单抗于 2022 年 7 月，经国家药品监督管理局（NMPA）批准，用于不可切除或转移性的高度微卫星不稳定型（MSI-H）或错配修复缺陷型（dMMR）的晚期实体瘤，同年 9 月获批第二个适应症，为既往接受全身系统治疗失败的不可切除或转移性黑色素瘤。
研究药物和给药方式	研究药物：<br>试验药品：普特利单抗注射液（普佑恒）<br>生产厂家：乐普生物科技股份有限公司<br>剂型：注射液<br>规格：100mg，采用 10mL 西林瓶包装<br>用法：静脉滴注<br>储存条件：密封、避光，储存时需置于 2-8℃医用冰箱。<br>给药方式：<br>本研究是一项探索性临床研究，符合入选、排除标准的舌鳞癌患者在 21 天周期的第 1 天通过静脉 (IV) 输注接受 200 mg 普特利单抗作为手术前的新辅助治疗，持续 3 个周期，术后按指南标准治疗。
研究终点	主要研究终点:<br>• 3 年总生存（OS）率。

	次要研究终点:<br>•主要病理缓解率（MPR）：定义为手术切除的原发灶和区域淋巴结内，存活肿瘤细胞比例≤10%。<br>•病理完全缓解（Pathologic Complete response，pCR）率<br>•客观缓解率（ORR）<br>•无进展生存期(Progression-free survival, PFS)<br>•总生存期(Overall Survival, OS)<br>•无事件生存时间(EFS)：定义为从入组开始到以下任何事件的首次记录日期的时间：疾病进展；根据指示通过影像学或活检评估的局部或远处复发；或因任何原因死亡<br>•安全性：按照 NCI-CTCAE v5.0，对治疗中出现的不良事件（TEAE）和治疗相关不良反应（TRAE）进行评估。<br>•生活质量评分：使用的评估量表包括欧洲癌症研究与治疗组织生活质量核心量表（EORTC QLQ-C30）及其头颈部肿瘤子量表（EORTC QLQ-H&N35），以评估症状和功能相对基线的变化。<br>•功能保留：使用的评估量表为华盛顿大学生存质量问卷第 4 版（UW QOL，V4.0），同时收集术后是否需要预防性气管切开，是评估舌鳞癌患者功能保留和治疗改良的重要评价指标。
参加中心	单中心：湖南省肿瘤医院
入组病例	总病例数 27 例

<table>
<tr>
<td>样本量统计假设</td>
<td>
参考背景文献报道数据，以及科室临床实践中新辅助化疗的 ORR，假设新辅助化疗的历史 ORR 为 30%，可作为基线。假设新辅助免疫+化疗的 ORR 为 57%，用 PASS15 软件计算样本量为 27 例（假设 10%的脱落率）。

参考文献：S. Nakahara, Y. Takenaka, K. Ogawa, et al. Phase II study of docetaxel, cisplatin, and concurrent radiation followed by platinum-based adjuvant chemotherapy for technically unresectable, locally advanced head and neck squamous cell carcinoma. Int J Clin Oncol[J]. 2016;21(6):1030-1037.

Semrau, F. Waldfahrer, M. Lell, et al. Feasibility, toxicity, and efficacy of short induction chemotherapy of docetaxel plus cisplatin or carboplatin (TP) followed by concurrent chemoradio-therapy for organ preservation in advanced cancer of the hypopharynx, larynx, and base of tongue. Early results. Strahlenther Onkol[J]. 2011;187(1):15-22

Huang Y, Sun J, Li J, et al. Neoadjuvant immunochemotherapy for locally advanced resectable oral squamous cell carcinoma: a prospective single-arm trial (Illuminate Trial) [published online ahead of print, 2023 Jun 6]. Int J Surg. 2023;10.1097/JS9.0000000000000489. doi:10.1097/JS9.0000000000000489.

A phase 2 study of neoadjuvant chemotherapy plus durvalumab in resectable locally advanced head and neck squamous cell carcinoma/https://pubmed.ncbi.nlm.nih.gov/37395170/

PASS 15.0.5　　2023/11/14 13:28:03
Tests for One Proportion
Numeric Results for Testing One Proportion using the Z-Test with S(P0)
Alternative Hypothesis: Two-Sided (H0: P = P0 vs. H1: P ≠ P0)
<table>
<tr><th>Power*</th><th>n</th><th>Proportion Given H0 P0</th><th>Proportion Given H1 P1</th><th>Difference P1-P0</th><th>Alpha</th><th>Reject H0 If |Z| ></th></tr>
<tr><td>0.80444</td><td>24</td><td>0.3000</td><td>0.5700</td><td>0.2700</td><td>0.0500</td><td>1.9600</td></tr>
</table>
* Power was computed using the normal approximation method.
</td>
</tr>
<tr>
<td>统计分析及方法</td>
<td>•一般资料分析</td>
</tr>
</table>

	评价年龄、性别、分期等一般资料。<br>•不良反应分析<br>采用 NCI-CTC5.0 标准评价患者毒副反应、并发症等。<br>•疗效分析<br>1.有效性评价：根据 RECISTv1.1 评估标准分为 CR、PR、SD、PD。<br>2.生存获益评价：MPR、pCR、ORR、无疾病进展生存期（Progression-free Survival，PFS）和总生存期（Overall survival，OS）；。
入组标准	（1）自愿参加临床研究并签署知情同意书。<br>（2）年龄≥18 岁，且≤70 岁。<br>（3）经病理确诊的原发性舌鳞癌：III 期或 IVA 期浸及中线及过中线舌鳞癌。<br>（4）未接受过针对目前头颈部肿瘤的放疗、化疗、免疫治疗或生物治疗等抗肿瘤治疗。<br>（5）ECOG PS 评分：0-2 分，对 ECOG 的评估应在治疗开始之日前 7 天内进行。<br>（6）原发灶可手术切除，且患者愿意接受手术治疗。<br>（7）至少有一个可测量病灶（根据 RECIST v1.1）。<br>（9）主要器官功能充分，定义为：<br>血常规（在检查前 14 天内，不得接受输血、白蛋白、重组人促血小板生成素或集落刺激因子治疗）：白细胞（WBC）≥4.0×109/L，绝对中性粒细胞计数（ANC）≥1.5×109/L，血小板计数（PLT）≥100×109/L，血红蛋白（Hb）≥90g/L（14 天内未输血及血制品，未使用造血刺激因子纠正）；<br>肝功能：总胆红素（TBIL）≤2.5×ULN，丙氨酸氨基转移酶（ALT）和天门冬氨酸氨基转移酶（AST）≤3×ULN，血清白蛋白≥ 3.0g/dL（30 g/L）；<br>肾功能：肌酐清除率（Ccr）≥60ml/min（Cockcroft-Gault 公式）；<br>凝血功能：国际标准化比值（INR）或凝血酶原时间（PT）≤1.5×ULN。<br>（10）经研究者评估，心、肺功能正常。<br>（11）血压正常，伴有高血压病患者血压控制良好（不高于 150/90 mmHg）。<br>（12）无明显化疗和免疫治疗的禁忌症。

	（13）自愿在研究治疗期间及治疗结束后 120 天内采取有效避孕措施。<br>（14）育龄女性必须已经采取可靠的避孕措施，在入组前 7 天内进行血妊娠试验，且结果为阴性。
排除标准	出现以下任一标准的受试者不可纳入本研究：<br>（1）原发灶不明确。<br>（2）已知有远处转移。<br>（3）既往接受过抗 PD-1/PD-L1 抗体、抗 PD-L2 抗体、CTLA-4 抗体，或其他作用于 T 细胞共刺激或检查点通路的药物/抗体。<br>（4）存在严重活动性自身免疫疾病，首次给药前 2 年内需要接受糖皮质激素或系统性免疫抑制治疗患者。疾病已受控制且不需要系统性免疫抑制治疗患者允许入组，如 I 型糖尿病、只需要接受激素替代治疗的甲状腺功能减退症和无需行全身治疗的皮肤疾病（例如，白癜风、银屑病和脱发）。<br>（5）患有先天或后天免疫功能缺陷（如 HIV 感染）、活动性乙型肝炎（HBV-DNA≥1×104 拷贝数/ml 或 2000 IU/ml）或丙丙型肝炎（丙肝抗体阳性且 HCV-RNA 高于分析方法的检测下限）。<br>（6）首次给药前 14 天内，存在感染性疾病，需要使用全身性抗感染治疗。<br>（7）研究药物首次用药前 14 天内或研究期间，需要使用皮质类固醇

	(>10 mg/天泼尼松或等效剂量的同类药物)或其他免疫抑制剂进行系统治疗的受试者。在没有活动性自身免疫疾病的情况下，允许吸入或局部使用类固醇，或剂量 ≤10 mg/天泼尼松疗效剂量的肾上腺激素替代。<br>（8）首次给药前 14 天内，使用过有抗肿瘤适应症的中草药或中药制剂（以说明书为准）。<br>（9）已知有特定物质滥用（如精神类药物、酗酒等）或吸毒史。<br>（10）首次给药前 6 个月内，出现心脑血管疾病（心肌梗死、严重/不稳定型心绞痛、 NYHA 2 级以上心功能不全及症状性充血性心力衰竭、脑血管意外如卒中等）或控制不良的心律失常（包括 QTc 间期＞480ms，QTc 间期以 Fridericia 公式计算）。<br>（11）经研究者评估，接受过重大手术且首次给药前 28 天内仍未恢复。<br>（12）首次给药前 6 个月内，具有重大血管疾病或出血性疾病病史（例如，需要手术修补或近期有外周动脉血栓形成的主动脉瘤、门静脉高压伴随消化道出血等）。<br>（13）进入研究前 3 年内曾诊断为任何其他恶性肿瘤，可进行局部治疗且已治愈的皮肤基底细胞癌或鳞状细胞癌、浅表性膀胱癌、宫颈原位癌、乳腺导管内原位癌和甲状腺乳头状癌除外。<br>（14）既往或准备进行器官移植（包括骨髓移植）。<br>（15）首次使用研究药物前 4 周内接种过活疫苗（允许接种灭活疫苗）。<br>（16）入组前 4 周内，参加过其他临床试验。<br>（17）已知对研究药物或其任何辅料过敏，或者对其他单克隆抗体发生过严重过敏反应。<br>（18）妊娠期或哺乳期妇女。<br>（19）存在其他严重身体或精神疾病或实验室检查异常，可能增加参与研究的风险，或影响研究依从性，或干扰研究结果以及研究者认为不适合参与本研究的患者。
评价时间	在 3 程新辅助化疗联合免疫后进行复查，按 RECISTv1.1 疗效评价标准评价，记录为 CR、PR、SD 或 PD。根据随访结果计算各组患者的 3 年总生存率、无进展生存率、无局部区域复发生存率及无远处转移生存率。<br>从受试者入组后开始普特利单抗联合化疗治疗，3 个疗程结束后进行手术，术后应根据研究医生的判断决定后续的巩固治疗方案。
研究持续时间	从第一例患者入组到最后一例患者完成至少 24 周的随访（数据截止日

	期与首次给药日期间隔≥24 周）后进行数据分析，共需要约 3 年时间。
安全性	（1）基线体征和症状<br>研究者将在筛选期间对受试者进行全面体格检查，记录受试者的生命体征和相关症状及有临床意义的异常结果。<br>（2）体格检查和生命体征<br>研究者将按研究计划的流程，于每次随访时对受试者进行体格检查。<br>（3）实验室安全性评估<br>研究者将按研究计划的流程，进行血常规、生化常规、凝血功能、尿常规等项目分析。
药代动力学	一项 1 期研究评估了 30 例中国晚期恶性实体瘤患者使用 4 个不同剂量普特利单抗（1mg/kg 、3 mg / kg 、10 mg / kg 、200 mg /次，每 3 周一次给药）的药代动力学。结果显示第 1 周期给予本品后，在 1-10 mg / kg 的剂量范围内体内暴露（ Cmax 、 AUC0-1 。）随剂量增加而增加，Cmax 与 AUC0-1 增加比例与剂量增加比例大致相当。每 3 周给药一次，给药 6 次后，基本达到稳态浓度。
分析计划	1 一般资料分析<br>评价年龄、性别、分期等一般资料。<br>2 不良反应分析<br>采用 NCI-CTC5.0 标准评价患者毒副反应、并发症等。<br>3 疗效分析<br>本研究的主要研究终点为根据 RECIST（1.1 版）评估的 ORR。主要终点的分析在最后一例受试者完成至少 24 周的随访（数据截止日期与首次给药日期间隔≥24 周）后进行。<br>ORR 将基于 FAS 进行。用 ClopperPearson 法估计其 95%精确双侧置信区间（CI）。采用 Kaplan-Meier 法分析到达事件终点（DoR、PFS 和 OS）的时间。图形分析将包括靶病灶肿瘤负荷百分比随时间自基线变化的蜘蛛图、靶病灶肿瘤负荷自基线变化最佳百分比的瀑布图。<br>4 分层分析<br>按 PD-L1 表达等进行分层分析
试验结束时间	按照试验方案，已达到了收集计划的全部数据的目的，最后一例受试者完成给药和随访。
计划试验日期	2024 年 3 月-2024 年 6 月

	召开启动会议构建数据平台及课题组成员系统学习及培训制定试验方案并送伦理委员会批准<br>2024 年 6 月-2025 年 6 月<br>临床试验入组及执行试验方案<br>2025 年 6 月-2026 年 6 月<br>临床资料随访，撰写文章（临床试验方案的毒副反应、有效率、顺应性及生存质量，疗效评价，在国际 SCI 杂志发表结果）<br>2026 年 6 月-2027 年 6 月<br>临床资料随访，国际 SCI 杂志发表相关文章（远期疗效评价及晚期副反应）

1.研究背景

1.1 口腔鳞癌和头颈鳞癌治疗现状

口腔鳞状细胞癌（oral squamous cell carcinoma，OSCC）是口腔颌面部最常见的恶性肿瘤，尤其湖南是槟榔消费大省,槟榔相关口腔癌的发病率明显高于全国平均水平，是严重威胁我省人民身体健康主要癌种之一。除舌尖腹面粘膜下少数腺体聚集外，其他舌体粘膜下无腺体，因此舌癌95％以上是鳞状细胞癌。根治性手术联合术后放疗或放化疗的综合治疗是局部晚期口腔鳞癌的主要治疗手段，但其效果并不理想，局部晚期患者的5年生存率仅30%左右，而部分局部极晚期患者(T4或者N3期)甚至无手术机会，仅能予以姑息性放化疗。近年来，有研究者尝试增加术前TPF方案诱导化疗(多西他塞-顺伯-5-氟尿嘧啶)来提高局部晚期口腔鳞癌患者的疗效，但研究结果表明TPF方案诱导化疗对5年总体生存率没有明显改善作用。因此，开展探索新型治疗方案的临床试验 ,以提高对局部区域极晚期口腔鳞癌患者的治疗效果具有重要意义。

口腔鳞癌中，舌鳞癌（oral tongue squamous cell carcinoma，OTSCC）发病率处于首位，对于舌鳞癌的治疗，目前多采取手术联合放、化疗或靶向联合放疗等综合模式进行治疗，但疗效仍不理想，2年无进展生存率不到40%，5年总生存率在20% -30%，35%-60%患者会出现局部区域复发。另外，对局晚舌鳞癌患者，尤其是浸及中线及过中线舌鳞癌患者，进行大范围的根治性手术可能给患者造成舌活动差，语言沟通，吞咽等功能障碍，影响患者外观导致社交困难，严重影响患者的生活质量。为进一步提高治疗疗效，延长患者生存，近年来，较多研究探索局部晚期舌鳞癌的新辅助治疗方案，包括免疫或靶向药物联合化疗等方向，目前仍无标准方案出现，本研究旨在探索局晚舌鳞癌特别是高肿瘤负荷的患者中有效安全的新辅助治疗方案。

1.2 新辅助化疗

既往多项研究证实，TPF方案（紫杉类+铂类+5-氟尿嘧啶）在局晚HNSCC中新辅助/诱导治疗观察到较好的功能保留率、无进展生存期、OS等，奠定了TPF新辅助/诱导化疗的地位，但TPF显示出较高毒性III/IV度中性粒细胞数减少发生率更高，且TPF新辅助/诱导治疗方案或对后续CRT造成负面影响。有研究进一步探索了高效低毒的替代治疗方案。

尽管在一些研究中远处失败率有所改善，但在最终放疗前接受新辅助多西他赛、顺铂和氟尿嘧啶的患者中未显示出生存优势。因此，新辅助化疗仅获得NCCN指南类别2B推荐水平，并未广泛纳入局部晚期HNSCC以及舌鳞癌等为主的口腔鳞癌的治疗模式。

在2022年欧洲肿瘤内科学会（ESMO）年会壁报交流中，上海交通大学第九人民医院钟来平教授团队展示了增加西妥昔单抗、多西他赛和顺铂的新辅助化疗对可手术的口腔/口咽部鳞状细胞癌（OOPSCC）的治疗效果。本研究旨在评价使用多西他赛、顺铂和西妥昔单抗（TPE）进行新辅助化疗靶向治疗后，再进行手术和术后放疗（S+PORT）与直接S+PORT治疗在局部晚期可切除的OOPSCC患者中的疗效。TPE组总体缓解率（ORR）和pCR率分别为43.4%（59/136）和10.5%（14/133）。本研究未能证明增加新辅助药物TPE治疗可以提高局部晚期可切除OOPSCC患者的生存，而pCR患者可从新辅助药物TPE治疗中生存获益。

2021年12月6日，J Clin Oncol杂志在线发表一项2期试验结果，目的是探索在可手术的口腔癌中，新辅助化疗在缩小肿瘤和下颌骨保留中的潜在作用和安全性。患者1:1随机分配到先手术（节段性切除），随后接受辅助治疗（标准治疗组）；或两个周期新辅助化疗（多西他赛、顺铂和氟尿嘧啶），随后接受手术和放化疗（干预组）。标准治疗组的患者根据最终组织病理学报告进行治疗。主要终点是下颌骨保留率。次要终点是局部控制率、无病生存期、总生存期和治疗相关毒性。干预组中29例患者接受了手术，1例患者在新辅助化疗获得完全缓解后接受同步放化疗，15例患者接受边缘下颌骨切除术。 34例患者中，共16例患者获得下颌骨保留，下颌骨保留率为47%（95% CI，31.49~63.24；15例边缘下颌骨切除术和1例同步放化疗）。长期随访结果显示，两组患者复发的数量几乎相同，远处转移患者的数量也没有差异。

1.3 新辅助免疫治疗

免疫检查点抑制剂治疗在目前肿瘤治疗研究领域中备受关注。免疫检查点抑制剂通过结合免疫检查点，解除免疫系统对肿瘤细胞的耐受，提高T细胞对肿瘤细胞的有效识别和杀伤，重启肿瘤免疫循环。目前，肿瘤免疫检查点靶点如细胞毒T淋巴细胞抗原4（cytotoxicTlymphocyte-associatedantigen-4,CTLA-4）和程序性死亡受体1/配体1（programmedcelldeathreceptor1/ligand1,PD-1/PD-L1）已经显示出明显临床疗效。PD-1在活化T细胞表面表达，在生理条件下，与配体PD-L1、PD-L2结合抑制T细胞增殖和活化，导致机体免疫抑制。然而，肿瘤细胞大量表达PD-L1，与PD-1识别、结合，减弱机体抗肿瘤免疫力使肿瘤存活[20-22]。

PD-1抑制剂的作用机制是针对PD-1设计特定的蛋白质抗体，阻止PD-1和PD-L1的识别过程，部分恢复T细胞功能，从而使T细胞可以杀死肿瘤细胞[23]。既往临床研究报道PD-L1过表达与肿瘤的预后不良相关，阻断PD-1信号会改善恶性肿瘤患者的预后[22]。肿瘤患者的常规治疗（手术、化疗和放疗）联合PD-1抑制剂免疫治疗是安全的，对增强机体的免疫功

能，控制肿瘤的进展，抑制肿瘤的复发和转移、延长生存期具有较好的作用。PD-1单抗辅助或新辅助免疫治疗联合放化疗或手术是目前肿瘤研究的热点。

免疫治疗是近年来新兴的肿瘤治疗方法，PD-1/PD-L1通路研究较成熟，帕博利珠单抗，纳武利尤单抗已获批用于复发转移头颈鳞癌的治疗。目前免疫治疗在头颈领域的探索逐渐从复发转移阶段向局部晚期过渡。

小鼠模型的研究表明，新辅助免疫治疗和手术优于手术和辅助免疫治疗，而在手术前进行新辅助免疫治疗的时机对于确定最大疗效非常重要。在口腔癌的小鼠模型中，新辅助抗-PD-1免疫检查点抑制剂治疗引起了T细胞的应答，而辅助治疗则没有。针对PD-1/PD-L1通路的新辅助免疫治疗在结直肠癌和肺癌。综上所述，这些临床前研究表明，在局部晚期HNSCC的治疗中，新辅助免疫检查点抑制剂治疗可能发挥潜在作用。

免疫检查点抑制剂(ICI)是治疗复发/转移性 HNSCC 的标准推荐方案。多个临床前数据表明，在同期和新辅助治疗情况下，加用 ICI 是安全的，不会延迟手术或减少放化疗的完成。在同时进行的情况下，尽管 ICI 的使用在所有已发表的试验中都显示出短期反应，但还没有发表 PFS 或 OS 获益的数据。在新辅助治疗中，与 ICI 单药治疗相比，联合 ICI 治疗的主要病理反应率优于单药治疗，但不良事件发生率没有显著增加，但其在提高生存率方面的价值尚不清楚。ICI 疗效似乎受到肿瘤特征的影响，特别是 PD-L1联合阳性评分、HPV 状态和肿瘤微环境。在未经治疗的 LA HNSCC 中，ICI 的使用具有显著的异质性，同时使用多种明确的新辅助治疗方案。

Checkmate 358[16]（头颈队列）是第一个头颈部肿瘤PD-1抑制剂新辅助治疗的1/2期临床研究， 52名III-IV期HNSCC患者在第1天和第15天接受了nivolumab治疗，并在第29天进行了手术。23新辅助nivolumab耐受性良好，并在23.5%的HPV阳性HNSCCs和5.9%的HPV阴性肿瘤中诱导病理消退，这表明人乳头瘤病毒阴性恶性肿瘤需要更积极的治疗方案,如何进一步提高局部晚期头颈鳞癌患者综合治疗疗效意义重大。

另一项临床研究IMCISION（NCT03003637）招募了32名口腔部位T2期或更高期的未治疗HNSCC患者。患者分为两组，一组在第1周和第3周接受NIVO单抗治疗，另一组在第1周接受NIVO单抗和伊匹木单抗的联合治疗。有3名患者登记后被发现不符合资格，共29例患者（6例Nivolumab单药，23例联合治疗）进行最后的分析。第二次剂量给药后3至7天，患者接受手术治疗。8名采用联合治疗患者（35%，95%CI:16-57%）和1名采用Nivolumab单药治疗患者（17%，95%CI:0-64%）实现了主要病理缓解（MPR）。1名采用联合治疗患者实现病理学完全缓解（pCR）。23名联合治疗患者中的2名（9%，95%CI:1-28%）实现部分病理

缓解（PPR）。在4名Nivolumab单药治疗患者和18名联合治疗患者中观察到免疫相关的不良事件（irAEs）。其中，两名Nivolumab单药治疗患者和10名联合治疗患者发展为3-4级irAEs。此外，没有观察到未知或其他的irAEs。

Uppaluri等人在研究手术可切除的局部晚期HNSCC中，调查新辅助和辅助性的pembrolizumab时发现类似的病理学反应率。共招募了36名患者，他们接受了单剂量的新辅助pembrolizumab治疗，随后在2-3周后进行手术切除。有阳性切缘和/或淋巴结外扩的患者继续接受辅助性pembrolizumab治疗。22%的患者达到PTR1，另外还有22%达到PTR2。接受辅助性pembrolizumab治疗的高风险病理学患者有16.7%的1年复发率，与类似患者的历史数据相比表现良好。有趣的是，PTR0患者表现出抑制性检查点的上调，而PTR1和PTR2与干扰素γ（IFN-g）活性、基线PD-L1表达和免疫细胞浸润相关，这提出了选择适合新辅助免疫治疗的患者的生物标志物的可能性。作者试图描绘肿瘤细胞克隆损失的模式，但未能得出明确的与PTR和肿瘤细胞克隆动力学相关的结论。（病理性肿瘤反应(PTR)根据包含角质碎片、巨细胞和坏死的肿瘤床的比例来定义，并使用以下分级方案来评估:PTR0，无反应或反应少于10%；PTR1，大于等于10%且小于50%；和PTR2，50%或更高的反应。）

一项大型国际III期试验(NCT03765918)，KEYNOTE-689，目前正在进行进行中。该试验旨在招募600名先前未经治疗、高风险、可切除的局部晚期HNSCC患者。患者被随机分配到两组:(1)新辅助帕博利珠单抗和手术，随后是标准护理辅助治疗加辅助帕博利珠单抗或(2)手术加标准护理辅助治疗。主要终点是根据RECIST 1.1的MPR和无事件生存率，次要终点包括总生存率、临床部分缓解率和生活质量结果指标。

1.4 新辅助免疫联合化疗研究现状

在其他恶性肿瘤的新辅助治疗中，化疗和免疫治疗的结合已经得到证实。在新辅助卡铂和紫杉醇化疗中加入帕博利珠单抗增加了II期和III期乳腺癌患者临床部分缓解的百分比。此外，有几项正在进行的可切除非小细胞肺癌研究将抗PD-1、抗PD-L1抗体与化疗相结合，中期数据显示了良好的病理反应率。不出所料，这种组合策略开始在局部晚期HNSCC进行评估。

2023年6月，上海交通大学医学院附属第九人民医院钟来平教授课题组成果发表于International journal of surgery（影响因子15.300），探索了PD-1单抗联合化疗的新辅助治疗用于局晚可切除口腔鳞状细胞癌的疗效、安全性和相关生物标志物分析。本研究是一项关于NAICT用于LAOSCC疗效的前瞻性临床研究。研究纳入标准包括：年龄区间18-75岁；病理诊断为口腔鳞状细胞癌；临床分期为III-IV期；ECOG PS分级为0-1；器官和骨髓功能良好等。

排除标准包括先前的全身性癌症治疗、自身免疫性相关疾病或任何可能干扰临床试验入组的情况。治疗方案：应用特瑞普利单抗（PD-1抑制剂）联合白蛋白紫杉醇/顺铂，21天为一个周期，总共治疗两个周期。每周期第1天通过静脉滴注依次应用白蛋白紫杉醇（260 mg/m2）、顺铂（75 mg/m2）和特瑞普利单抗（240 mg），随后实施根治性手术和辅助治疗。主要研究终点是安全性和主要病理缓解（MPR）率。通过靶向的下一代测序（NGS）和多重荧光免疫组化（MIF）技术，对NAICT前后的肿瘤样本进行分子特征和肿瘤免疫微环境的评估。该研究的临床试验注册号为NCT04473716。NAICT和R0切除完成率均为100%。20例受试者均纳入NAICT疗效的病理学和影像学评估。根据术后病理评估，MPR率为60%（12/20）（95% CI：36.41%－80.02%），其中pCR率为30%（6/20）（95% CI：12.84%－54.33%）。根据RECIST v1.1标准，2例患者（10%）达到完全缓解（CR），10例患者（50%）达到部分缓解（PR）；客观缓解率（ORR）为60%（12/20）（95% CI：36.41%－80.02%）。

一项2期单臂多中心研究（DOI: 10.1002/cncr.34930）评估了可切除局部晚期 HNSCC 患者使用卡铂，白蛋白紫杉醇和 durvalumab 的新辅助化疗免疫治疗。主要终点是假设病理完全缓解率为50%。化学免疫治疗和手术切除后，患者接受研究确定的病理风险适应辅助化疗，包括单独的 durvalumab (低风险) ，涉及野外放疗加每周顺铂和 durvalumab (中等风险) ，或标准放化疗加 durvalumab (高风险)。39名受试者中共有35名接受了计划中的手术切除; 一名受试者由于治疗相关的毒性而延迟了手术。最常见的治疗相关不良事件是血细胞减少、疲劳和恶心。治疗后影像学检查显示客观有效率为57% 。病理完全缓解率和主要病理缓解率分别为29% 和49% 。1年无进展生存率为83.8% (95% 置信区间，67.4%-92.4%)。

对可切除HNSCC患者进行新辅助NIVO单抗联合每周卡铂和紫杉醇的评估。在新辅助方案中，给予三次Nivolumab。在26名患者中，69%获得了MPR或临床部分反应。另一项正在进行的研究是评估在西妥昔单抗的新辅助铂类治疗中加入西米普利单抗，特别是减少手术切除范围的能力，以及临床-病理降级，例如可以省略术后放疗(有或没有化疗)。有理由预计，在新辅助治疗中，免疫治疗与化疗的结合可能允许外科医生减少肿瘤切除的范围，修改基于辅助放疗的治疗，将不可切除的局部晚期疾病转化为可切除的疾病，同时潜在地产生抗肿瘤记忆T细胞，这可能潜在地减少局部和远处复发的可能性。**上述多个研究提示我们，在头颈肿瘤特别是舌鳞癌中，新辅助抗PD-1单抗联合化疗可能是一个有效的方案，为患者提供了新的治疗选择。**

免疫疗法（ICIs）已经作为治疗头颈部鳞状细胞癌（HNSCC）复发和/或转移病情的主流治疗方式进入了临床。这些研究中鼓舞人心的数据迅速推动了将这些药物作为治愈目标方案的一部分的发展。研究人员正在评估ICIs作为术前新辅助治疗方案的组成部分（单一药

物和联合免疫疗法、化疗和免疫疗法的组合），并有证据显示出肿瘤显著的治疗反应，包括完全病理缓解。此外，人们对于在高风险疾病复发的患者中作为辅助治疗使用ICIs的潜在用途非常感兴趣。然而，目前还没有证据表明这种方法能够转化为改善无进展生存、无事件生存、无疾病复发以及总体生存等结果，希望随机研究的结果尽快公布。

总的来说，免疫疗法在HNSCC中的突破值得期待，如何选择治疗的适用人群、适用时间、治疗周期、适用策略和适宜的疗效评价方法依然是未来的工作重点。现开展本**探索性研究，新辅助普特利单抗+紫杉类+铂类初发局部区域舌鳞癌的安全性和有效性。探寻最佳用药时机、用药周期、个性化治疗方案以及降低毒副作用发生率，并利用新辅助治疗前后临床样本开展新辅助治疗分子标靶的探寻，为初发局晚期口腔鳞癌的诊疗提供坚实的询证医学证据。**

1.5 研究药物普特利单抗介绍

普特利单抗（Pucotenlimab）是一款我国自主研发的针对人 PD-1 的人源化 IgG4 单抗，可高亲和力与 PD-1 结合，以通过阻断 PD-1 与其配体 PD-L1 及 PD-L2 的结合来恢复免疫细胞杀死癌细胞的能力。普特利单抗注射液采用创新分子设计以延长其半衰期，显示出强大的临床抗肿瘤活性以及良好的安全性。普特利单抗抗体设计上在铰链区引入了 S228P 突变来阻止 IgG4 的 Fab 臂交换，同样使得其抗体的稳定性极大大增加；其创新性地采用抗体工程技术，于 Fc 区引入三重突变，提高 FcRn 的结合亲和力，从而大幅延长其半衰期，显示出优异的临床疗效及患者的药物依从性，且三重突变修饰并未增加其 ADCC 和 CDC 效应。普特利单抗注射液的半衰期均值为 21.8（单次给药）及 38.2 天（稳态）。此外普特利单抗差异化识别 PD-1 的关键表位 N58，结合后可使得免疫细胞的 PD-1 表达下调，以及降低 PD-1 与肿瘤细胞 PD-L1 的结合能力，进而发挥信号阻断作用，临床前头对头动物模型研究比较证实，普特利单抗比 K 药和 O 药有更强的抗肿瘤效果[1]。

普特利单抗于 2022 年 7 月，经国家药品监督管理局（NMPA）批准，用于不可切除或转移性的高度微卫星不稳定型（MSI-H）或错配修复缺陷型（dMMR）的晚期实体瘤，入组 100 例受试者，普特利单抗 200mg，静脉滴注，三周给药一次，中位随访 22.5 个月，独立评审委员会评估结果，整体 ORR 高达 49%（95% CI　38.86%－59.20%），DCR 为 70.0%（95% CI 60.02%～78.76%；CRC 人群 ORR 高达 56.3%，DCR 为 70.4%（95% CI 58.41, 80.67）；FAS 全分析集的 mPFS 和 mOS 仍未达到。由研究者评估的 ORR 为 50.0%（95% CI 39.83%-60.17%），DCR 为 73.0%（95% CI 63.20%-81.39%）；中位 PFS 为 22.8（6.18,NR）个月［2］。

同年 9 月获批第二个适应症，为既往接受全身系统治疗失败的不可切除或转移性黑色素瘤；该研究共计 119 例受试者，普特利单抗 3mg/kg，静脉滴注，三周给药一次，中位随访时

间为 19.32 个月，整体人群的 ORR 高达 20.2%，为目前获批同类产品最高，其中皮肤型和原发病灶不明型黑色素瘤的 ORR 分别高达 36.4%和 33.3%；普特利单抗治疗使患者的长期生存也得到显著延长，整体人群的 mOS 长达 17.2m，其中肢端型黑色素瘤的 mOS 长达 20.1m，而皮肤型患者 mOS 仍未达到［3］；目前正在开展普特利单抗联合经肝动脉化疗栓塞术（TACE）一线治疗 IV 期 M1c 黑色素瘤肝转移的随机、开放、多中心 III 期临床研究(HX008-III-MM-01)。

普特利单抗有多项实体瘤治疗领域的探索研究，包括联合卡陪他滨及奥沙利铂一线治疗晚期胃癌的单臂、多中心、Ib 期研究，共入组 35 例晚期胃癌患者，截止 2021 年 5 月，中位随访时间为 22.0 个月。ORR 高达 60.0%（CPS≥1 人群 ORR 高达 75.0%），mPFS 最优，为 9.2 个月，mDOR 为 12.3 个月，mOS 长达 15.3m［4］。

联合伊利替康二线治疗晚期胃癌的单臂、多中心、II 期研究，入组了 58 例晚期胃癌患者，截止 2021 年 2 月，中位随访时间为 18.8 个月。ORR 高达 27.6%（CPS≥1 人群 ORR 高达 38.5%），mPFS 最为 4.2 个月，mDOR 为 12.8 个月，mOS 长达 12.1 个月（CPS≥1 人群 mOS 长达 17.7 个月）［5］。

普特利单抗联合顺铂和吉西他滨一线治疗 TNBC 的单臂、多中心、Ib 期研究，入组 31 例患者，截至 2021 年 8 月 4 日，中位随访 18.1 个月，ORR 高达 80.7%，1 例 CR，24 例 PR，DCR 达 100%，mPFS 9.0 个月［6］，显著优于化疗。

普特利单抗联合贝伐珠单抗或仑伐替尼一线治疗晚期肝癌的研究获得喜人结果，在普特利单抗联合贝伐珠单抗队列，入组 28 例晚期肝癌患者，接受 HX008（200 mg IV Q3W）联合贝伐珠单抗（15 mg/kg IV Q3W）的治疗方案，中位随访时间为 15.2 个月，ORR 高达 28.6%，DCR 为 75.0%；mPFS 长达 7.5 个月，mOS 尚未达到；普特利单抗联合仑伐替尼队列，入组 47 例晚期肝癌患者，接受 HX008（200mg IV Q3W）联合仑伐替尼（体重≥60 kg 为 12mg/d；<60 kg 为 8mg/d）的治疗方案，中位随访时间为 12.5 个月，ORR 为 36.2%，DCR 为 80.9%，mPFS 为 9.6 个月［7］。

此外，在这项开放标签 II 期研究中，纳入组织学诊断为 IIIB 或 IV 期的首次接受全身治疗的 nsq-NSCLC 患者。患者静脉滴注普特利单抗 200mg，Q3W，最长使用 35 个周期，联合铂+培美曲塞 Q3W，4 个周期，后续培美曲塞，Q3W，维持治疗。主要终点是总缓解率(ORR)和安全性，次要终点是无进展生存期(PFS)、反应持续时间(DOR)和总生存期(OS)。从 2020 年 9 月 25 日至 2021 年 4 月 15 日，共入组 43 例患者，截止日期(2022 年 8 月 3 日)，中位随访时间为 13.1 个月。22 例患者获得部分缓解，20 例保持病情稳定，ORR 为 48.8% (95%CI: 38.9%， 59.2%)， DCR 为 95.3% (95%CI: 88.7%， 98.4%)。中位 PFS 和 DOR 分别为 11.1 个月(95%CI: 6.8, 14.4)和 13.9 个月(95%CI: 6.6, NE)。中位 OS 未达到［8］。

尽管目前暂无普特利单抗在头颈部肿瘤的研究数据，但在上述各项研究中，普特利单抗均取得良好的临床疗效，且患者安全性和耐受性均良好，比如在普特利单抗单药治疗

MSI-H/dMMR 实体瘤研究中，≥3 级 TRAEs 发生率是 18%；在普特利单抗单药治疗黑色素瘤研究中，≥3 级 TRAEs 发生率是 15.1%，均未发生治疗相关死亡事件，不同研究发生率不同，公司没有发布统一的数据，各项研究的具体发生率可参见发表文献。普特利单抗是我国自主研发的人源化抗 PD-1 单克隆抗体，其独特的结合位点 N58，具有更强的与 pdL1 的结合力，Fc 段三重突变修饰，显著延长抗体半衰期，具有抑瘤作用强，安全性好的特点，为免疫治疗提供新的选择。

参考文献：

[1]Cancer Genome Atlas Network. Comprehensive genomic characterization of head and neck squamous cell carcinomas. Nature[J]. 2015;517(7536):576-582.
[2]J. Ferlay, H. R. Shin, F. Bray, et al. Estimates of worldwide burden of cancer in 2008: GLOBOCAN 2008. Int J Cancer[J]. 2010;127(12):2893-2917.
[3]W. E. Samlowski, J. Moon, J. P. Kuebler, et al. Evaluation of the combination of docetaxel/carboplatin in patients with metastatic or recurrent squamous cell carcinoma of the head and neck (SCCHN): a Southwest Oncology Group Phase II study. Cancer Invest[J]. 2007;25(3):182-188.
[4]Y. He, T. Guo, J. Wang, et al. Which induction chemotherapy regimen followed by cisplatin-based concurrent chemoradiotherapy is the best choice among PF, TP and TPF for locoregionally advanced nasopharyngeal carcinoma? Ann Transl Med[J]. 2019;7(5):104.
[5]H. Peng, L. L. Tang, B. B. Chen, et al. Optimizing the induction chemotherapy regimen for patients with locoregionally advanced nasopharyngeal Carcinoma: A big-data intelligence platform-based analysis. Oral Oncol[J]. 2018;79:40-46.
[6]S. Nakahara, Y. Takenaka, K. Ogawa, et al. Phase II study of docetaxel, cisplatin, and concurrent radiation followed by platinum-based adjuvant chemotherapy for technically unresectable, locally advanced head and neck squamous cell carcinoma. Int J Clin Oncol[J]. 2016;21(6):1030-1037.
[7]S. Semrau, F. Waldfahrer, M. Lell, et al. Feasibility, toxicity, and efficacy of short induction chemotherapy of docetaxel plus cisplatin or carboplatin (TP) followed by concurrent chemoradio-therapy for organ preservation in advanced cancer of the hypopharynx, larynx, and base of tongue. Early results. Strahlenther Onkol[J]. 2011;187(1):15-22.
[8] Lenouvel, Daniel. Implications of PD-L1 Expression in Oral Squamous Cell Carcinomas. Granada: Universidad de Granada, 2021. [http://hdl.handle.net/10481/66662].
[9]J. B. Liao, R. E. Swensen, K. J. Ovenell, et al. Phase II trial of albumin-bound paclitaxel and granulocyte macrophage colony-stimulating factor as an immune modulator in recurrent platinum resistant ovarian cancer. Gynecol Oncol[J]. 2017;144(3):480-485.
[10]M. G. Fury, E. J. Sherman, S. S. Rao, et al. Phase I study of weekly nab-paclitaxel + weekly cetuximab + intensity-modulated radiation therapy (IMRT) in patients with stage III-IVB head and neck squamous cell carcinoma (HNSCC). Ann Oncol[J]. 2014;25(3):689-694.
[11]J. Weiss, J. Gilbert, A. M. Deal, et al. Induction chemotherapy with carboplatin, nab-paclitaxel and cetuximab for at least N2b nodal status or surgically unresectable squamous cell carcinoma of the head and neck. Oral Oncol[J]. 2018;84:46-51.
[12]D. Adkins, J. Ley, K. Trinkaus, et al. A phase 2 trial of induction nab-paclitaxel and cetuximab given with cisplatin and 5-fluorouracil followed by concurrent cisplatin and radiation for locally advanced squamous cell carcinoma of the head and neck. Cancer[J]. 2013;119(4):766-773.
[13]A. D. Santin, W. Deng, M. Frumovitz, et al. Phase II evaluation of nivolumab in the treatment of persistent or recurrent cervical cancer (NCT02257528/NRG-GY002). Gynecol Oncol[J]. 2020;157(1):161-166.
[14]Y. Song, Q. Gao, H. Zhang, et al. Treatment of relapsed or refractory classical Hodgkin lymphoma with the anti-PD-1, tislelizumab: results of a phase 2, single-arm, multicenter study. Leukemia[J]. 2020;34(2):533-542.
[15] Jifang Gong Lin Shen,　Yuqin Song, et al. GLS-010 Zimberelimab), a Novel Fully Human anti-PD-1 mAb in fatients with Advanced Tumors Preliminary Results of a Phase lb Clinical

Trial. cancer research[J]. 2020;80 (Supplement 16): 3275.
[16]Gonçalves A Ferris R, Baxi S, et al. . An open-label, multicohort, phase 1/2 study in patients with virus-associated cancers (CheckMate 358): Safety and efficacy of neoadjuvant nivolumab in squamous cell carcinoma of the head and neck (SCCHN). . Presented at ESMO 2017 Congress; September 8-12, 2017; Madrid, Spain Abstract LBA46[J]. 2017.
[17] Jibin Zhang,etc.HX008: a humanized PD-1 blocking antibody with potent antitumor activity and superior pharmacologic properties.MAbs. 2020 Jan-Dec;12(1):1724751. doi: 10.1080/19420862.2020.1724751.
[18] Bo Zhang, et al.Pucotenlimab in patients with advanced mismatch repair-deficient or microsatellite instability-high solid tumors: A multicenter phase 2 study.Cell Rep Med. 2023 Dec 19;4(12):101301. doi: 10.1016/j.xcrm.2023.101301.

[19] Chuanliang Cui, et al.Safety and efficacy of Pucotenlimab (HX008) - a humanized immunoglobulin G4 monoclonal antibody in patients with locally advanced or metastatic melanoma: a single-arm, multicenter, phase II study.BMC Cancer. 2023 Feb 6;23(1):121. doi: 10.1186/s12885-022-10473-y.
[20]Jianming Xu, et al.Anti-PD-1 antibody HX008 combined with oxaliplatin plus capecitabine for advanced gastric or esophagogastric junction cancer: a multicenter, single-arm, open-label, phase Ib trial.Oncoimmunology. 2020 Dec 31;10(1):1864908.doi: 10.1080/2162402X.2020.1864908.
[21] Yan Song et al.HX008, an anti-PD1 antibody, plus irinotecan as second-line treatment for advanced gastric or gastroesophageal junction cancer: a multicenter, single-arm phase II trial.J Immunother Cancer. 2020 Oct;8(2):e001279. doi: 10.1136/jitc-2020-001279.
[22]Jun Cao et al.Phase 1b clinical trial of pucotenlimab (HX008), a novel anti-PD-1 monoclonal antibody, combined with gemcitabine and cisplatin in the first-line treatment of metastatic triple-negative breast cancer.Front Oncol. 2022 Aug 2;12:837963. doi: 10.3389/fonc.2022.837963. eCollection 2022.
[23]Hong Zhao, et al.Anti-PD1 antibody pucotenlimab in combination with bevacizumab or lenvatinib in a phase II study for the treatment of advanced hepatocellular carcinoma (aHCC).J Clin Oncol 41, 2023 (suppl 16; abstr e16180),DOI ： 10.1200/JCO.2023.41.16_suppl.e16180.
[24]Caicun Zhou et al. Pucotenlimab (HX008) - humanized anti-PD-1 monoclonal antibody plus pemetrexed and platinum as first-line treatment for advanced non-squamous non-small-cell lung cancer (nsqNSCLC): A single-arm multicenter phase II study.Annals of Oncology (2023) 34 (suppl_2): S755-S851. 10.1016/annonc/annonc1331

2. 研究目标

（1）主要研究目的：评价免疫检测点抑制剂普特利单抗+紫杉类+铂类初发局部区域舌鳞癌（III期或IVA期浸及中线及过中线）的3年总生存（OS）率。

（2）次要研究目的：

2.1探索普特利单抗+紫杉类+铂类初发局部区域舌鳞癌（III期或IVA期浸及中线及过中线）的近期疗效(客观缓解率ORR，完全缓解率CR，部分患者率PR），通过缩瘤抑瘤作用，减小手术范围，以半舌或部分舌切除达到根治目的同时保留患者的器官提高生活质量。同时评价远期疗效（2年无进展生存率、2年无复发生存率和2年无远处转移生存率)。

2.2手术切除率以及手术后R0切除率，完成影像学及术后病理标本肿瘤患者率比对以及新 辅助治疗前后原发灶、淋巴结等有效缓解率的比对。

2.3免疫检查点抑制剂PD-1、白蛋白紫杉醇、顺伯新辅助治疗局部区域极晚期口腔鳞癌的生存质量评分、功能保留（UW-QOL量表和术后是否需要预防性气管切开）和安全性（不良事件发生率等）。

（3）探索性研究内容：

3.1普特利单抗+紫杉类+铂类初发局部区域舌鳞癌（浸及中线及过中线）新辅助治疗前后患者肿瘤组织、淋巴结及外周血中免疫细胞组成及其功能和表达谱的变化趋势及其与疗效之间的相关性。

3.2普特利单抗+紫杉类+铂类初发局部区域舌鳞癌（浸及中线及过中线）新辅助治疗后临床缓解与病理缓解的相关性，以及与远期疗效的相关性。

3.3 PD-L1表达情况与疗效的相关性。

3.4 新辅助治疗前后肿瘤原发灶真实生发中心比对及研究。

（3）研究终点指标：3 年总生存（OS）率为主要研究终点，病理完全缓解（Pathologic Complete response，pCR）率、主要病理缓解率（MPR）、客观缓解率 ORR、无进展生存期(Progression-free survival, PFS)、总生存期(Overall Survival, OS)、生存质量评分、功能保留和安全性为次要研究终点。

3. 研究内容和意义

本研究是一项单中心、单臂、前瞻性的探索性临床研究，评价普特利单抗联合化疗作为可切除初发局部晚期舌鳞癌新辅助治疗的疗效和安全性。拟选择经多学科会诊后局部晚期初治舌鳞癌患者，以浸及中线及过中线舌鳞癌患者为研究对象，予普特利单抗+紫杉类+铂类三药联合的新辅助治疗3周期后，评估该联合方案临床疗效及安全性，以期在手术前达到缩

瘤抑瘤、减小手术范围，以半舌或部分舌切除达到根治目的，减少手术创伤，尽最大可能保留舌功能，提升生活质量。并将入组患者与既往新辅助治疗前湖南省肿瘤医院局晚期舌鳞癌手术患者进行组间比较，以验证实验目的，为该类患者高效低毒的新辅助治疗策略。

为提高舌鳞癌总体人群中的远期生存率，推动学科的发展，以患者生存获益为中心，以现有的肿瘤治疗进展与早期临床结果为导向，现开展本探索性研究，这也和未来肿瘤诊疗精准化人群分层和全程精细化管理这一思想相契合。需要强调，转化治疗只是治疗全程中的一个起始环节，局晚期舌鳞癌的转化治疗方案选择、治疗反应评估、手术时机判断以及术后辅助治疗等，应依托于多学科诊疗团队共同决策。现阶段，转化治疗在临床路径、治疗方式选择、手术时机、手术指征等关键问题上仍待统一和规范化。多项以减少复发、延长生存为目标的术前转化和术后辅助研究也正持续进行。期待转化治疗及手术后患者管理模式的进一步完善，为提高舌鳞癌患者的长期生存助力。

本研究意义：客观反应率越高的联合治疗方案越具有对局晚期舌鳞癌成功转化的潜力，越值得积极尝试。转化治疗后外科手术的必要性主要包括：①完整肿瘤的病理检查可以明确转化治疗效果，并指导后续的辅助治疗；②适时外科干预可在一定程度上少ICIs和化疗的治疗用药，减少耐药性及药物不良反应；③半舌切除手术是达到根治性目的的重要保证，达到CR或PR并长期维持更是确保远期生存获益的前提。对局晚期舌鳞癌患者而言，保持功能和生活质量同样要考虑，疗效固然是方案制订时要考虑的一方面，药物的安全性也同样重要。有了良好的安全性为前提，患者才能保证良好的依从性，才有机会从治疗中获益。无论在治疗模式、理念，还是手段、方案方面，本研究的治疗方案都是规范、合理、有效的，既充分发挥了免疫治疗前置的优势，也充分利用了多模式综合治疗的技术优势。

本研究的创新点：以初发局晚期舌鳞癌（III 期或 IVA 期浸及中线及过中线）为研究对象，术前利用普特利单抗+紫杉类+铂类进行三周期新辅助治疗，通过影像学评估并结合专科检测等评判其近期疗效，评价舌癌退缩至半舌以内比例(影像学评估舌中隔以内 1cm 以上)，将缩瘤达到半舌以内的患者进行缩瘤手术，手术方案以半舌切除以内为标准，将半舌，同侧口底以及口底肌群整体贯通切除，术中快速切片验证舌正中份切缘安全性，术后与本中心既往单纯手术治疗的同类别舌鳞癌患者进行组间比较，判断近期疗效与远期疗效，评价生活质量。保留部分器官和功能是本研究的亮点，假如患者的生存获益与既往单纯化疗相比差不多，但是生活质量大大提高，也是优势。更何况患者的ORR,PFS,OS都还是有可能超过既往单纯化疗的数据。

4. 研究设计

4.1 研究样本量以及病例分配

参考背景文献报道数据，以及科室临床实践中新辅助化疗的 ORR，假设新辅助化疗的历史 ORR 为 30%，可作为基线。新辅助免疫+化疗的 ORR 为 57%，用 PASS15 软件计算样本量为 27 例（假设 10%的脱落率）。

参考文献：S. Nakahara, Y. Takenaka, K. Ogawa, et al. Phase II study of docetaxel, cisplatin, and concurrent radiation followed by platinum-based adjuvant chemotherapy for technically unresectable, locally advanced head and neck squamous cell carcinoma. Int J Clin Oncol[J]. 2016;21(6):1030-1037.

Semrau, F. Waldfahrer, M. Lell, et al. Feasibility, toxicity, and efficacy of short induction chemotherapy of docetaxel plus cisplatin or carboplatin (TP) followed by concurrent chemoradio-therapy for organ preservation in advanced cancer of the hypopharynx, larynx, and base of tongue. Early results. Strahlenther Onkol[J]. 2011;187(1):15-22

Huang Y, Sun J, Li J, et al. Neoadjuvant immunochemotherapy for locally advanced resectable oral squamous cell carcinoma: a prospective single-arm trial (Illuminate Trial) [published online ahead of print, 2023 Jun 6]. Int JSurg. 2023;10.1097/JS9.0000000000000489. doi:10.1097/JS9.0000000000000489.

A phase 2 study of neoadjuvant chemotherapy plus durvalumab in resectable locally advanced head and neck squamous cell carcinoma/https://pubmed.ncbi.nlm.nih.gov/37395170/

PASS 15.0.5　　　　　　　　　　　　　　　　　　　　　2023/11/14 13:28:03

Tests for One Proportion

Numeric Results for Testing One Proportion using the Z-Test with S(P0)
Alternative Hypothesis: Two-Sided　(H0: P = P0 vs. H1: P ≠ P0)

Power*	n	Proportion Given H0 P0	Proportion Given H1 P1	Difference P1-P0	Alpha	Reject H0 If |Z| >
0.80444	24	0.3000	0.5700	0.2700	0.0500	1.9600

* Power was computed using the normal approximation method.

4.2 试验对象的随机方法及盲法

按就诊顺序登记所有符合入组条件的舌鳞癌患者，每个患者将肿瘤分期等临床信息输入系统后入组。

本研究采取的是非盲法的单臂临床研究。

4.3 试验对象分组

不适用。

4.4 剂量选择依据

普特利单抗I期研究从1mg/kg到10mg/kg以及200mg的剂量爬坡过程中，各剂量组在首次给药后21天内，均没有发生DLT，表明普特利单抗注射液的MTD超过了10mg/kg，虽然没有爬到MDT，但已经有了明显的疗效趋势，同时也是为了确保患者安全性的前提下，就没有再继续爬坡剂量；而通过I期研究证实，普特利单抗注射液在3 mg/kg剂量下的稳态半衰期约24天，与帕博利珠单抗相似；200mg固定剂量下连续6周给药稳态下的半衰期约为38天；根据临床前和I期临床的PK特征，以及参考帕博利珠单抗的II期剂量，推荐普特利单抗3mg/kg剂量和200mg固定剂量，每三周（21天）给药一次（Q3W）开展的II期临床研究。Q3W也是目前长效ICIs比较常用的给药周期，便于与其他Q3W的药物配伍使用，为进一步提高患者治疗依从性、降低未来患者治疗经济成本，本研究采用200mg Q3W剂量开展后续试验。

5、技术路线

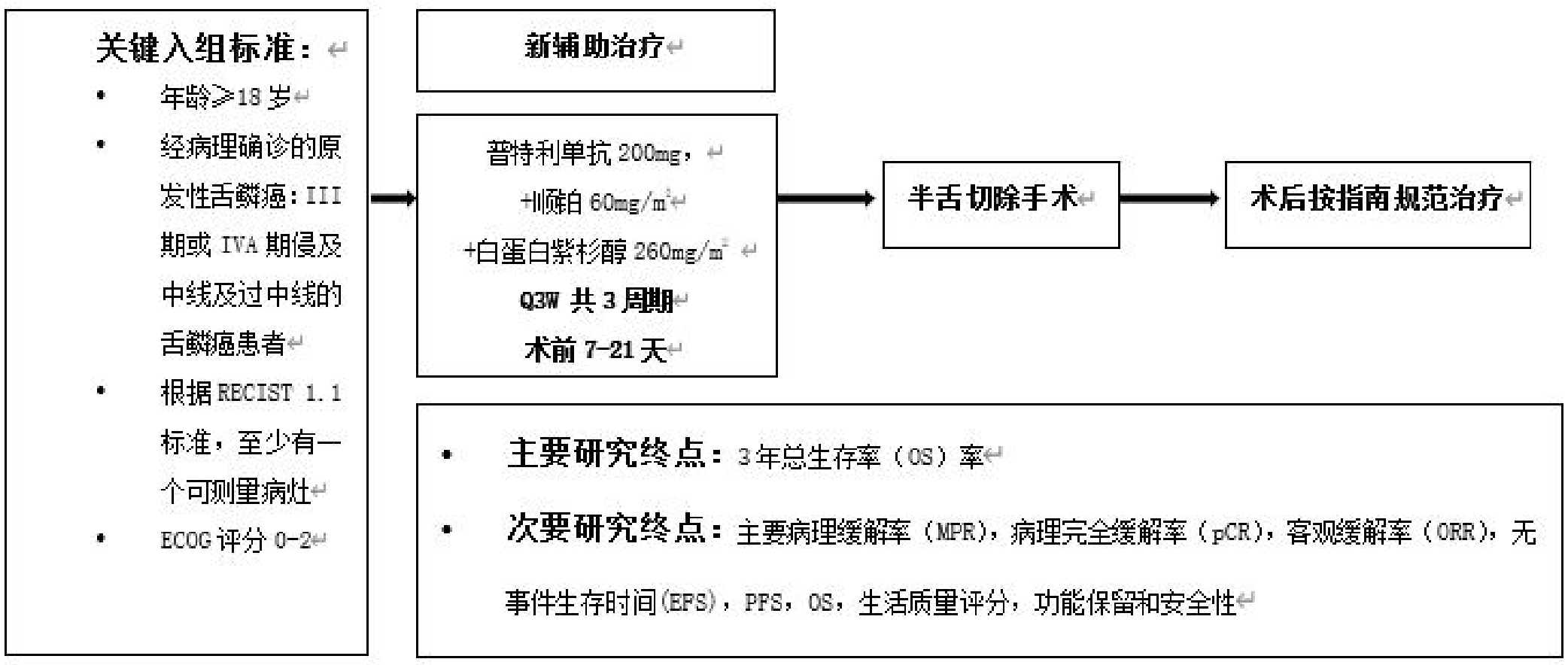

6、研究方法

本研究为单中心、开放、II期、探索性临床试验。

6.1 入组标准

（1）入选标准

1. 自愿参加临床研究并签署知情同意书。
2. 年龄≥18 岁，且≤70 岁。
3. 经病理确诊的原发性舌鳞癌：III 期或 IVA 期浸及中线及过中线舌鳞癌。
4. 未接受过针对目前头颈部肿瘤的放疗、化疗、免疫治疗或生物治疗等抗肿瘤治疗。
5. ECOG 评分 0-2 分。
6. 原发灶可手术切除，且患者愿意接受手术治疗。

7. 至少有一个可测量病灶（根据 RECIST v1.1）。
8. 主要器官功能充分，定义为：

   血常规（在检查前 14 天内，不得接受输血、白蛋白、重组人促血小板生成素或集落刺激因子治疗）：白细胞（WBC）≥4.0×10^9/L，绝对中性粒细胞计数（ANC）≥1.5×10^9/L，血小板计数（PLT）≥100×10^9/L，血红蛋白（Hb）≥90g/L（14 天内未输血及血制品，未使用造血刺激因子纠正）；

   肝功能：总胆红素（TBIL）≤2.5×ULN，丙氨酸氨基转移酶（ALT）和天门冬氨酸氨基转移酶（AST）≤3×ULN，血清白蛋白≥ 3.0g/dL（30 g/L）；

   肾功能：肌酐清除率（Ccr）≥60ml/min（Cockcroft-Gault 公式）；

   凝血功能：国际标准化比值（INR）或凝血酶原时间（PT）≤1.5×ULN。
9. 经研究者评估，心、肺功能正常。
10. 血压正常，伴有高血压病患者血压控制良好（不高于 150/90 mmHg）。
11. 无明显化疗和免疫治疗的禁忌症。
12. 自愿在研究治疗期间及治疗结束后 120 天内采取有效避孕措施。
13. 育龄女性必须已经采取可靠的避孕措施，在入组前 7 天内进行血妊娠试验，且结果为阴性。

（2）排除标准

出现以下任一标准的受试者不可纳入本研究：

- 原发灶不明确；
- 已知有远处转移。
- 既往接受过抗 PD-1/PD-L1 抗体、抗 PD-L2 抗体、CTLA-4 抗体，或其他作用于 T 细胞共刺激或检查点通路的药物/抗体。
- 存在严重活动性自身免疫疾病，首次给药前 2 年内需要接受糖皮质激素或系统性免疫抑制治疗患者。疾病已受控制且不需要系统性免疫抑制治疗患者允许入组，如 I 型糖尿病、只需要接受激素替代治疗的甲状腺功能减退症和无需行全身治疗的皮肤疾病（例如，白癜风、银屑病和脱发）。
- 患有先天或后天免疫功能缺陷（如 HIV 感染）、活动性乙型肝炎（HBV-DNA≥1×104 拷贝数/ml 或 2000 IU/ml）或丙丙型肝炎（丙肝抗体阳性且 HCV-RNA 高于分析方法的检测下限）。
- 首次给药前 14 天内，存在感染性疾病，需要使用全身性抗感染治疗。
- 研究药物首次用药前 14 天内或研究期间，需要使用皮质类固醇（>10 mg/天泼尼松或等效剂量的同类药物）或其他免疫抑制剂进行系统治疗的受试者。在没有活动性自身免疫疾病的情况下，允许吸入或局部使用类固醇，或剂量 ≤ 10 mg/天泼尼松

疗效剂量的肾上腺激素替代。

- 首次给药前 14 天内，使用过有抗肿瘤适应症的中草药或中药制剂（以说明书为准）。
- 已知有特定物质滥用（如精神类药物、酗酒等）或吸毒史。
- 首次给药前 6 个月内，出现心脑血管疾病（心肌梗死、严重/不稳定型心绞痛、NYHA 2 级以上心功能不全及症状性充血性心力衰竭、脑血管意外如卒中等）或控制不良的心律失常（包括 QTc 间期＞480ms，QTc 间期以 Fridericia 公式计算）。
- 经研究者评估，接受过重大手术且首次给药前 28 天内仍未恢复。
- 首次给药前 6 个月内，具有重大血管疾病或出血性疾病病史（例如，需要手术修补或近期有外周动脉血栓形成的主动脉瘤、门静脉高压伴随消化道出血等）；
- 进入研究前 3 年内曾诊断为任何其他恶性肿瘤，可进行局部治疗且已治愈的皮肤基底细胞癌或鳞状细胞癌、浅表性膀胱癌、宫颈原位癌、乳腺导管内原位癌和甲状腺乳头状癌除外。
- 既往或准备进行器官移植（包括骨髓移植）。
- 首次使用研究药物前 4 周内接种过活疫苗（允许接种灭活疫苗）。
- 入组前 4 周内，参加过其他临床试验。
- 已知对研究药物或其任何辅料过敏，或者对其他单克隆抗体发生过严重过敏反应。
- 妊娠期或哺乳期妇女。
- 存在其他严重身体或精神疾病或实验室检查异常，可能增加参与研究的风险，或影响研究依从性，或干扰研究结果以及研究者认为不适合参与本研究的患者。

（3）剔除标准

- 试验期未按规定用药及未能完成计划的患者（同时期化疗少于 2 周期；IMRT 放疗剂量低于试验方案中总剂量的 95%，放疗中断时间长于 10 天）。
- 因不良反应未按规定完成试验者不进行疗效分析（纳入不良反应统计）。
- 试验期间合用了其他药物影响疗效和毒性评价治疗者。
- 其他有关违反试验方案的患者。

（4）退出标准

- 研究过程中，若受试者出现严重不良事件或不可耐受的毒副反应，经研究者批准可停止研究，退出研究（纳入安全性分析）。
- 研究过程中，若半数以上的受试者出现不可耐受的毒副反应，或连续出现严重不良事件，研究者可提出提前终止试验。
- 受试者撤回知情同意，要求退出。

（5） 伴随药物及治疗

伴随治疗包括研究药物给药前7天至治疗结束访视期间，患者使用的任何药物（例如，处方药、非处方药、草药、营养补充剂）。应向研究者报告所有合并药物使用情况，并记录在CRF的合并用药中。

研究期间禁止使用以下药物：

- 任何其它研究性药物。
- 任何抗癌治疗、基于抗体的治疗、类视色素治疗、激素治疗、用亚硝基脲治疗、丝裂霉素 C、小分子酪氨酸激酶抑制剂、具有抗肿瘤活性的中成药，不允许对于靶病灶的姑息性放疗。
- 免疫抑制药物包括但不限于全身性糖皮质激素，剂量超过每天 10 mg 泼尼松或等效药物、甲氨蝶呤、硫唑嘌呤和 TNF-α 拮抗剂。使用免疫抑制药物治疗研究药物相关 AE 或在对比剂过敏的受试者中使用免疫抑制药物是可接受的。此外，允许使用吸入、局部和鼻内用糖皮质激素。允许糖皮质激素作为超敏反应的预防用药（如 CT 检查前用药）。
- 活疫苗（在研究期间至末次给药后的 120 天之内）。
- 不鼓励但也不禁止同时使用膳食补充剂、非研究者开具的药物和替代/补充治疗。
- 在首次输注普特利单抗之前，不允许使用预防输注相关反应的预先用药（即一级预防）；在发生输注相关反应后，允许在普特利单抗后续输注中使用任何计划的预防用药（即二级预防）。

6.2 试验过程的观察和评价

（1）试验前评价与筛选

按口腔舌鳞癌单病种规范管理，每位受试病人在进入试验之前必须完成下列项目，并明确病理及临床分期：

- 入选资格确定并签署知情同意书；
- 既往病史、治疗史、药物史；
- 身高、体重，体格检查：包括口腔检查及颈淋巴结在内的头颈部检查以及神经系统检查；
- 生命体征；
- ECOG 评分；
- 肿瘤病灶的影像学检查（口腔+颈部增强 MRI）
- 排除转移的检查：胸部/腹部/盆腔部 CT(平扫+增强)+骨 ECT，或 PET/CT 检查
- 心电图；
- 超声心动图；
- 乙型、丙型肝炎、HIV；
- 血液生化（肝功能、肾功能、电解质等）；

- 血常规（白细胞、中性粒细胞、血小板、血红蛋白）；
- 尿常规检查；
- 甲状腺功能检查（TSH、游离 T3、游离 T4）；
- 妊娠测试；
- 凝血功能。

（2）治疗期评价

从治疗开始到结束应评价以下内容：

- 治疗前、治疗结束时进行肿瘤病灶的 MRI 评估，采用 RECIST 疗效评价标准，评价指标为 CR、PR、SD 或 PD，结束时应复查胸片及腹部 B 超；
- 伴随药物的使用；
- 一般状况；
- 急性毒性反应（NCI-CTC 5.0 标准），包括血液学毒性、胃肠道反应、肝肾功能损害、粘膜炎、外周神经病变等；
- 实验室检查：诱导化疗阶段，每疗程 D1 进行心电图、血常规、生化常规、尿常规、HBsAg 定量检测（针对基线期 HBsAg 异常者）检查，D8、D15 检查血常规、生化常规、尿常规。试验组同期治疗和辅助治疗时每疗程 D1 进行心电图、血常规、生化常规、尿常规、甲状腺功能、凝血功能及 HBsAg 定量检测（针对基线期 HBsAg 异常者）检查，在每疗程用药后 D8、D15 检查血常规、生化常规、尿常规。标准治疗对照组同期治疗时每疗程 D1 进行心电图、血常规、生化常规、尿常规及 HBsAg 定量检测（针对基线期 HBsAg 异常者）检查，在每疗程用药后 D8、D15 检查血常规、生化常规。

（3）治疗结束评价

- ECOG 评分
- 血常规、生化常规
- 腹部 B 超或腹部 CT
- 胸部正侧位片或胸部 CT
- 全身骨扫描（需要时做）
- 记录不良反应

（4）随诊

放疗后第一年每2～3月随访一次，第二、三年每4～6月随访一次，以后每6个月随访一次。随诊方式采用记录患者返院复查资料，或由主管医生签名的信访、电话随访等，以评价患者的疗效及毒性反应的恢复情况，近期及远期毒性反应的情况。随访内容按单病种诊疗规范进行。

随访内容：常规头颈部检查，每3个月检查一次；B超、胸片每6个月复查一次；放疗结束后3个月必须复查颈部MRI，以后每半年或一年复查一次MRI。

由于随访过程中许多难以预计的情况，如患者不配合、人口流动、联系电话的更改等因素影响，导致失访率增加。为保证随访记录完整准确及降低失访率，需准确记录患者联系方式，增加多种途径的联系内容，包括主管医生及家属联系方式等，保证能从多种途径随访。

6.3 治疗方案

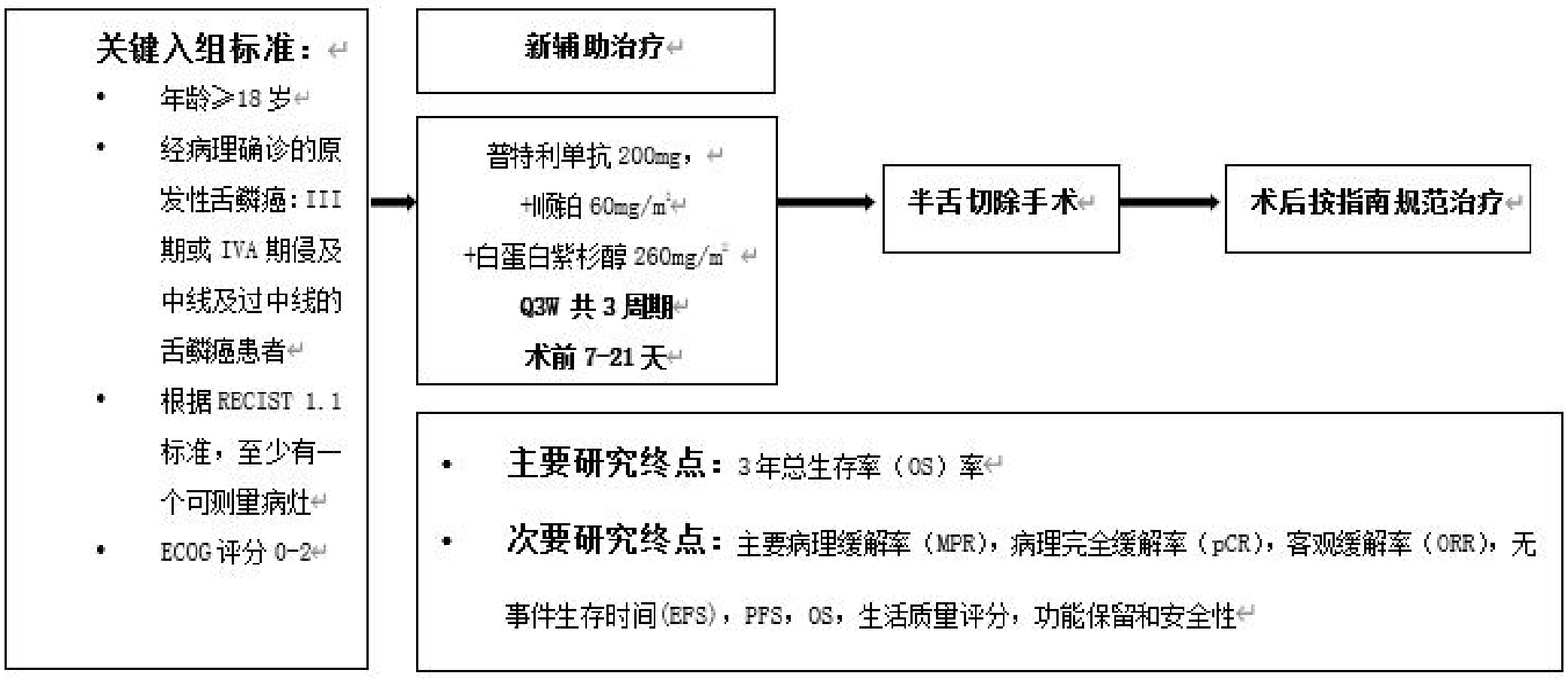

（1）化疗方案及剂量、疗程

招募局部晚期初治舌鳞癌患者，以浸及中线及过中线舌鳞癌患者为研究对象，予普特利单抗+紫杉类+铂类三药联合的新辅助治疗 3 周期后，评估该联合方案临床疗效及安全性，以期在手术前达到缩瘤抑瘤、减小手术范围，以半舌或部分舌切除达到根治目的，减少手术创伤，尽最大可能保留舌功能，提升生活质量，并将入组患者与既往新辅助治疗前湖南省肿瘤医院局晚期舌鳞癌手术患者进行组间比较，以验证实验目的，为该类患者高效低毒的新辅助治疗策略。

具体剂量如下，普特利单抗 200mg，从新辅助治疗的第一天开始，每 3 周静脉注射一次，共 3 个疗程。诱导化疗：白蛋白紫杉醇(每周期 260 mg/m^2，d1)和顺铂(每周期 60 mg/m^2，d1) ，每 3 周一次，共 3 个疗程。

- **化疗剂量调整**

药物	起始剂量	减少一个剂量级	减少两个剂量级
白蛋白紫杉醇	260mg/m^2	起始剂量的 80%	起始剂量的 60%
顺铂	60mg/m^2	起始剂量的 80%	起始剂量的 60%

a) 血液学毒性方面：

中性粒细胞		血小板	顺铂、白蛋白紫杉醇剂量调整
≥ 1.0×10⁹/L	和	≥ 75×10⁹/L	足量
0.5-<1.0×10⁹/L	和/或	50-< 75×10⁹/L	减量 1 个级别
< 0.5×10⁹/L<br>或粒细胞缺乏性发热<br>或粒细胞缺乏性感染	和/或	25-< 50×10⁹/L	减量 2 个级别

b)　肾功能毒性方面

①　停用 Cisplatin 至肌酐清除率≥ 60 ml/min。

②　若肌酐清除率<40 ml/min 则终止化疗。

③根据肌酐清除率进行的顺铂剂量调整：

肌酐清除率	顺铂
≥ 60ml / min	足量
40-< 60 ml / min	减量 1 个级别
< 40ml / min	停止化疗

c)　胃肠道毒性：化疗期间无需对 DDP 进行剂量调整。

d)　肝脏毒性：

①　停止化疗至血胆红素≤1.5×ULN 以及 AST 和/或 ALT ≤2.5×ULN 且 ALP ≤ 2.5×ULN。

②化疗必须停止如果血胆红素>2×ULN，或 AST/ALT > 5×ULN 和/或 ALP > 5×ULN。

肝脏毒性	白蛋白紫杉醇剂量调整
AST/ALT > 2.5–≤ 5×ULN<br>和/或 ALP > 2.5–≤ 5×ULN	减量 1 个级别
AST/ALT > 5×ULN 和/或 ALP > 5×ULN	停止化疗

④对于乙型肝炎病毒携带者，在化疗前及化疗期间需监测病毒的 DNA 变化。化疗前应在肝炎专科医生的指导下进行抗病毒治疗。

普特利单抗

普特利单抗，200mg每三周给药一次，每3周为1个治疗周期。以60分钟（±10分钟）静脉输注给药，对于无法耐受60分钟输注的受试者，输注时间最长可延长至120分钟（±15分钟）。给药延迟一般不超过3天，若特殊情况下，延迟超过3天不超过7天，建议下一次用药时，以这一次实际给药时间为准重新计算时间窗，后续给药时间窗仍然是3天。若延迟超过7天，建议本次不再给药，下一次按照计划给药日期再继续用药。延迟给药最长12周，自上一次给药时间计算，否则终止治疗。

- **普特利单抗剂量调整**

普特利单抗剂量调整（根据 NCI CTCAE 5.0 版对治疗中发生的 AE 进行分级，基于分级对普特利单抗的治疗进行调整，以管理潜在 irAE。）

✧　对于 1 级 irAE，无需进行剂量调整。

✧　对于 2 级 irAE，将暂停普特利单抗治疗，直至 2 级消退至 ≤1 级：

1)如果毒性恶化，将其视为 3 级或 4 级进行治疗。

2)如果毒性改善至基线水平，则在下次计划治疗访视时进行治疗。

3)如果 2 级 irAE 在 12 周内未消退至 ≤1 级或基线水平，将永久停用普特利单抗。

✧ 发生 3 级 irAE 时，可根据个体毒性永久停用普特利单抗治疗。

✧ 发生 4 级 irAE 时，必须永久停用普特利单抗治疗。

● 允许普特利单抗暂停的最长间隔时间是 12 周。如果在 12 周内不能恢复至可以重新使用普特利单抗的状态，则受试者永久停用普特利单抗，进入随访阶段。

①过敏反应和输注反应

抗体给药可能会引起过敏反应。因此，立即提供适当的药物和医疗设备来治疗急性过敏反应是至关重要的，研究人员必须接受培训以识别和治疗过敏反应。研究现场必须配备紧急救援队和设备，以及如有必要，将受试者纳入重症监护病房的能力。发生严重过敏反应的时候，受试者必须根据当地相关诊疗常规立刻给予紧急处理。受试者必须立刻给予肾上腺素和地塞米松，并立刻给予心电监护，可考虑抽取血清IgE标本。受试者必须立刻永久停药并退出研究治疗。受试者发生此类症状时必须立刻告知研究者。

输注反应的治疗调整指南

**CTCAE 级别**	**调整措施**
**1级 – 轻微**	一过性轻微反应，不建议中断输液和临床干预。将滴速减慢50%并密切观察任何恶化症状。必要时进行临床干预。
**2级 – 中度**	暂停普特利单抗治疗，并立刻给予系统性治疗（例如抗组胺药物，NSAID， 麻醉药物，静脉补液）；当输注反应缓解到0-1级时候重新给药并将滴速减低50%。 期间密切观察任何恶化症状。根据当地医疗常规采取恰当的治疗干预措施。
**3级– 严重**	立刻停止输液并移走输液管。如果重新给药，后续治疗的时候，输液时间至少2小时以上并且需要给予相关预防性用药（例如抗组胺药物和NSAID 药物），需要同时密切观察相关输注反应的临床症状。根据当地医疗常规采取恰当的治疗干预措施。
**4级– 危及生命并且需要紧急临床干预**	发生4级输注反应的患者必须立刻永久停药并退出研究。根据当地医疗常规采取恰当的治疗干预措施。

如果患者再次发生CTCAE 2级或以上的输注反应，患者必须立刻永久停药并退出研究。

肺毒性事件/肺炎控制指导原则

**事件**	**管理指导**
**1级**	•持续接受普特利单抗治疗，并密切监测<br>•重复评价影像检查<br>•考虑请肺部专家会诊

	•对于再次出现的肺部事件，按照3级或者4级的管理指导处理
**2级**	•暂停普特利单抗治疗<br>•考虑请肺部专家或者感染科专家会诊，并考虑支气管镜检查/经支气管肺泡灌洗<br>•开始每日口服泼尼松1–2 mg/kg/天或同等药物<br>•如果在8周内事件改善达0或1级，则重新开始普特利单抗治疗[a，b]<br>•如果在8周内事件不能改善达0或1级，则永久终止普特利单抗治疗<br>•对于再次出现的肺部事件，按照3级或者4级的管理指导处理
**3级或4级**	•永久终止普特利单抗治疗<br>•推荐支气管镜检查/BAL.<br>•开始每日口服泼尼松1–2 mg/kg/天或同等药物.<br>•如果事件在开始服用激素治疗48小时内没有改善，考虑免疫抑制类药物.<br>•如果事件改善达0或1级，激素可逐步减量，时间大于1个月

免疫相关甲状腺功能减退症和亢进症的控制指导原则

事件	管理指导
**无症状性甲状腺功能减退**	•继续普特利单抗 治疗<br>•开始使用甲状腺激素类替代激素治疗。<br>•每周监测一次促甲状腺激素（TSH）
**症状性甲状腺功能减退**	•暂停普特利单抗治疗<br>•开始使用甲状腺激素类替代激素治疗。<br>•每周监测一次TSH<br>•考虑内分泌科医生会诊<br>•症状得到控制，甲状腺功能恢复，可再次开始 普特利单抗治疗
**无症状性甲亢**	**TSH ≥ 0.1 mU/L 且< 0.5 mU/L:**继续 普特利单抗治疗；每四周监测一次TSH<br><br>**TSH < 0.1 mU/L:**按照症状性甲亢指导原则进行治疗
**症状性甲亢**	•暂停普特利单抗 治疗<br>•需要时开始抗甲状腺药物治疗，比如甲巯咪唑或者卡比马唑治疗<br>•考虑内分泌科医生会诊<br>•症状得到控制，甲状腺功能恢复，可再次开始 普特利单抗治疗<br>•出现危及生命的免疫相关的甲亢时，永久终止普特利单抗治疗

胰腺炎控制指导原则

事件	管理指导
**1级淀粉酶/脂肪酶升高**	• 继续普特利单抗治疗<br>• 在给药前，监测淀粉酶/脂肪酶水平

**2级淀粉酶/脂肪酶升高**	• 继续普特利单抗治疗<br>• 每周监测一次淀粉酶/脂肪酶<br>• 上述酶水平持续升高时（如超过3周），考虑口服泼尼松10 mg每日一次或等效剂量药物
**3级和4级淀粉酶/脂肪酶升高**	• 暂停普特利单抗治疗<br>• 消化科专家会诊<br>• 隔天监测淀粉酶/脂肪酶<br>• 如果症状未改善，考虑口服泼尼松1–2 mg/kg/每日或等效剂量药物<br>• 如果在8周内事件改善达0或1级，则重新开始普特利单抗治疗[a，b]<br>• 如果在8周内事件不能改善达0或1级，则永久终止普特利单抗治疗[a，b]<br>• 对于复发事件，则永久终止普特利单抗治疗[c]
**自身免疫性胰腺炎，2-3级**	• 暂停普特利单抗治疗<br>• 消化科专家会诊<br>• 静脉注射治疗（1～2 mg/kg/天甲基泼尼龙或等效剂量药物），症状改善后并转化为口服甾类药物（泼尼松1～2 mg/kg/天或同等药物）<br>• 如果在8周内事件改善达0或1级，则重新开始普特利单抗治疗[a，b]<br>• 如果在8周内事件不能改善达0或1级，则永久终止普特利单抗治疗[a，b]<br>• 对于复发事件，则永久终止普特利单抗治疗
**自身免疫性胰腺炎，4 级**	• 则永久终止普特利单抗治疗[c]<br>• 消化科专家会诊<br>• 静脉注射治疗（1～2 mg/kg/天甲基泼尼龙或等效剂量药物），症状改善后并转化为口服甾类药物（泼尼松1～2 mg/kg/天或同等药物<br>• 如果事件在开始服用激素治疗48小时内没有改善，考虑免疫抑制类药物.<br>• 如果事件改善达0或1级，激素可逐步减量，时间大于1个月

高血糖控制指导原则

事件	管理指导
**高血糖<br>1-2 级**	• 继续普特利单抗 治疗<br>• 需要时开始使用胰岛素治疗<br>• 监测血糖控制情况
**高血糖<br>3-4级**	• 暂停普特利单抗 治疗<br>• 开始使用胰岛素治疗<br>• 监测血糖控制情况<br>• 症状得到控制，血糖水平稳定，可再次开始普特利单抗治疗

肝毒性控制指导原则

事件	管理指导
**肝毒性事件<br>1级**	•继续 普特利单抗治疗.<br>•持续监测肝功能（LFT） 直到恢复到正常水平。
**肝毒性事件<br>2级**	•所有事件:<br>•密切监测LFT，直到恢复至基线水平.<br>•如果事件持续时间>5 天:<br>•暂停普特利单抗治疗<br>•开始每日口服泼尼松1–2 mg/kg/天或同等药物<br>•如果在8周内事件改善达0或1级，则重新开始普特利单抗治疗[a, b]<br>•如果在8周内事件不能改善达0或1级，则永久终止普特利单抗治疗
**肝毒性事件<br>3级或4级**	•永久终止普特利单抗治疗<br>•考虑请消化道专家会诊，进行肠胃道检查和肝穿刺活检，以明确肝损伤的原因<br>•开始每日口服泼尼松1–2 mg/kg/天或同等药物.<br>•如果事件在开始服用激素治疗48小时内没有改善，考虑免疫抑制类药物.<br>•如果事件改善达0或1级，激素可逐步减量，时间大于1个月

皮肤毒性控制指导原则

事件	管理指导
**皮肤毒性<br>1级**	• 继续普特利单抗治疗<br>• 考虑局部甾体类和/或其他对症治疗（如抗组胺药）
**皮肤毒性<br>2级**	• 继续普特利单抗治疗<br>• 考虑与皮肤科医生会诊

	• 使用局部甾体类药物。<br>• 若皮疹未恢复，则考虑使用更高效价的局部甾体类药物
**皮肤毒性 3级**	• 暂停普特利单抗治疗<br>• 考虑皮肤科医生会诊<br>• 开始口服泼尼松10 mg或同等药物治疗，若48～72小时后皮疹未恢复，增加到泼尼松1～2 mg/kg/天或同等药物<br>• 如果在8周内事件改善达0或1级，则重新开始普特利单抗治疗[a, b]<br>• 如果在8周内事件不能改善达0或1级，则永久终止普特利单抗治疗[a, b]
**皮肤毒性 4级**	• 永久终止普特利单抗治疗

结肠炎的控制指导原则

事件	管理指导
**1级腹泻或结肠炎**	• 继续普特利单抗治疗<br>• 开始对症治疗<br>• 如果症状持续7天以上，则建议实施内镜检查。<br>• 密切监测。
**2级腹泻或结肠炎**	• 暂停普特利单抗治疗<br>• 开始对症治疗<br>• 推荐消化科专家会诊<br>• 对于复发事件或者事件持续>5天，开始每日口服泼尼松1–2 mg/kg/天或同等药物.<br>• 如果在8周内事件改善达0或1级，则重新开始普特利单抗治疗[a, b]<br>• 如果在8周内事件不能改善达0或1级，则永久终止普特利单抗治疗[a, b]
**3级腹泻或结肠炎**	• 暂停普特利单抗治疗<br>• 消化科专家会诊，进行确证性活检<br>• 静脉注射治疗（1～2 mg/kg/天甲基泼尼龙或等效剂量药物），症状改善后并转化为口服甾类药物（泼尼松1～2 mg/kg/天或同等药物）<br>• 如果在8周内事件改善达0或1级，则重新开始普特利单抗治疗[a, b]<br>• 如果在8周内事件不能改善达0或1级，则永久终止普特利单抗治疗[a, b]
**4级腹泻或结肠炎**	• 永久终止普特利单抗治疗<br>• 消化科专家会诊，进行确证性活检<br>• 静脉注射治疗（1～2 mg/kg/天甲基泼尼龙或等效剂量药物），症状改善后并转化为口服甾类药物（泼尼松1～2 mg/kg/天或同等药物<br>• 如果事件在开始服用激素治疗48小时内没有改善，考虑免疫抑制

	类药物.<br>• 如果事件改善达0或1级，激素可逐步减量，时间大于1个月

神经毒性控制指导原则

事件	管理指导
**免疫相关神经毒性，1级**	• 继续普特利单抗治疗<br>• 评估病因
**免疫相关神经毒性，2级**	• 暂停普特利单抗治疗<br>• 评估病因.<br>• 按照相关机构指导原则进行治疗<br>• 如果在8周内事件改善达0或1级，则重新开始普特利单抗治疗[a, b]<br>• 如果在8周内事件不能改善达0或1级，则永久终止普特利单抗治疗[a, b]
**免疫相关神经毒性，3-4级**	• 永久终止普特利单抗治疗<br>• 按照相关机构指导原则进行治疗
**重症肌无力和Guillain-Barre综合症(任何级别)**	• 永久终止普特利单抗治疗<br>• 考虑神经科医生会诊<br>• 按照相关机构指导原则进行治疗<br>• 考虑1～2 mg/kg/天甲基泼尼龙静脉注射治疗或泼尼松口服或等效剂量药物

事件	管理指导
**眼部毒性 1级**	• 继续普特利单抗 治疗<br>• 强烈建议由眼科医师进行评价<br>• 采用局部用皮质激素滴眼剂和局部用免疫抑制疗法治疗<br>• 如果症状持续，按照2级毒性处理
**眼部毒性 2级**	• 暂停普特利单抗 治疗<br>• 强烈建议由眼科医师进行评价<br>• 采用局部用皮质激素滴眼剂和局部用免疫抑制疗法治疗<br>• 如果在8周内事件改善达0或1级，则重新开始普特利单抗治疗[a, b]<br>• 如果在8周内事件不能改善达0或1级，则永久终止普特利单抗治疗[a, b]
**眼部毒性 3级或4级**	• 永久终止普特利单抗治疗<br>• 强烈建议由眼科医师进行评价

	• 开始每日口服泼尼松1–2 mg/kg/天或同等药物.<br>• 如果事件改善达0或1级，激素可逐步减量，时间大于1个月

免疫相关骨关节病控制指导原则

事件 CTCAE 描述[1]	管理指导
**轻微炎性症状，红肿[2]，关节肿胀[3]**	•继续普特利单抗 治疗<br>•止痛剂：NSAIDs4-6周<br>•如果NSAIDs无效，考虑强的松7.5-15mg/天口服4-6周<br>•如果≤2关节受累，低剂量强的松(10 mg/天)或NSAIDs无效，可以考虑关节内注射皮质类固醇<br>•如果2-4周内无改善，升至2级管理<br>•进行风湿性疾病系列检查及功能性评估
**中度疼痛伴炎症表现，红肿，关节肿胀，日常使用工具受限**	•暂停普特利单抗 治疗<br>•风湿科会诊确定炎性关节炎，评估关节内注射的必要性及检查发现早期骨损体征<br>•强的松20mg/天，2-4周，增加至1mg/kg/天或等量，如果连续2-4周无好转，升级至3级管理<br>•如果症状改善，皮质类固醇逐渐减量超过4-8周，或恢复至1级
**严重疼痛伴炎症表现，红肿，关节肿胀，不可逆的关机损伤，功能丧失，日常生活不能自理**	•停止普特利单抗治疗<br>•风湿科会诊<br>•强的松1mg/kg/天或等量连续2-4周无好转，或恢复至1级<br>•考虑免疫抑制治疗[4]（如甲氨蝶呤[5]，柳氮磺胺吡啶，米氟米特），考虑抗细胞因子治疗（如TNF抑制剂）<br>•开始每日口服泼尼松1–2 mg/kg/天或同等药物.<br>•如果症状改善，皮质类固醇逐渐减量超过4-8周，或恢复至1级<br>•如4-6周内症状无改善，永久停止普特利单抗治疗

1.CTCAE 包括分别列出的关节炎，关节积液和关节痛，虽然这些事件有相同的症状如疼痛和影响日常活动

2. 睡眠或静止后关节僵直，于活动或热敷后症状好转.

3. 关节肿胀参考临床检查发现，可包含软组织肿胀，关节积液和滑膜炎.

4. 在开始药物治疗前，需进行乙肝，丙肝监测

5. 甲氨蝶呤需由15 mg 每周开始, 每日补充叶酸，滴定至25 mg 每周, 或如患者无法耐受口服可改为注射

6. 在开始抗细胞因子治疗前评估是否有活动性结核

免疫相关心肌炎控制指导原则

事件	管理指导
**免疫相关的心肌炎1级**	• 咨询心脏专科医生<br>• 根据专业指南开始治疗
**免疫相关的心肌炎2 级**	• 停止普特利单抗治疗12周<br>• 建议就诊心内科<br>• 头颅MRI检查<br>• 开始甲强龙静脉输注治疗1-2mg/kg/天，或等量或转换为强的松1-2 mg/kg/天口服或相应剂量至缓解<br>• 如果有临床指针用激素替代治疗<br>• 根据专科指南开始治疗并可以根据需要考虑抗心律失常药，临时起搏器ECMO或VAD<br>• 如果症状好转至1级或更好，重新开始普特利单抗治疗<br>• 如果症状未好转至1级或更好，永久停止普特利单抗治疗
**免疫相关的心肌炎3-4 级**	• 永久停止普特利单抗治疗咨询心脏专科医生<br>• 如果有临床指针用激素替代治疗<br>• 根据专科指南开始治疗并可以根据需要考虑抗心律失常药，临时起搏器ECMO或VAD<br>• 开始甲强龙静脉输注治疗1-2mg/kg/天，或等量或转换为强的松1-2 mg/kg/天口服或相应剂量至缓解[a b]<br>• 如果事件在使用皮质激素48小时内未缓解，考虑增加免疫抑制剂<br>• 如果症状改善，皮质类固醇逐渐减量超过1月，或恢复至1级

ECMO=体外膜肺氧合; VAD=心室辅助装置.

a如果已经开始使用皮质类固醇，则在普特利单抗可以恢复治疗之前，泼尼松或同等药物必须在≥ 1个月时间内逐渐减量至口服剂量≤10 mg/天以下。

b　普特利单抗可能会被暂停用药超过56天，以允许皮质类固醇减量至口服泼尼松或同等药物≤10mg/天的。

7、临床观察项目

- **药物安全性评价**：在研究过程中观察不良事件发生并根据NCI 不良事件常用术语标准5.0版（NCI CTCEA V5.0）评估，并判断与研究药物的关系，记录在CRF上。
- **治疗疗效评价**：根据RECIST评价方法，评价治疗疗效为CR、PR、SD、PD。以有效率（CR + PR）为标准，稳定（控制）率（CR +PR + SD）为标准。

- **生存质量评分**：通过欧洲癌症研究与治疗组织生活质量核心量表（EORTC QLQ-C30）及其头颈部肿瘤子量表（EORTC QLQ-H&N35），以评估症状和功能相对基线的变化。
- **功能保留**：通过UW-QOL量表和术后是否需要预防性气管切开评价。参考中国口腔颌面外科杂志2009年11月第7卷第6期发表的文章《应用UW-QOL评价口腔鳞癌患者的生存质量》，使用的评估量表为翻译并适当修改后的华盛顿大学生存质量问卷第4版（UW-QOL，V4.0），以评估舌鳞癌患者功能保留。

  UW-QOL第4版共12个特异性问题，3个综合问题，并允许患者自行添加量表中未充分涉及的问题。该12个条目分别为：疼痛外貌、娱乐、活力、吞咽、咀嚼、语言、肩功能、味觉、唾液、情绪和焦虑。每一条目分3到5个等级，采用Likert计分法，得分范围为0到100分，高分代表较好的生存质量；量表总分由各条目得分直接相加而得。UW-QOL主要针对手术患者设计，增加了唾液和味觉条目后，也可用于放疗患者。该量表的直译并适当修改版见附件7。

  术后是否需要预防性气管切开，也是本研究中功能保留的评价范围，尤其是肿瘤靠近气管，如果本新辅助方案疗效良好，缩瘤后可让部分患者不需要进行预防性气管切开，尽可能保留患者气管功能。由于口腔体积较小，口腔癌术后预防性气管切开是一种手术，主要是为了保持术后呼吸道通畅，防止伤口水肿或出血压迫呼吸道导致气道堵塞，出现呼吸困难。

8、患者生存获益评价

a) 客观缓解率（ORR）：定义为从入组到任何原因出组期间（最长2年）基于RECIST 1.1的最佳疗效达CR或PR有意义的收益的受试者占总受试者的比例。

b)主要病理缓解率（MPR）：定义为手术切除的原发灶和区域淋巴结内，存活肿瘤细胞比例≤10%。

c)病理完全缓解率（pCR）：定义为手术切除的原发灶和区域淋巴结内，未发现存活肿瘤细胞。

d) 无疾病进展生存期（Progression-Free of Survival, PFS）：患者定义为从入组到因任何原因引起肿瘤进展或死亡的时间间隔。以新病灶的出现作为进展的标准，进展的标志时间点为首次观察到可测定的新病灶的日期。

e) 总生存期（Overall Survival, OS）：是指从入组开始至因任何原因引起死亡的时间。当记录至死亡之前的失访患者，截止到最后一次有记录的与患者接触的时间。

f) 可手术或同步放化疗转化率：经过免疫联合化疗新辅助治疗后，转化为可手术切除以及术后放疗或直接同步放化疗患者的比例，即可手术切除患者+直接同步放化疗患者/总入组患者。

g) 生活质量评分：使用的评估量表包括欧洲癌症研究与治疗组织生活质量核心量表（EORTC QLQ-C30）及其头颈部肿瘤子量表（EORTC QLQ-H&N35），以评估症状和功能相对基线的变化。

g) 功能保留：通过适当修改版UW-QOL量表和术后是否需要预防性气管切开评价，详见第7部分及附件7。

9、不良事件

9.1 定义：

自受试者签署知情同意书并入选研究后至末次访视之间所发生的任何不利医疗事件，无论与研究药物是否有因果关系，均判定为不良事件。

不良事件包括如下：

- 所有可疑的不良药物反应。
- 所有由于药物过量、滥用、停药、过敏或毒性产生的反应。
- 明显无关的疾病，包括先前存在疾病的加重。
- 受伤或事故，在备注记录此事故的结果。
- 生理检查或体格检查发现的异常，且需要临床治疗或作进一步检查者。

9.2 不良事件记录

研究医师应用简洁的医学术语报告医师直接观察到的或受试者自发报告的所有不良事件。另外，治疗开始的每次就诊均需向患者询问不良事件情况，如实填写不良事件记录表，记录不良事件的发生时间、严重程度、持续时间、采取的措施和转归。不良事件应记录在CRF的不良事件表中。

9.3 不良事件严重程度判定标准

在填写CRF的不良事件表时，研究者将使用轻度、中度、重度来描述不良事件的强度。为统一标准，事件强度的分级如下：

轻度：不影响受试者的正常功能。

中度：一定程度上影响到受试者的正常功能。

重度：明显影响受试者的正常功能。

注意区别不良事件的严重程度和强度。重度用来描述强度，不一定是SAE。例如头痛可能在强度上表现为重度，但不能列入SAE，除非它符合SAE标准。

9.4 不良事件与研究药物关系的判断标准

参考"关于发布个例药品不良反应收集和报告指导原则的通告（2018 年第 131 号）"，结合临床，采用七分法进行判断，参考标准如下：

1) 肯定有关：用药与不良反应的发生存在合理的时间关系；停药后反应消失或迅速减轻及好转（即去激发阳性）；再次用药不良反应再次出现（即再激发阳性），并可能明显加重；同时有说明书或文献资料佐证；并已排除原患疾病等其他混杂因素影响。
2) 很可能有关：无重复用药史，余同"肯定"，或虽然有合并用药，但基本可排除合并用药导致不良反应发生的可能性。
3) 可能有关：用药与反应发生时间关系密切，同时有文献资料佐证；但引发不良反应的药品不止一种，或不能排除原患疾病病情进展因素。
4) 可能无关：不良反应与用药时间相关性不密切，临床表现与该药已知的不良反应不相吻合，原患疾病发展同样可能有类似的临床表现。
5) 待评价：报表内容填写不齐全，等待补充后再评价，或因果关系难以定论，缺乏文献资料佐证。
6) 无法评价：报表缺项太多，因果关系难以定论，资料又无法获得。
7) 肯定无关：药物与不良事件不存在合理的可能性，即因果关系可以排除。

以上 7 级评价可通过下表表示：

关联性评价	时间相关性	是否已知	去激发	再激发	其他解释
肯定有关	+	+	+	+	-
很可能有关	+	+	+	?	-
可能有关	+	±	±?	?	±?
可能无关	-	-	±?	?	±?
肯定无关	研究药物与不良事件不存在合理的可能性，即因果关系可以排除。				
待评价	缺乏必须信息，需要补充材料才能评价				
无法判断	缺乏必须信息，并无法获得补充资料				

1) ＋表示肯定或阳性；－表示否定或阴性；±表示难以判断；？表示不明。
2) 时间相关性：用药与不良反应的出现有无合理的时间关系。

3) 是否已知：不良反应是否符合该药已知的不良反应类型。
4) 去激发：停药或减量后，不良反应是否消失或减轻。
5) 再激发：再次使用可疑药品是否再次出现同样的不良反应。

其他解释：不良反应是否可用并用药品的作用、患者病情的进展、其他治疗的影响来解释

9.5 严重不良事件以及记录与报告

不良事件符合下面一条或以上标准时归为严重不良事件（Serious Adverse Events, SAE）：

- 导致死亡；
- 危及生命；
- 需要住院治疗或延长住院时间；
- 导致持续的或严重的残疾或功能不全；
- 先天性畸形或出生缺陷。

有些还没有导致死亡、生命危险或需住院的医疗事件，经过适当的医学判断，认为其可能对患者或受试者造成危害或需药物或外科手术治疗以避免上述情况的发生时，也应视为SAE。

临床研究过程中受试者发生严重不良事件时，临床医生在30分钟内向中心负责人报告不良事件的类型和严重程度。接到报告后24小时内，需向南华大学附属第二医院GCP办公室主任、医务处长及伦理委员会主任汇报严重不良事件的情况，填写相应表格并签字和签署日期。

10、数据管理与统计分析

10.1 病例报告表的填写与移交

病例报告表由研究者填写，每个入选病例必须完成病例报告表。完成的病例报告表由临床监查员审查后，第一联移交数据管理员，进行数据录入与管理工作。

10.2 数据的录入与修改

数据录入与管理由统计学负责人指定的数据管理员负责。数据管理员采用SPSS24.0统计软件进行数据录入与管理。为保证数据的准确性，应由两个数据录入员独立进行双份录入并校对。

对病例报告表中存在的疑问，数据管理员做成疑问解答表(DRQ)，通过临床监查员向研究者发出询问，研究者应尽快解答并返回，数据管理员根据研究者的回答进行数据修改、确认与录入，必要时可以再次发出DRQ。

10.3 数据锁定

在确认建立的数据库正确后，由统计分析人员对数据进行锁定。锁定后的数据文件不再做改动。数据锁定之后发现的问题，经确认后在统计分析程序中进行修正。

10.4 统计分析

统计分析采用SAS 9.2或以上版本进行。如无特殊说明，对于定性指标，用频数、百分比来描述。对于定量指标，计算均值、标准差、中位数、最小值和最大值，时间-事件数据采用Kaplan-Meier法估计中位时间及其总体的95%置信区间。如无特殊说明，统计分析的检验水准取α=0.05双侧。

分析内容包括：

- 一般资料分析

  评价年龄、性别、分期等一般资料在各组间的分布与均衡性。

- 不良反应分析

  采用NCI-CTC5.0标准评价各组近期毒副反应及后遗症、并发症等。

- 诱导化疗及短期疗效分析

  按RECIST疗效评价标准评价，记录为CR、PR、SD或PD。

- 远期疗效分析

  根据随访结果计算各组患者的2、3、及5年总生存率、无进展生存率、无局部区域复发存率及无远处转移生存率。

- 分层分析

  按年龄、性别、分期等进行分层分析

- 每次的计量资料将采用均数±标准差进行统计描述。治疗前后的变化采用配对t检验；
- 每次的计数资料采用频数进行统计描述。治疗前后的变化采用Chi-Square检验。
- 总脱落率和由不良事件而产生的脱落率的比较将采用Chi-Square检验。
- 安全性和耐受性评价由不良反应和实验室数据的变化构成。
- 对不良事件进行描述统计，列出发生事件、持续时间、严重程度、和药物的关系、以及其转归；并对不良事件的发生率进行Chi-Square检验。
- 生存指标的分析使用Kaplan-Meier方法和Log-rank检验。

11、伦理学要求和受试者知情同意书

本临床研究已经获得湖南省肿瘤医院伦理道德委员会的书面同意。根据GCP的指导原则，研究方案的任何修改需要伦理道德委员会的同意。如这些修改可能会给患者带来危害，应更

新知情同意书的内容，并需征得参加研究患者的签名同意。本临床研究遵循《赫尔辛基宣言》和中国有关临床研究的规范和法规。

在受试者入选本研究前，研究医师必须向受试者或受试者法定代理人全面地介绍本临床研究的目的、程序和可能的风险。应让受试者知道他们有权在任何时间、任何情况下退出本研究。入组前每位受试者或受试者法定代理人必须签署知情同意书，一式两份（一份为原件、另一份为副本）。研究医师有责任在每位受试者进入研究之前获得知情同意书，并作为研究档案保存。

12、资料保存

为保证国家食品药品监督管理局和申办者对临床研究的评价与监督，临床研究单位应完整保存临床研究全过程的所有研究资料和原始记录，包括对所有受试者的确认(能有效地核对不同的记录资料，如CRF和医院原始记录)、所有的有签名的知情同意书、CRF、药品分发的详细记录等。研究单位应保存这些临床资料至临床结束后5年。

本临床研究的所有资料，所有权属于申报单位，除国家药品监督管理局要求外，未经申办者书面同意，研究者不得以任何形式提供给第三者。

表 2：筛选开始到治疗期间的研究流程

		新辅助治疗									手术治疗						辅助治疗及随访									治疗结束/退出治疗
**周期**		**1**			**2**			**3**			**1**			**2**			**1**			**…**			**18**			
**天数**	**-14 ~0**	**D 1**	**D 8**	**D 15**	**D 22**	**D 29**	**D 36**	**D 43**	**D 50**	**D 57**	**D 64**	**D 71**	**D 78**	**D 85**	**D 92**	**D 99**	**D 106**	**D 113**	**D 120**	**D 127**	**D 134**	**D 141**	**D 302**	**D 309**	**D 316**	
通过 RECIST 1.1 行肿瘤影像学评估(颈部增强 MRI)	**X**						**X**									**X**										**X**
胸腹部 CT（平扫+增强）或 PET-CT	**X**																									
知情同意书	**X**																									
既往病史、治疗史、药物史[a]	**X**																									
体格检查	**X**	**X**			**X**			**X**			**X**			**X**			**X**			**X**			**X**			**X**
生命体征[b]	**X**	**X**			**X**			**X**			**X**			**X**			**X**			**X**			**X**			**X**
ECOG 体能状态[c]	**X**	**X**			**X**			**X**			**X**			**X**			**X**			**X**			**X**			**X**
ECG	**X**	**X**			**X**			**X**			**X**			**X**			**X**			**X**			**X**			
超声心动图	**X**																									
凝血功能	**X**							**X**			**X**			**X**			**X**			**X**			**X**			
血常规、生化常规、尿常规	**X**	**X**	**X**	**X**	**X**	**X**	**X**	**X**	**X**	**X**	**X**	**X**	**X**	**X**	**X**	**X**	**X**	**X**	**X**	**X**	**X**	**X**	**X**	**X**	**X**	**X**
甲状腺功能检查	**X**																**X**			**X**			**X**			
乙型、丙型肝炎、HIV[d]	**X**	**如有异常定期检查**																								
血浆 HPV-DNA	**X**	**X**			**X**			**X**			**X**			**X**			**X**			**X**			**X**			**X**
淋巴细胞亚群 4 项	**X**	**X**									**X**															**X**
电子鼻咽镜检查	**X**				**X**			**X**			**X**			**X**			**X**			**X**			**X**			**X**
生活质量评价	**X**				**X**			**X**			**X**			**X**			**X**			**X**			**X**			**X**
合并用药/伴随治疗[e]	**X**	**X**	**X**	**X**	**X**	**X**	**X**	**X**	**X**	**X**	**X**	**X**	**X**	**X**	**X**	**X**	**X**	**X**	**X**	**X**	**X**	**X**	**X**	**X**	**X**	**X**
AE/SAE 评估	**X**							**X**	**X**	**X**	**X**	**X**	**X**	**X**	**X**	**X**	**X**	**X**	**X**	**X**	**X**	**X**	**X**	**X**	**X**	**X**
研究中止记录			**随时记录并根据规定予以报告**																							
研究结束记录																										**X**

缩略语：AE：不良事件；ECG：心电图；ECOG：美国东部肿瘤协作组；HIV：人类免疫缺陷病毒；RECIST：实体瘤疗效评价标准；SAE：严重不良事件；T3：三碘甲状腺原氨酸；T4：甲状腺素；TSH：促甲状腺激素。

注释：

a 既往病史

采集签署 ICF 前开始的、本适应症外的所有既往病史、药物过敏史、吸烟史及饮酒史；

b 生命体征包含坐位血压、脉搏、呼吸、体温；

c 是从患者的体力来了解其一般健康状况和对治疗耐受能力的指标。分为 0 至 5 分，共 6 个等级。打分低，患者的健康状况良好。一般认为打分为 3 以上的患者不适宜进行化疗；

d 乙肝病毒筛查项目包括乙肝表面抗原（HBsAg）、乙肝表面抗体（HBsAb）［如需要，接受乙型肝炎病毒（HBV）脱氧核糖核酸（DNA）载量检查 HBV DNA 检测］、丙肝核心抗体（HBcAb）［如果抗体测试为阳性，则进行丙型肝炎核糖核酸（RNA）定性检测］和 HIV-Ab 抗体；

e 合并用药/伴随治疗：记录筛选前前 30 天内及治疗过程中的本适应症以外的其他疾病治疗用药和伴随治疗。

附录

附录 1 肿瘤评价-新实体瘤缓解评价标准-版本 1.1

1 基线肿瘤的可测量性

1.1 定义

● 基线时，将肿瘤病灶/淋巴结分成可测或不可测两类，内容如下：

1.1.1 可测量的肿瘤病灶

必须至少准确测量肿瘤病灶的一个维度（记录测量平面的最大直径），其最小尺寸为：

● CT 或MRI 扫描：10 mm(CT/MRI 扫描层厚/间隔不超过5 mm)

● 临床检查的卡尺测量：10 mm(无法用卡尺准确测量的病灶应记录为不可测量)。

● 胸部X-线检查：20 mm。

恶性淋巴结：

采用CT 扫描评估时，淋巴结在短轴上的直径必须为15 mm（建议CT 扫描切片的厚度不超过5 mm），才可视为病理学增大且为可测量。在基线时和随访期间，仅对短轴进行测量和随访。也可参见下文第2.2 节“基线靶和非靶病灶的记录”，了解相关淋巴结测量的信息。

1.1.2 不可测量的肿瘤病灶

不可测量的肿瘤病灶包括小病灶（最大直径＜10 mm 或者短轴≥10 且＜15 mm 的病理性淋巴结）以及真正的不可测量病灶。真正的不可测的病灶包括：软脑膜疾病、腹水、胸腔或心包积液、炎性乳癌、皮肤或肺淋巴管转移癌、体检发现但不能通过用可重现成像技术所测量的腹部包块/腹部器官肿大。

1.1.3 关于病灶测量的特殊考虑事项

对于骨性病灶、囊性病灶和既往局部治疗的病灶需要特别注明：

骨性病灶：

● 骨扫描、PET 扫描或平片都不适合于测量骨性病灶， 但可用于证实骨性病灶的存在或消失。

● 对于含有可识别的软组织成分的溶解性骨病灶或者混合性溶解-增生病灶，其软组织成分可以通过横断层面成像技术如CT 或者MRI 进行评价，如果软组织成分符合上述可测量性定义，即可视为可测量病灶。

● 成骨病灶是不可测量的。

囊性病灶：

● 符合影像学单纯性囊肿定义标准的病灶不应被视为恶性病灶（既不是可测量病灶也不是不可测量病灶），因为按照定义这些病变只是单纯性囊肿。

● 如果符合以上可测量定义且为囊性转移的‘囊性病灶’可视为可测量病灶。然而，如果同一

患者存在了非囊性病灶，这些病灶将优先选为靶病灶。

既往局部接受治疗的病灶：

- 以往放疗过，或者接受过其他局部区域性治疗的肿瘤病灶一般不被视为可测量病灶，除非该病灶出现明确进展。研究方案中应详细规定将这类病灶视为可测量病灶的条件。

1.2 靶病灶：　测量方法的说明

1.2.1 病灶的测量

临床评估使用卡尺测量，所有的测量值都应该以米为单位进行记录。应尽可能在距治疗开始较近的时间内进行所有基线的评估，但不得在治疗开始前四周或者更长时间前进行。

1.2.2 评价方法

在基线和之后的试验期间必须使用相同的评估方法和相同的技术来评估每个满足要求且需要报告的病灶。应始终优先选择以成像为基础的评估方法。临床病灶：仅当临床病灶位于浅表且用卡尺测量的直径≧10mm时（如皮肤结节），才可以视为可测量病灶。对于皮肤病灶，推荐使用彩色拍照，包括使用标尺来估算病灶的大小。

胸部X 光检查: 当肿瘤进展作为重要研究终点时，胸部CT 要优于胸部X 线，因为CT比X 线更敏感，尤其是在识别新病灶方面。然而，胸部X-线上的病灶，如果它们可清楚界定并且肺部充气良好时，可视为可测量性病灶。

CT，*MRI*：CT 是目前用于疗效评价最佳的且可重现的成像方法。该指南对可测量性的定义建立在CT 扫描层厚度≤5mm 的基础上。当 CT 的层厚大于5mm，可测量病灶的最小尺寸应为层厚的2 倍。 MRI 也可以使用。

如果在入组前已知患者因为过敏或肾功能不全而不能接受IV 造影剂的CT 扫描，应该根据研究的肿瘤类型和疾病的解剖位置指导是否在基线和研究期间使用无造影剂CT 或MRI（无IV 造影剂）来评估受试者。对于在基线造影剂CT 扫描后发生造影剂禁忌的患者，也应该根据肿瘤类型、疾病解剖部位决定是否进行无造影剂CT或MRI 扫描（增强或平扫）。同时，应进行优化，以在有可能时与既往研究作比较。应该与放射科医师对每例病例进行讨论，决定替换成其他方法是否可行，如果不可行，应该认为患者从此时开始不可评估。

超声波：　超声波不能用于评价病灶大小，不能用作测量方法。

内窥镜检查、*腹腔镜检查*、*肿瘤标记物*、*细胞学*、*组织学*：　一般不建议使用这些技术进行客观肿瘤评估，但是也会根据研究设计来确定。

2 肿瘤缓解评估

2.1. 肿瘤总负荷与可测量疾病的评价

为客观缓解或以后疾病进展的评价，有必要估计基线水平时肿瘤的总负荷，并将其作为参考与后续的测量进行比较。可测量疾病定义为至少存在一个可测量的病灶（参见上文第1.1.1 节详述的定义）。

2.2. “靶”病灶及“非靶”病灶在基线的记录

当基线有一个以上可测量病灶存在时，应将能代表所有受累器官的所有病灶（最多总共5个病灶，每个器官最多2 个病灶）确定为靶病灶，并在基线期予以记录和测量。

这意味着当患者只有一个或两个器官部位受累时，分别最多记录两个（一个部位）和四个（两个部位）病灶。该器官中的其他病灶（虽然可测量）将被记录为不可测病灶（即使CT 扫描的尺寸大于10 mm）。

应该根据尺寸（有最长径的病灶）选择靶病灶，靶病灶应能代表所有受累器官，并在重复测量时能重复出现。还有一种可能是，有时最大病灶不适合重复性测量，在这种情况下，应该选择下一个最大的且重复测量的病灶。

淋巴结需要特别提及，因为淋巴结是可以通过成像观察到的正常解剖结构（即使没有被肿瘤累及）。按照第1.1.1 节所述，被定义为可测量且可确定为靶病灶的病理性结节必须符合CT 扫描的短径＞15 mm 的标准。只有这些结节的短径长度会参与基线加和。结节的短径是放射科医师在判断结节是否被实体瘤累及时通常会使用的直径。结节尺寸在通常情况下报告为获取图像的平面的两个维度（对于 CT 扫描这几乎一直是轴平面；对于 MRI采集平面可能是轴向、矢状或冠状面）。这些测量值中的较小者即为短径。例如，被报告为 20 mm x 30 mm 的腹部结节，其短径是 20 mm，符合恶性可测量结节的条件。在这个实例中，20 mm 应记录为结节测量值。所有其他病理性结节（短径≥10 mm 但＜15mm）应视为非靶病灶。短径＜10 mm 的结节应视为非病理性结节，不应该记录或跟踪。

计算所有目标病灶的直径总和（非结节病变的最长直径，结节病灶的短径），然后将其报告为基线直径总和。如果在总和中包括淋巴结，如上所述，在总和中只增加短径。基线直径总和将作为参考，来进一步描述任何客观的肿瘤在可测量直径上变化。基线时所有其他病变（或疾病部位），包括病理性淋巴结，应定义为非靶病灶，也进行记

录。不需要测量结果，这些病灶需注明“有”、“无”、或极罕见的“明确进展”（也参见第**2.3.4** 节）。

此外，在病例报告表上有可能将累及同一器官的多个非靶病灶，应进行记录（例如，“多个扩大的骨盆淋巴结”或者“多个肝转移”）。

2.3. 缓解标准

本部分定义了确定目标病灶客观肿瘤缓解的标准。

2.3.1. 靶病灶的评估

- 完全缓解（*CR*）：所有靶病灶消失。所有病理性淋巴结（不论靶或非靶病灶）的短径必须缩短至＜10 mm。
- 部分缓解（*PR*）：以基线直径总和为参照，靶病灶的直径总和至少降低30%。
- 进展性疾病（*PD*）：以研究最小的直径总和为参照（包括基线），目标病灶的直径总和至少增加20%。除了相对增加20%，该总和也必须至少有5 mm的绝对增加。（注意： 一个或多个新病灶的出现也被视为进展）。

● 疾病稳定（*SD*）：靶病灶缩小的程度未达到部分缓解，增加的程度也未达到PD，介于两者之间。

2.3.2. 靶病灶评估的特别说明

淋巴结：即使鉴定为靶病灶的淋巴结在研究中缩小至10mm 以内，每次测量时也应该记录与基线对应的实际短直径的值（与基线测量时的解剖平面一致）。这表示当将淋巴结作为靶病灶时，即使符合完全缓解标准，病灶“总和”也不可能为零，因为正常淋巴结的

短轴就定义为＜10 mm。

“太小而无法测量”的靶病灶：　在研究中，所有在基线期记录的病灶（淋巴结病灶和非淋巴结病灶），即使非常小（如2 mm），也应该在每次后续评估时记录其实际测量值。

然而，有时在基线时被记录为靶病灶的病灶或淋巴结变得太小以至于CT 扫描图十分模糊，放射科医师可能觉得无法对其进行准确测量，而将这些病灶或淋巴结报告为“太小而无法测量”。　如果发生这种情况，在病例报告表中记录一个测量值非常重要：

● 如果放射科医师认为病灶有可能消失，测量值应记录为0 mm。

● 如果认为病灶存在，且过于模糊，太小而难以测量，应记录为默认值5 mm并标明是BML（低于可测限）（注意：　这条规则不太可能会用于淋巴结，因为正常淋巴结的尺寸一般可以界定，而且周围经常包围着脂肪（如腹膜后腔中的淋巴结）；但是，如果淋巴结确实存在且看起来模糊，因为太小而无法测量，在这种情况下也应该记录为默认值5 mm 并标明BML。）

然而，要重申一下，如果放射科医师可以提供实际的测量值，应予以记录，即使该测量值小于5 mm，而且在这种情况下不应该勾选BML（BML相当于小于符号＜）。

治疗中分裂或融合的病灶：　当非淋巴结病灶“分裂”时，应该在计算靶病灶总和时加入各个破碎部分的最长径。类似地，当病灶融合时，如果两病灶之间的平面仍然保留下来，这样有助于获得每个单独病灶的最大直径测量值。如果病灶真正发生融合，以致这些病灶不再是分离的，那么这种情况下的最长径矢量应该是“融合病灶”的最长径。

2.3.3. 非靶病灶的评估

本部分定义了用于确定非靶病灶群的肿瘤缓解标准。虽然有些非靶病灶实际上可以测量，但是这些病灶并不需要测量，只需在方案规定的时间点进行定性评估。

完全缓解（*CR*）：所有非靶病灶消失（如适用，肿瘤标记物水平恢复正常）。所有淋巴结必须达到非病理性大小（短轴＜10 mm）。

非完全缓解（*Non-CR*）/非疾病进展（*Non-PD*）：持续存在一个或多个非靶病灶和/或肿瘤标记物水平维持在正常值范围以上。

疾病进展（*PD*）：　现存的非靶病灶明确进展（参见第2.3.4 节）。出现一处或多处的新病灶也被视为进展。

2.3.4. 评估非靶疾病进展的注意事项

当患者还有可测量病变时：**在这种情况下，要根据非靶疾病来判断是否达到“明确进展”的**

标准，还需非靶疾病实质性恶化到达这样的总体水平（即使靶疾病出现SD或PR），即总体肿瘤负荷增加程度足以终止治疗。一个或多个非靶病灶尺寸上稍微增加通常不足以达到“明确进展”状态。当靶疾病出现SD 或PR 时，仅基于非靶疾病的变化就确定为总体进展的情形极少。

当患者只有不可测量病变时：这样的情况发生在具有可测量疾病并非研究入选标准的某些III期临床试验中。整体评估还是参照上文标准，但是在这种情况下，没有可测量病灶的数据，不可测量病灶的负荷增加的不容易解释。当非靶疾病改变导致整体疾病负荷增加的程度相当于靶疾病出现疾病进展时，依据非靶疾病做出明确进展的定义，需要建立一种有效的检测方法来评估。例如：肿瘤负荷增量相对于“体积”额外增加73%（相当于可测量病灶的直径增加了20%）， 又比如胸腔积液从“微量”到“大量”，淋巴管炎疾病从局部到普遍扩散，或者在研究方案中被描述为“足以要求调整治疗方案”。 若出现“明确进展”，患者在当时应被认为出现总体PD。理想情况下应该有客观标准用于不可测量疾病，而这种病灶的特殊性质决定了这不可能，因此，增量必须是可靠的。

2.3.5. 新病灶

新的恶性病灶的出现意味着疾病进展；所以，对于新病灶检测工作做出一些特别说明很重要。由于没有具体的标准可用来确定新放射学病灶，而新病灶的检测结果必须明确，比如不能归因于扫描技术的差异、影像学方法的变化或被认为代表并非肿瘤的结果。（例如，某些“新”骨骼病灶可能仅仅是先前已有病灶的愈合或突然复发）。当患者的基线病灶表现出部分或完全缓解时，这一点尤为重要。例如，肝脏病灶的坏死可能在CT 扫描报告中被报告为“新囊性病灶”，而实际并不是这样的。

基线时在解剖部位并未扫描出而在随访研究时发现的病灶被视为新病灶，且将显示疾病进展。如果新病灶不明确，例如尺寸过小，但它确实为新疾病，则需要在继续进行的治疗和随访评估中予以阐明。如果重复扫描证实确实为新病灶，那么应以初始扫描日期为疾病进展日期。

2.4 缓解评估

2.4.1 时间点缓解（总缓解）

假定在研究方案规定的每个时间点都进行一次缓解评估。表1 中总结了基线时有可测量病灶的患者在每个时间点的总体缓解情况的结果。

当患者只有不可测量（因此为非靶）疾病时，应使用表2。

表**1 缓解时间点-有靶病灶（伴/不伴非靶病灶）的患者**

靶病灶	非靶病灶	新病灶	总体缓解
CR	CR	否	CR
CR	非 CP/非 PD	否	PR
CR	未评估	否	PR
PR	非 PD 或未全部评估	否	PR
SD	非 PD 或未全部评估	否	SD
未全部评估	非 PD	否	NE
PD	任何	是或否	PD
任何	PD	是或否	PD
任何	任何	是	PD

CR =完全缓解，PR=部分缓解，SD =疾病稳定，PD =疾病进展，NE =不可评估。

表2 缓解时间点-仅有非靶病灶的患者

非靶病灶	新病灶	总缓解
CR	否	CR
非 CR/非 PD	否	非 CR/非 PD[a]
未全部评价	否	NE
明确 PD	是或否	PD
任何	是	PD

CR=完全缓解，PD= 疾病进展，和 NE=不可评估。

a 对于非靶病灶，“非 CP/非 PD”优先于“疾病稳定”，因为在一些试验越来越多地使用 SD 作为疗效评估终,因此不建议在所有病灶都不能测量时归为“疾病稳定”类别。

2.4.2 评估缺失和不可评估的说明

如果在某一个特定时间点,患者没有任何影 资料和其他测 指标,那么该患者在该时间 点是不可评估的。 如果患者在一次评估中只进行了一部分病灶的测量，那么通常情况下该时间点也被认为不可评估的,除非有令人信服的证据证明单个缺失病灶的影响不会影响所指定的时间点疗效反应评价。 这最有可能在疾病进展的情况下发生。

例如,如果一例患者在基线时测得的三个病灶,总和为 50 mm,在随访期间只对两个病灶进行了评估,但总和为 80 mm,则我们认为该患者已达到 PD 状态，这与缺失病灶的影响无关。

一处或多处靶病灶如果因为没有扫描而没有进行评估,或者因为图像质量差或视野受限而不可评估，那么靶病灶的疗效评估应该为“无法评估”，因为患者不可评估。类似地, 如果一处或多处非靶病灶被指示为“未评估”，那么非靶病灶的反应应为“无法评估” (除非发生明显进

展)。 如果靶病灶反应或非靶反应为“无法评估”，那么总体疗效反应应为“无法评估”(除非这是疾病进展的明显证据)，因为这种情况与该时间点不可评 估的情况是等同的。

表 3 需要确认时的最佳总体缓解

第一个时间点<br>总体缓解	后续时间点<br>总体缓解	最佳总体缓解
CR	CR	CR
CR	PR	SD，PD 或者 PR*
CR	SD	若达到 SD 持续时间的最低标准，则为 SD，否则为 PD
CR	PD	若达到 SD 持续时间的最低标准，则为 SD，否则为 PD
CR	NE	若达到 SD 持续时间的最低标准，则为 SD，否则为 NE
PR	CR	PR
PR	PR	PR
PR	SD	SD
PR	PD	若达到 SD 持续时间的最低标准，则为 SD 否则为 PD
PR	NE	若达到 SD 持续时间的最低标准，则为 SD 否则为 NE
NE	NE	NE

CR =完全缓解，PR =部分缓解，SD =疾病稳定，PD =疾病进展，和 NE =不可评估。
a 如果在第一个时间点确实为完全缓解，然后在后续时间点出现疾病，即使该疾病满足相对于基线的部分缓解标准，则在这个时间点评估为疾病进展（因为疾病在完全缓解后复发）。
最佳缓解取决于是否满足疾病稳定的最短持续时间。 然而，有些情况下患者评估为“完全缓解”，但随后的扫描提示小病灶很可能仍然存在，则实际上患者在第一个时间点为部分缓解而非完全缓解。 在这些情况下，应将最初的 CR 更改为 PR，最佳缓解状态为 PR。

3 关于疗效评估的特殊说明

当淋巴结疾病已被列入靶病灶,而且淋巴结减少到“正常”的大小(<10 mm),它们在扫描记录仍可有一个测量数据。即使淋巴结已恢复正常,若进展以淋巴结大小的增加为依据,为了不夸大效果,这些测量结果仍应该记录。如前所述，这意味着完全缓解的患者在病例报告表(eCRF)中的总和不一定为“零”。

因整体健康状况恶化需中断治疗的患者,且缺少该时间点疾病进展客观证据的患者应报告为“症状性恶化”。即使在中断治疗以后,也应积极证实其疾病的客观进展。 症状性恶 化不是客观疗效的一种 述, 只是停止治疗的原因。 这类患者的客观缓解状况应通过对 靶疾病和非靶疾病的评估来决定,如表 1,表 2 和表 3 所示。

对于不明确的进展发现(例如非常小且不确定的新发病灶;已有病灶中的囊性或坏死改变 等),治疗可以持续到下一次预定的评估。如果在下一个预定的评估确认为进展,进展日 期应该为怀疑出现进展的较早日期。

在实验入组的晚期疾病患者原发性疾病持续或部分存在时,原发肿瘤应适当的记录在靶病灶或非靶病灶中。 这样是为了避免当原发性疾病仍然存在但不可评估时,使用统计程序 对完整的总体疗效做出错误评价。

附录 2 ECOG 活动状态评分

根据美国东部肿瘤协作组(ECOG)活动状态分级和标准对患者进行分级如下:

体能评分	体力状态
0	活动能力完全正常，与起病前活动能力无任何差异
1	能自由走动及从事轻体力活动，包括一般家务或办公室工作，但不能从事较重的体力活动
2	能自由走动及生活自理，但已丧失工作能力，日间不少于一半时间可以起床活动
3	生活仅能部分自理，日间一半以上时间卧床或坐轮椅
4	完全残疾；生活不能自理；卧床不起
5	死亡

附件 3 免疫相关不良事件管理参考办法

以下关于任何免疫相关不良事件（irAE）诊断和处理的建议仅用作指导。本文件应与专家（在使用免疫药物治疗癌症方面经验丰富的专科医师）的临床判断以及各机构指导原则或政策结合使用，并建议参考最新的免疫相关不良事件的管理指南/共识。

用于诊断 irAE 的标准包括血液检查、诊断性影像学检查、组织病理学和微生物学评估，以排除其他原因如感染、疾病进展和伴随药物的不良反应。除了这些检查结果外，在进行 irAE 诊断时还应考虑以下因素：

- 使用普特利单抗与不良事件之间是否有时序关系？
- 患者在普特利单抗停药后的反应如何？
- 在重新开始普特利单抗治疗后，事件是否复发？
- 使用皮质类固醇治疗后是否出现临床缓解？
- 事件是否为自身免疫性内分泌疾病？
- 疾病进展或其他诊断是否为更合理的解释？

免疫相关不良事件的治疗

- 免疫相关不良事件可迅速发展；根据适当情况，可能需要中断研究治疗、密切监测、及时进行诊断相关检查和治疗干预
- 免疫相关不良事件在给予免疫抑制治疗后应迅速改善。如果没有出现这种情况，请检查诊断，请专家进一步给予建议，并联系研究的医学监查员
- 对于一些快速消退的 3 级毒性，如果有证据表明对研究治疗产生了临床缓解，在与研究的医学监查员协商后，可以考虑恢复研究药物治疗
- 下表中的类固醇剂量是指口服或静脉给予（甲基）泼尼松龙的剂量。可替换等效剂量的其他皮质类固醇。对于类固醇难治性irAE，需考虑使用类固醇保留剂（例如霉酚酸酯[MMF]）
- 如果患者长期接受免疫抑制治疗，则考虑预防治疗性抗生素以预防机会性感染

免疫相关的不良事件	可能发现的征兆和症状	普通诊断与鉴别诊断	推荐管理办法	剂量调整
肺炎	有症状的：呼吸困难、咳嗽、胸膜炎性胸痛，缺氧<br>无症状的：状似严重细菌性肺炎	• 肺部放射影像评价<br>• 检视支气管镜检时使用的组织和灌洗液的样本以排查传染性病原体，以及对其进行病	轻度（1 级）：密切观察，如加重，按照严重级别处理。	可考虑延迟给药，并每 2-3 天进行评估，如无加重，可继续治疗。
			中度（2 级）：施用糖皮质激素强的松或同类药，给药量每日 1-2	暂停普特利单抗给药，直至不良事件转

	的肺部浸润	理学评价	mg/kg	为 1 级或更佳
			重度（3 级）或更严重：大剂量糖皮质激素（每日静脉注射 2-4 mg/kg 甲基强的松龙）配合抗生素；病人住院治疗；与呼吸科医师会诊；可以考虑施用英夫利昔单抗给予额外的免疫抑制	永久性终止普特利单抗治疗
结肠炎	水泻，粪便附着粘液或便血，腹痛<br><br>恶心呕吐，脱水，腹膜损伤迹象，肠穿孔	• 便检<br>• 内窥镜检或结肠镜检排查可能的感染<br>• 活组织检查排查其他可能的致病原	轻度（1 级）腹泻：止泻药治疗，口服补液疗法，补充电解质	继续给药
			中度（2 级）腹泻：施用糖皮质激素强的松或同类药，给药量每日 1-2 mg/kg	暂停普特利单抗给药，直至不良事件转为 1 级或更佳
			重度（3 级）腹泻：大剂量糖皮质激素（每日静脉注射 2-4 mg/kg 甲基强的松龙）配合抗生素；激素难治型腹泻可以考虑施用英夫利昔单抗	暂停普特利单抗给药，直至不良事件转为 1 级或更佳
			复发重度（3 级）或危及生命（4 级）：大剂量糖皮质激素（每日静脉注射 2-4 mg/kg 甲基强的松龙）配合抗生素；激素难治型腹泻可以考虑施用英夫利昔单抗；病人住院治疗	永久性终止普特利单抗治疗
			如有疑似肠穿孔症状，应停用激素并且考虑手术；病人住院治疗；不能施用英夫利昔单抗	永久性终止普特利单抗治疗
肝炎	有症状的：发烧，倦怠，恶心，腹痛，肝肿大	• 化学诊断：针对肝酶水平升高<br>• 血清测试：排查病毒性肝炎（包	• 中度（2 级）：施用糖皮质激素强的松或同类药，给药量每日 1-2 mg/kg	暂停普特利单抗给药，直至不良事件转为 1 级或更佳

	无症状的：肝功能指标升高（肝酶水平，胆红素水平）	括甲型和乙型肝炎），巨细胞病毒和EB病毒<br>• 肝脏超声诊断<br>• 活组织检查或放射影像评价以区分其他导致肝损伤的致病原，例如肝部肿瘤进展，感染以及摄入其他药物或酒精导致的影响	• 针对转移性肝癌或原发性肝细胞癌的受试者，如在参与试验时已被诊断出中度（2 级）AST/ALT 升高（高于基线 50%或以上），则暂停普特利单抗给药	
			• 重度（3 级）或更严重：大剂量糖皮质激素（每日静脉注射 2-4 mg/kg 甲基强的松龙）；48-72 小时后如无改善则考虑施用其他免疫抑制剂（吗替麦考酚酯）；病人住院治疗；与肝病专科医师会诊<br>• 针对转移性肝癌或原发性肝细胞癌的受试者，如在参与试验时已被诊断出中度（2 级）AST/ALT 升高（高于基线 50%或以上且持续一周或更久），则永久终止普特利单抗给药<br>• 血清总胆红素>正常水平上限 3 倍以上	永久性终止普特利单抗治疗
垂体炎	对非甾体抗炎药或其他镇痛药耐受的头痛；虚弱；倦怠，增重或失重；情绪或行为举止异常；低血压；电解质紊乱；腹痛，性欲减退；肾上腺危象；性腺功能减退	• 临床化学检验<br>• 内分泌实验室检测，促肾上腺皮质激素，甲状腺功能检查<br>• 脑部核磁共振：针对有显著增大特征的脑垂体	中度（2 级）且无肾上腺危象：大剂量糖皮质激素（每日 1-2 mg/kg 甲基强的松龙或同类药）；启动激素替代疗法；与内分泌医师会诊	暂停普特利单抗给药，直至不良事件转为 1 级或更佳
			≥3 级或出现肾上腺危象：大剂量糖皮质激素（每日 1-2 mg/kg 甲基强的松龙或同类药）；与内分泌医师会诊	永久性终止普特利单抗治疗

肾炎	尿血，外周性水肿，血清肌酐水平升高	• 临床化学检验<br>• 尿检<br>• 活组织检查（如有必要）	中度（2 级）：施用糖皮质激素强的松或同类药，给药量每日 1-2 mg/kg	暂停普特利单抗给药，直至不良事件转为 1 级或更佳
			重度（3 级）或更严重：施用糖皮质激素强的松或同类药，给药量每日 1-2 mg/kg	永久性终止普特利单抗治疗
甲状腺功能减退/甲状腺功能亢进	典型的无症状的不良事件，依靠实验室检测发现	依据甲状腺素 T3，T4 及促甲状腺激素的检测结果	中度（2 级）以下无症状的甲状腺功能减退：甲状腺素替代疗法	继续给药
			中度（2 级）以下无症状的甲状腺功能亢进：考虑使用 beta 受体阻滞剂	继续给药
			重度（3 级）或更严重的甲状腺功能减退：替代疗法	暂停普特利单抗给药，直至不良事件转为≤1 级或基线水平
			在有效管理下仍持续 6 周或更久的重度（3 级）甲状腺功能亢进：施用糖皮质激素强的松或同类药，给药量每日 1-2 mg/kg	暂停普特利单抗给药，直至不良事件转为 1 级或更佳
			危及生命（4 级）的甲状腺功能亢进	永久性终止普特利单抗治疗
皮炎	斑丘疹或红皮症，瘙痒症，皮肤溃疡，大疱性皮炎，史-约综合征	• 在确认其他可能的致病原之前，应视为免疫介导<br>• 可对皮肤活组织切片进行病理学评价以排查可能的致病原	中度（2 级）或更佳（≤ 30%体表面积）：局部外用糖皮质激素和口服非处方抗组胺药，若 7 日无改善可使用系统性糖皮质激素	继续给药
			重度（3 级，＞30%体表面积）：施用糖皮质激素强的松或同类药，给药量每日 1-2 mg/kg	暂停普特利单抗给药，直至不良事件转为 2 级或更佳
			危及生命（4 级，例如：史-约综合征，中毒性表皮坏死，全层性皮肤溃	永久性终止普特利单抗治疗

			疡、坏死或出血）：大剂量糖皮质激素（每日 2-4 mg/kg 甲基强的松龙或同类药）；病人住院治疗；与皮肤科医师会诊	
神经肌肉毒性	外周感觉神经病变，肌无力，格林巴利综合征，横断性脊髓炎，重症肌无力	• 理学检查：感官变化，腱反射缺失<br>• 神经影像检测，神经传导测试<br>• 神经/肌肉活组织检查	中度（2 级）或更佳：施用糖皮质激素强的松或同类药，给药量每日 1-2 mg/kg	暂停普特利单抗给药，直至不良事件转为 1 级或更佳
			重度（3 级）或更严重：大剂量糖皮质激素（每日 2-4 mg/kg 甲基强的松龙或同类药）	永久性终止普特利单抗治疗
脑炎	头痛，发热，疲劳或虚弱，意识模糊，记忆力障碍，嗜睡，幻觉，癫痫，颈强直	• 神经影像检测，脑部核磁共振造影<br>• 腰椎穿刺	1 级（无症状或轻微症状）	继续普特利单抗给药
			2 级（首次出现的中重度症状或体征）：与神经科医师会诊，根据治疗指南进行症状针对性治疗。评估感染或其它导致神经病变的病因。	暂停普特利单抗给药，直至恢复至基线水平
			3 级以上（免疫性脑炎，无其它病因）：与神经科医师会诊，施用糖皮质激素（每日 1-2 mg/kg 强的松或同类药）配合抗生素。若无效可考虑其它免疫抑制剂。	永久性终止普特利单抗治疗
眼毒性	光敏性，眼部疼痛或干涩，视力模糊，葡萄膜炎，虹膜炎，表层巩膜炎	• 眼科检查	中度（2 级）或更佳：局部外用糖皮质激素（1% 的泼尼松龙）	暂停普特利单抗给药，直至不良事件转为 1 级或更佳；在局部外用或系统性糖皮质激素治疗下，如 14 天内未转为 1 级或更佳，永久性终止普特利单抗治疗
			重度（3 级）或更严重：大剂量糖皮质激素（每	永久性终止普特利单抗治疗

			日 2-4 mg/kg 甲基强的松龙或同类药）	
心肌炎	胸痛，心悸，呼吸困难，外周性水肿	• 心肌酶<br>• 心电图<br>• 超声心动图<br>• 心血管磁共振检查<br>• 心内膜心肌活检	轻度（1 级，无症状，有实验室或影像学异常）：持续监测心肌酶和心电图（2-3 天一次）。	若两周内恢复到基线水平，则继续给药，若没有恢复，则暂停给药，并请心内科医师会诊。
			中度（2 级）或更严重（有临床症状）：入院观察，持续监测心肌酶、心电图、超声心动图等，请心内科医师会诊。可行心血管磁共振检查，并考虑心内膜心肌活检。应用糖皮质激素治疗。如活检证实严重心肌炎，应考虑免疫调节剂如抗胸腺细胞球蛋白（推荐）/英孚利昔（未出现心衰的情况）/麦考酚酸吗乙酯/他克莫司。	永久性终止普特利单抗治疗
其他免疫介导的不良反应例如关节炎，胰腺炎，溶血性贫血，肾上腺功能不全，肌无力综合征和横纹肌溶解			中度（2 级）	暂停普特利单抗给药，直至不良事件转为 1 级或更佳
			重度（3 级）或更严重	永久性终止普特利单抗治疗

附件 4 体重指数（BMI）计算公式

体重指数（BMI）=体重/身高的平方

体重单位为千克（Kg），身高单位为米（m）。

附件 5 EORCT QLQ C30（生活质量评分）量表

CHINESE MANDARIN (CHINA)

EORTC QLQ-C30 (V3)

我们想了解有关您和您的健康的一些情况，请您亲自回答下面所有问题，这里的答案并无“对”与“不对”之分，只要求在最能反映您情况的那个数字上画圈。您所提供的资料我们将会严格保密。

请填上您的姓名首位字母：___________

出生日期： ___ 年___月___日

今天日期： ___ 年___月___日

	没有	有点	相当	非常
1．您从事一些费力的活动有困难吗,比如说提很重的购物袋或手提箱？	1	2	3	4
2．长距离行走对您来说有困难吗？	1	2	3	4
3．户外短距离行走对您来说有困难吗？	1	2	3	4
4．您白天需要呆在床上或椅子上吗？	1	2	3	4
5．您在吃饭、穿衣、洗澡或上厕所时需要他人帮忙吗？	1	2	3	4

**在过去的一星期内：**	没有	有点	相当	非常
6．您在工作和日常活动中是否受到限制？	1	2	3	4
7．您在从事您的爱好或休闲活动时是否受到限制？	1	2	3	4
8．您有气短吗？	1	2	3	4
9．您有疼痛吗？	1	2	3	4
10．您需要休息吗？	1	2	3	4
11．您睡眠有困难吗？	1	2	3	4
12．您觉得虚弱吗？	1	2	3	4
13．您食欲不振（没有胃口）吗？	1	2	3	4
14．您觉得恶心吗？	1	2	3	4
15．您有呕吐吗？	1	2	3	4
16．您有便秘吗？	1	2	3	4

请接下页

在过去的一星期内：	没有	有点	相当	非常
17．您有腹泻吗？	1	2	3	4
18．您觉得累吗？	1	2	3	4
19．疼痛影响您的日常活动吗？	1	2	3	4
20．您集中精力做事有困难吗，如读报纸或看电视？	1	2	3	4
21．您觉得紧张吗？	1	2	3	4
22．您觉得忧虑吗？	1	2	3	4
23．您觉得脾气急躁吗？	1	2	3	4
24．您觉得压抑（情绪低落）吗？	1	2	3	4
25．您感到记忆困难吗？	1	2	3	4
26．您的身体状况或治疗影响您的家庭生活吗？	1	2	3	4
27．您的身体状况或治疗影响您的社交活动吗？	1	2	3	4
28．您的身体状况或治疗使您陷入经济困难吗？	1	2	3	4

对下列问题，请在 1-7 之间选出一个最适合您的数字并画圈。

29．您如何评价在过去一星期内您总的健康情况？

1　2　3　4　5　6　7

非常差　　　　　　　　　　非常好

30．您如何评价在过去一星期内您总的生命质量？

1　2　3　4　5　6　7

非常差　　　　　　　　　　非常好

© Copyright 1995 EORTC Study Group on Quality of Life. All rights reserved. Version 3.0

附件 6 欧洲癌症研究组织(EORTC)头颈癌生存质量问卷(QLQ H&N35)

EORTC QLQ - H&N35

Patients sometimes report that they have the following symptoms or problems. Please indicat extent to which you have experienced these symptoms or problems during the past week. P answer by circling the number that best applies to you.

During the past week:	Not at all	A little	Quite a bit	Very mucl
31. Have you had pain in your mouth?	1	2	3	4
32. Have you had pain in your jaw?	1	2	3	4
33. Have you had soreness in your mouth?	1	2	3	4
34. Have you had a painful throat?	1	2	3	4
35. Have you had problems swallowing liquids?	1	2	3	4
36. Have you had problems swallowing pureed food?	1	2	3	4
37. Have you had problems swallowing solid food?	1	2	3	4
38. Have you choked when swallowing?	1	2	3	4
39. Have you had problems with your teeth?	1	2	3	4
40. Have you had problems opening your mouth wide?	1	2	3	4
41. Have you had a dry mouth?	1	2	3	4
42. Have you had sticky saliva?	1	2	3	4
43. Have you had problems with your sense of smell?	1	2	3	4
44. Have you had problems with your sense of taste?	1	2	3	4
45. Have you coughed?	1	2	3	4
46. Have you been hoarse?	1	2	3	4
47. Have you felt ill?	1	2	3	4
48. Has your appearance bothered you?	1	2	3	4

SPECIMEN

Please go on to the next page

ENGLISH

During the past week:		Not at all	A little	Quite a bit	Very much
49.	Have you had trouble eating?	1	2	3	4
50.	Have you had trouble eating in front of your family?	1	2	3	4
51.	Have you had trouble eating in front of other people?	1	2	3	4
52.	Have you had trouble enjoying your meals?	1	2	3	4
53.	Have you had trouble talking to other people?	1	2	3	4
54.	Have you had trouble talking on the telephone?	1	2	3	4
55.	Have you had trouble having social contact with your family?	1	2	3	4
56.	Have you had trouble having social contact with friends?	1	2	3	4
57.	Have you had trouble going out in public?	1	2	3	4
58.	Have you had trouble having physical contact with family or friends?	1	2	3	4
59.	Have you felt less interest in sex?	1	2	3	4
60.	Have you felt less sexual enjoyment?	1	2	3	4

During the past week:		No	Yes
61.	Have you used pain-killers?	1	2
62.	Have you taken any nutritional supplements (excluding vitamins)?	1	2
63.	Have you used a feeding tube?	1	2
64.	Have you lost weight?	1	2
65.	Have you gained weight?	1	2

SPECIMEN

附件 7 华盛顿大学生存质量问卷第 4 版（UW-QOL，V4.0)

该问卷考察你过去 7 天里的生存质量情况，请回答所有问题，在符合你情况的方框内划勾。
1. 疼痛
□我根本没有疼痛
□我有轻的疼痛，但不需要药物治疗
□我有中等程度疼痛，需要按时服药（如可待因或非麻醉剂）
□我有严重疼痛，必须用麻醉剂（如吗啡）才能控制
□我有极严重疼痛，麻醉剂（如吗啡）也不能控制
2. 外貌
□我的外貌没有改变
□我的外貌改变很轻微
□我的外貌改变使我烦恼，但我仍能保持活力
□我感到严重毁容并因此限制了我的活力
□由于我的外貌，我不能与人相处
3. 活力
□我与以往一样活跃
□我有时不能保持以往的行动速度，但并不常有
□我经常觉得累，尽管我也外出，但我放慢了活动的速度
□我不外出，因为我没有力气
□我经常卧床或坐在椅子上，不能离开家门
4. 娱乐
□我的娱乐没有室内或户外限制
□我有一些事情不能做，但我仍能外出享受生活
□我多次希望能更多外出，但不能如愿以偿
□我所能做的受到了严重限制，大部分时候我呆在家里看电视
□我完全不能享受生活
5. 吞咽
□我能像以前那样咽食物
□我不能咽下某些固体食物
□我只能进流食
□我不能吞咽，因为咽食物时会呛住
6. 咀嚼
□我能像以前那样咀嚼食物
□我能吃软的食物，但咬不动某些食物
□我连软的食物也吃不了
7. 语言
□我说话和以前没有区别
□我说某些字时觉得费劲，但打电话时能被别人听懂
□只有家人和朋友能听懂我说什么
□我说话别人完全听不懂
8. 肩功能
□我的肩膀没有任何问题
□我的肩膀僵硬，但不影响我的活动或力气
□我的肩膀疼痛或无力，使我不得不改变工作（或家务劳动）
□我因为肩膀问题而不能工作（或家务劳动）
9. 味觉
□我能正常品尝食物
□我能正常品尝大部分食物
□我只能品尝一些食物

□我完全不能品尝食物
10. 唾液
□我的唾液正常
□我的唾液略少，但已足够
□我的唾液太少了
□我完全没有唾液
11. 情绪
□我的情绪良好，不受癌症的影响
□我的情绪总的来说很好，偶尔受到癌症的影响
□因为癌症，我的情绪说不上好，但也不沮丧
□因为疾病，我有一些沮丧
□因为疾病，我极度沮丧
12. 焦虑
□我对所患的癌症不焦虑
□我对所患的癌症有一点焦虑
□我对所患的癌症感到焦虑
□我对所患的癌症极为焦虑
选择在过去 7 天里对你影响最大的 3 个项目：
□疼痛 □吞咽 □味觉
□外貌 □咀嚼 □唾液
□活力 □发音 □情绪
□娱乐 □肩功能 □焦虑

综合问题

1．与您患疾病之前的 1 个月相比，你如何评价你现在的健康相关生存质量？
□比以前好多了
□比以前好一点
□和以前差不多
□比以前差一些
□比以前差远了
2．总的说来，你认为你过去 7 天的健康相关生存质量怎样？
□ 极 好 □ 比 较 好 □ 一 般 □ 比 较 差 □极差
3．总体生存质量不仅包括身体健康和心理健康，还包括一些其他因素，如家庭、朋友、精神、个人娱乐活动等一些对你享受生活有重要意义的因素。考虑对你的个人生活有影响的所有事情，评价过去 7 天里你的总体生存质量：
□ 极 好 □ 比 较 好 □ 一 般 □ 比 较 差□极差
请叙述在我们的问题中没有充分涉及、但对你的生存质量很重要的其他问题（医学的或非医学的），如果必要，可附加额外的纸张。
