# Supplementary material for: R0 resection following neoadjuvant PD-1 inhibition with paclitaxel-cisplatin is associated with improved overall survival in locally advanced and recurrent oral squamous cell carcinoma: multicenter retrospective cohort study
Source: Ann Med. 2026 Oct 29;58(1):2744077. doi: 10.1080/07853890.2026.2744077 (PMC13721807; doi:10.1080/07853890.2026.2744077)
Supplement: Supplementary file 2 [file IANN_A_2744077_SM3824.docx]

Supplementary materials

Neoadjuvant PD-1 Inhibition Plus Paclitaxel and Cisplatin Followed by R0 Resection Improves Overall Survival in Locally Advanced and Recurrent Oral Squamous Cell Carcinoma

Qiwei Wang^1*^, Yan Liu^1*^, Liang Zuo^1^, Shuichao Gao^1^, Jiaming Chen^1^, Lei Xue^2^, Ping Li^3^, Leixiao Qing^4^, Jingguan Lin^5^, Yaqian Han^6^, Hekun Jin^6^, Yujuan Zhou^6^, Hao Tian^1#^,

# ^1^MD, Department of head and neck surgery, Hunan Cancer Hospital, The Affiliated Cancer Hospital of Xiangya school of medicine, Central South University, Changsha, Hunan province, China;

# ^2^MD, Department of pathology, Hunan Cancer Hospital, The Affiliated Cancer Hospital of Xiangya school of medicine, Central South University, Changsha, Hunan province, China

# ^3^MD, Department of Maxillofacial-Head and Neck Surgery, Liuyang Hospital of Traditional Chinese Medicine, Changsha, Hunan province, China

# ^4^MD, Department of Otolaryngology, Liling Hospital of Traditional Chinese Medicine, Zhuzhou, Hunan province, China

# ^5^MD, Hunan Key Laboratory of Cancer Metabolism, Hunan Cancer Hospital, The Affiliated Cancer Hospital of Xiangya school of medicine, Central South University, Changsha, Hunan province, China

# ^6^MD, Department of Head and Neck Radiotherapy, Hunan Cancer Hospital, The Affiliated Cancer Hospital of Xiangya school of medicine, Central South University, Changsha, Hunan province, China

# ^#^Corresponding author: Hao Tian, MD, Department of head and neck surgery, Hunan Cancer Hospital, The Affiliated Cancer Hospital of Xiangya school of medicine, Central South University, Changsha, Hunan province, China; Email: tianhao@hnca.org.cn

# * These authors contribute equally

The model includes only pretreatment baseline covariates. Surgery, radiotherapy, and the post-treatment response category were excluded by design; surgery and radiotherapy were instead analyzed separately as time-dependent exposures (Table 3). T0–T2 were collapsed into a single reference category because of small numbers (T0, n=4; T1, n=1; T2, n=12). Recurrent disease denotes recurrence documented before the initiation of neoadjuvant therapy (a baseline characteristic), not relapse after treatment. The proportional hazards assumption was assessed using Schoenfeld residuals; no covariate showed evidence of violation (all covariate-specific tests, P>0.62). HR, hazard ratio; CI, confidence interval. (Supplementary Table S2).

**Table S2 Multivariable Cox regression analysis**

| **Variable** | **HR** | **P value** | **Lower 95%CI** | **Upper 95%CI** |
| --- | --- | --- | --- | --- |
| **Recurrent disease** |  |  |  |  |
| Yes VS No | 3.02 | **<0.001** | 1.71 | 5.32 |
| **T_stage** |  |  |  |  |
| T3 VS T0-T2 | 0.81 | 0.673 | 0.3 | 2.19 |
| T4 VS T0-T2 | 0.92 | 0.864 | 0.35 | 2.39 |
| **N_stage** |  |  |  |  |
| N1 VS N0 | 0.75 | 0.434 | 0.36 | 1.54 |
| N2 VS N0 | 1.19 | 0.615 | 0.60 | 2.35 |
| N3 VS N0 | 1.65 | 0.39 | 0.53 | 5.15 |
| **Tumor site** |  |  |  |  |
| Gingiva_palate VS Buccal | 0.82 | 0.625 | 0.38 | 1.80 |
| Tongue VS Buccal | 0.64 | 0.138 | 0.35 | 1.16 |
