# Supplementary material for: R0 resection following neoadjuvant PD-1 inhibition with paclitaxel-cisplatin is associated with improved overall survival in locally advanced and recurrent oral squamous cell carcinoma: multicenter retrospective cohort study
Source: Ann Med. 2026 Oct 29;58(1):2744077. doi: 10.1080/07853890.2026.2744077 (PMC13721807; doi:10.1080/07853890.2026.2744077)
Supplement: Supplementary file 3 [file IANN_A_2744077_SM3825.docx]

Supplementary materials

Neoadjuvant PD-1 Inhibition Plus Paclitaxel and Cisplatin Followed by R0 Resection Improves Overall Survival in Locally Advanced and Recurrent Oral Squamous Cell Carcinoma

Qiwei Wang^1*^, Yan Liu^1*^, Liang Zuo^1^, Shuichao Gao^1^, Jiaming Chen^1^, Lei Xue^2^, Ping Li^3^, Leixiao Qing^4^, Jingguan Lin^5^, Yaqian Han^6^, Hekun Jin^6^, Yujuan Zhou^6^, Hao Tian^1#^,

# ^1^MD, Department of head and neck surgery, Hunan Cancer Hospital, The Affiliated Cancer Hospital of Xiangya school of medicine, Central South University, Changsha, Hunan province, China;

# ^2^MD, Department of pathology, Hunan Cancer Hospital, The Affiliated Cancer Hospital of Xiangya school of medicine, Central South University, Changsha, Hunan province, China

# ^3^MD, Department of Maxillofacial-Head and Neck Surgery, Liuyang Hospital of Traditional Chinese Medicine, Changsha, Hunan province, China

# ^4^MD, Department of Otolaryngology, Liling Hospital of Traditional Chinese Medicine, Zhuzhou, Hunan province, China

# ^5^MD, Hunan Key Laboratory of Cancer Metabolism, Hunan Cancer Hospital, The Affiliated Cancer Hospital of Xiangya school of medicine, Central South University, Changsha, Hunan province, China

# ^6^MD, Department of Head and Neck Radiotherapy, Hunan Cancer Hospital, The Affiliated Cancer Hospital of Xiangya school of medicine, Central South University, Changsha, Hunan province, China

# ^#^Corresponding author: Hao Tian, MD, Department of head and neck surgery, Hunan Cancer Hospital, The Affiliated Cancer Hospital of Xiangya school of medicine, Central South University, Changsha, Hunan province, China; Email: tianhao@hnca.org.cn

# * These authors contribute equally

**Supplementary Figure S4. Landmark analyses accounting for immortal time bias.** Kaplan–Meier estimates of overall survival after exclusion of patients who died or were censored before the landmark, with the time origin reset: (A) surgery versus non-surgery, 6-month landmark; (B) radiotherapy versus no radiotherapy, 9-month landmark; (C) postoperative radiotherapy versus surgery alone in surgical patients, 9-month landmark; (D) surgery versus non-surgery in recurrent disease, 6-month landmark. HRs and 95% CIs were estimated using Cox proportional hazards models from the landmark time.
