# Supplementary material for: R0 resection following neoadjuvant PD-1 inhibition with paclitaxel-cisplatin is associated with improved overall survival in locally advanced and recurrent oral squamous cell carcinoma: multicenter retrospective cohort study
Source: Ann Med. 2026 Oct 29;58(1):2744077. doi: 10.1080/07853890.2026.2744077 (PMC13721807; doi:10.1080/07853890.2026.2744077)
Supplement: Supplementary file 6 [file IANN_A_2744077_SM3830.docx]

Supplementary materials

Neoadjuvant PD-1 Inhibition Plus Paclitaxel and Cisplatin Followed by R0 Resection Improves Overall Survival in Locally Advanced and Recurrent Oral Squamous Cell Carcinoma

Qiwei Wang^1*^, Yan Liu^1*^, Liang Zuo^1^, Shuichao Gao^1^, Jiaming Chen^1^, Lei Xue^2^, Ping Li^3^, Leixiao Qing^4^, Jingguan Lin^5^, Yaqian Han^6^, Hekun Jin^6^, Yujuan Zhou^6^, Hao Tian^1#^,

# ^1^MD, Department of head and neck surgery, Hunan Cancer Hospital, The Affiliated Cancer Hospital of Xiangya school of medicine, Central South University, Changsha, Hunan province, China;

# ^2^MD, Department of pathology, Hunan Cancer Hospital, The Affiliated Cancer Hospital of Xiangya school of medicine, Central South University, Changsha, Hunan province, China

# ^3^MD, Department of Maxillofacial-Head and Neck Surgery, Liuyang Hospital of Traditional Chinese Medicine, Changsha, Hunan province, China

# ^4^MD, Department of Otolaryngology, Liling Hospital of Traditional Chinese Medicine, Zhuzhou, Hunan province, China

# ^5^MD, Hunan Key Laboratory of Cancer Metabolism, Hunan Cancer Hospital, The Affiliated Cancer Hospital of Xiangya school of medicine, Central South University, Changsha, Hunan province, China

# ^6^MD, Department of Head and Neck Radiotherapy, Hunan Cancer Hospital, The Affiliated Cancer Hospital of Xiangya school of medicine, Central South University, Changsha, Hunan province, China

# ^#^Corresponding author: Hao Tian, MD, Department of head and neck surgery, Hunan Cancer Hospital, The Affiliated Cancer Hospital of Xiangya school of medicine, Central South University, Changsha, Hunan province, China; Email: tianhao@hnca.org.cn

# * These authors contribute equally

Postoperative radiotherapy followed a response-adapted risk-stratification protocol: patients achieving pCR or pPR without high-risk features received 60 Gy/30 fractions, whereas escalation to 66 Gy with concurrent weekly cisplatin was indicated by extranodal extension, poor pathological response (pSD or PD), perineural invasion, or lymph vascular invasion (Supplementary Table S1).

**Table S1 Pathological grading characteristics**

| **Patient Group** | **Number of Cases** | **Pathological Features / Notes** | **Radiotherapy Regimen** |
| --- | --- | --- | --- |
| **Non-Surgical Treatment** | All patients | - | 66 Gy + concurrent cisplatin: 100 mg/m² every 3 weeks |
| **Postoperative Radiotherapy** | 48 | - | - |
| pCR | 13 | Pathological complete response | 60 Gy/30f |
| pPR | 24 | Partial pathological response | 23 patients: 60 Gy; 30f  1 patient (ENE+): 66 Gy/33f + weekly cisplatin: 40 mg/m² |
| PD | 1 | Perineural and vascular invasion | 66 Gy/33f + weekly cisplatin: 40 mg/m² |
| pSD | 10 | Perineural and vascular invasion | 66 Gy/33f + weekly cisplatin: 40 mg/m² |
